# Comparison of models to predict incident chronic liver disease: a systematic review and external validation in Chinese adults

**DOI:** 10.1186/s12916-024-03754-9

**Published:** 2024-12-31

**Authors:** Xue Cong, Shuyao Song, Yingtao Li, Kaiyang Song, Cameron MacLeod, Yujie Cheng, Jun Lv, Canqing Yu, Dianjianyi Sun, Pei Pei, Ling Yang, Yiping Chen, Iona Millwood, Shukuan Wu, Xiaoming Yang, Rebecca Stevens, Junshi Chen, Zhengming Chen, Liming Li, Christiana Kartsonaki, Yuanjie Pang, on behalf of the China Kadoorie Biobank Collaborative Group

**Affiliations:** 1 Department of Epidemiology & Biostatistics, School of Public Health, Peking University, 38 Xueyuan Road, Beijing, 100191 China; 2 Medical Sciences Division, University of Oxford, Oxford, OX3 9DU UK; 3 Center for Public Health and Epidemic Preparedness & Response, Peking University, Beijing, 100191 China; 4 Key Laboratory of Epidemiology of Major Diseases (Peking University), Ministry of Education, Beijing, 100191, China; 5 Clinical Trial Service Unit & Epidemiological Studies Unit (CTSU), Nuffield Department of Population Health, University of Oxford, Old Road Campus, Oxford, OX3 7LF UK; 6 Medical Research Council Population Health Research Unit at the University of Oxford, Oxford, OX3 7LF UK; 7 Meilan Center for Disease Control and Prevention, Haikou, 570100 China; 8 China National Center for Food Safety Risk Assessment, Beijing, 100022 China

**Keywords:** Risk prediction, Chronic liver disease, Hepatocellular carcinoma, Chinese, Systematic review, External validation

## Abstract

**Background:**

Risk prediction models can identify individuals at high risk of chronic liver disease (CLD), but there is limited evidence on the performance of various models in diverse populations. We aimed to systematically review CLD prediction models, meta-analyze their performance, and externally validate them in 0.5 million Chinese adults in the China Kadoorie Biobank (CKB).

**Methods:**

Models were identified through a systematic review and categorized by the target population and outcomes (hepatocellular carcinoma [HCC] and CLD). The performance of models to predict 10-year risk of CLD was assessed by discrimination (C-index) and calibration (observed vs predicted probabilies).

**Results:**

The systematic review identified 57 articles and 114 models (28.4% undergone external validation), including 13 eligible for validation in CKB. Models with high discrimination (C-index ≥ 0.70) in CKB were as follows: (1) general population: Li-2018 and Wen 1–2012 for HCC, CLivD score (non-lab and lab) and dAAR for CLD; (2) hepatitis B virus (HBV) infected individuals: Cao-2021 for HCC and CAP-B for CLD. In CKB, all models tended to overestimate the risk (O:E ratio 0.55–0.94). In meta-analysis, we further identified models with high discrimination: (1) general population (C-index ≥ 0.70): Sinn-2020, Wen 2–2012, and Wen 3–2012 for HCC, and FIB-4 and Forns for CLD; (2) HBV infected individuals (C-index ≥ 0.80): RWS-HCC and REACH-B IIa for HCC and GAG-HCC for HCC and CLD.

**Conclusions:**

Several models showed good discrimination and calibration in external validation, indicating their potential feasibility for risk stratification in population-based screening programs for CLD in Chinese adults.

**Supplementary Information:**

The online version contains supplementary material available at 10.1186/s12916-024-03754-9.

## Background

Chronic liver disease (CLD), encompassing mainly liver cancer and cirrhosis, affected 1.70 billion people globally in 2021 [1]. According to the Global Burden of Disease Study (GBD), the disease burden of CLD is high in China, with 0.39 billion cases and 0.33 million deaths in 2021. With the universal coverage of hepatitis B virus (HBV) vaccination and Westernized lifestyles, the leading cause of CLD in China shifted from HBV to non-alcoholic fatty liver disease (NAFLD), which accounted for ~ 40% prevalent cases in 1990 and ~ 70% in 2019. Due to the asymptomatic progression, CLD is often diagnosed in advanced stages and has a poor prognosis [2].

Screening strategies for CLD are imperative because disease surveillance contributes to early diagnosis and overall survival [3, 4]. Screening strategies can be divided into individual-based (i.e., centering on the high-risk individuals) and population-wide approaches. The American Association for the Study of Liver Diseases (AASLD) [5] and European Association for the Study of the Liver (EASL) [6] recommend ultrasound and alpha-fetoprotein (AFP) tests to high-risk populations including patients with cirrhosis and non-cirrhotic chronic hepatitis B (CHB) as screening strategies for liver cancer. China adopts a similar approach [7]. However, because of the suboptimal sensitivity, dependency on operator skills, poor adherence, and limited accessibility of imaging modalities [8], non-invasive biomarkers and risk prediction models have emerged as promising risk stratification tools for population screening, which optimize resources and have the potential to improve the detection and prognosis of CLD [9].

Currently, international consensuses and expert views have recommended non-patented blood tests (e.g., FIB-4 or NAFLD fibrosis score) as screening tests for patients with CLD risk factors [10–12]. However, despite the satisfactory C-index (between 0.53 and 0.80) [13], the inaccessibility in the community setting hinders their generalizability as first-line tests [14, 15]. Recent studies have focused on developing models incorporating more accessible non-laboratory parameters, such as lifestyle factors and family history. Despite the development of numerous prediction models for CLD, significant gaps remain in model implementation, including inadequate validation across diverse regions and populations [10]. Furthermore, there is a lack of comprehensive comparison of CLD models across populations with different CLD etiologies, particularly in China, where the etiology differs importantly from Western countries.

Therefore, our objective was to systematically review published CLD prediction models and externally validate them using the China Kadoorie Biobank (CKB), one of the largest and geographically diverse prospective cohort studies in China. We also conducted a meta-analysis to compare model performance across populations in both published cohort studies and CKB.

## Methods

### Systematic literature search and identification of published models

A systematic search was conducted in the PubMed and Embase databases up to October 14, 2022. The search terms included MeSH terms in PubMed and Emtree terms in Embase as well as free-text terms. The following terms were used as index terms or free-text words: “hepatocellular carcinoma,” “liver cancer,” “chronic liver disease,” “severe liver disease,” and “risk prediction,” among other related terms. We included original research articles, systematic reviews, and conference abstracts. The references of systematic reviews were manually reviewed to identify potentially missing studies. The complete search strategy for the databases is provided in Additional file 1: Method S1.

The articles were included based on the following criteria: (1) focused on the development, update, or validation of prediction models, or the comparison of existing models; (2) included predictors involving but not limited to lifestyle and clinical risk factors; (3) designed as prospective cohorts, retrospective cohorts, case-cohort studies, or nested case–control studies; (4) included outcomes related to CLD, such as liver cancer, cirrhosis, and other liver diseases caused by various etiologies (e.g., NAFLD, alcoholic liver disease [ALD], and viral hepatitis); and (5) reported performance measures of predictive ability, including but not limited to the area under the ROC curve (AUC) or C-statistic.

We excluded ecological studies (i.e., conducted at the population level), studies comprising hospital patients who underwent CLD-related procedures (e.g., hepatectomy, liver transplantation) or received specific antiviral therapies (e.g., entecavir, ribavirin), studies focused solely on prognostic models, studies not published in English, and studies categorized as narrative reviews, letters, editorials, or commentaries.

After removing duplicate articles, two independent reviewers (XC and YC) conducted separate title and abstract screening, retrieving the full text when necessary. Discrepancies were resolved through discussion, and a third reviewer (YP) was consulted. After the initial screening, three reviewers (XC, KS, and CM) independently conducted full-text screening and data extraction to determine the final inclusion of eligible articles. The reasons for exclusion were recorded for each article during the full-text screening. To ensure the accuracy of the database, the three reviewers re-checked any discrepancies in data extraction and reviewed the reference lists of all eligible articles to identify any missed studies. The research protocol has been registered and approved in the PROSPERO international prospective register (ID: CRD42022374724). The risk of bias was assessed according to PROBAST (Prediction model Risk Of Bias Assessment Tool) [16].

### Data extraction

Data extraction was based on the guidelines of the Preferred Reporting Items for Systematic Reviews and Meta-Analyses (PRISMA) statement [17]. Information extracted included citation details (e.g., authors, publication date, region), study design and methods, study population, sample size, model name, model formula, included variables, measures of predictive ability, recruitment years of participants, duration of follow-up, measurement outcomes, and their corresponding International Classification of Diseases (ICD) codes, as well as outcome measurement methods. The information was extracted from each eligible risk prediction model to perform external validation. When multiple validation studies existed, a meta-analysis was performed to summarize the evidence to support and compare prediction models in a particular field according to TRIPOD-SRMA [18].

If published models had been updated regarding the predictors or coefficients, the updated data were extracted. For models providing absolute risk, the following data were also extracted to evaluate the model’s calibration: age-specific incidence rates, age-specific mortality rates, attributable risk, survival functions, mean values of risk factors in the cohort, and risk scores estimated at the mean values of all predictors.

### Validation cohorts

The models were externally validated according to the TRIPOD (Transparent Reporting of a multivariable prediction model for Individual Prognosis Or Diagnosis) guidelines [19]. Details of the CKB study and methods for external validation are reported in Additional file 1: Method S2 [20, 21].

### Model predictors and outcomes

During the external validation stage, we first attempted to match the predictors of the original model with the available variables in CKB. When a direct match could not be achieved, a proxy variable was defined as closely as possible to the original predictor. The measurement and definition of CKB variables are described in detail in Additional file 1: Method S3. If none of the above situations could be achieved, the model was excluded.

All eligible models were classified according to the target population. General population included population-based cohorts with no specific restrictions on CLD risk factors. HBV infected individuals included patients with CHB and individuals with positive serum hepatitis B surface antigen (HBsAg). HCV infected individuals included patients with chronic hepatitis C (CHC), individuals with positive serum anti-hepatitis C virus antibody (Anti-HCV Ab), and individuals with positive serum HCV RNA. Patients with NAFLD, diabetes, and individuals with high CLD risk (including diabetes, obesity, and high alcohol consumption) were included.

Outcomes of the prediction models were categorized into hepatocellular carcinoma (HCC) and CLD. HCC was defined by the ICD-10 code C22.0 and C22.9 excluding other subtypes of liver cancer (i.e., intrahepatic bile duct carcinoma, hepatoblastoma). Sensitivity analysis was conducted using different definitions of HCC (ICD-10 code: C22.0 or C22). CLD included advanced liver disease and liver-related mortality, involving liver cirrhosis, NAFLD, and liver fibrosis (ICD-10 code: K70, K72, and K74 alongside other complications of CLD, Additional file 1: Method S4). We selected these ICD-10 codes to define CLD for the following reasons: (1) these are the standard definitions used in large-scale population-based studies including the UK Biobank and CKB and in well-established risk prediction models for CLD [22–24]; (2) several previous models included in the systematic review did not report the detailed ICD-10 codes so standard definitions need to be applied to improve the generalizability of these models. Based on the prediction time frame, models were further classified into five time intervals: < 5 years, 5 years, 5–10 years, 10 years, and > 10 years.

### Statistical analysis

Ten articles with 13 models were included for external validation in CKB, and all were developed using the Cox proportional hazards model. Because 10 of the 13 models did not report enough information, we fitted Cox regression using predictors as defined in the original studies and updated the model by re-estimating the predictor coefficients in CKB (i.e., “refit”). This approach aimed to evaluate the predictive performance and improve the calibration of the models in the relatively large sample of the CKB, particularly when the provided information was limited [25]. Harrell’s C-index was used to assess discrimination, while the calibration plot and O:E ratio were used to assess calibration. Formulas for each model can be found in Additional file 1: Method S5.

For prediction models that were examined in ≥ 2 independent datasets, we did a random-effects meta-analysis to calculate a summary estimate for model performance and calibration [26]. Discrimination was assessed by comparing discriminative ability using the C-index and AUC [27]. Of the 34 studies included in the meta-analysis, 26 reported AUC, 5 reported C-index, and 3 studies reported both. Therefore, we included the parameter as reported in the original studies and referred to it as “C-index” in the meta-analysis. Where a study reported both parameters, we included C-index in the meta-analysis. Effect sizes and their 95% confidence intervals were combined for the same model across studies to obtain pooled effect estimates using the “metamisc” package [26]. All statistical analyses were performed using R version 4.2.1.

## Results

### Characteristics of the included models

**Fig. 1:**
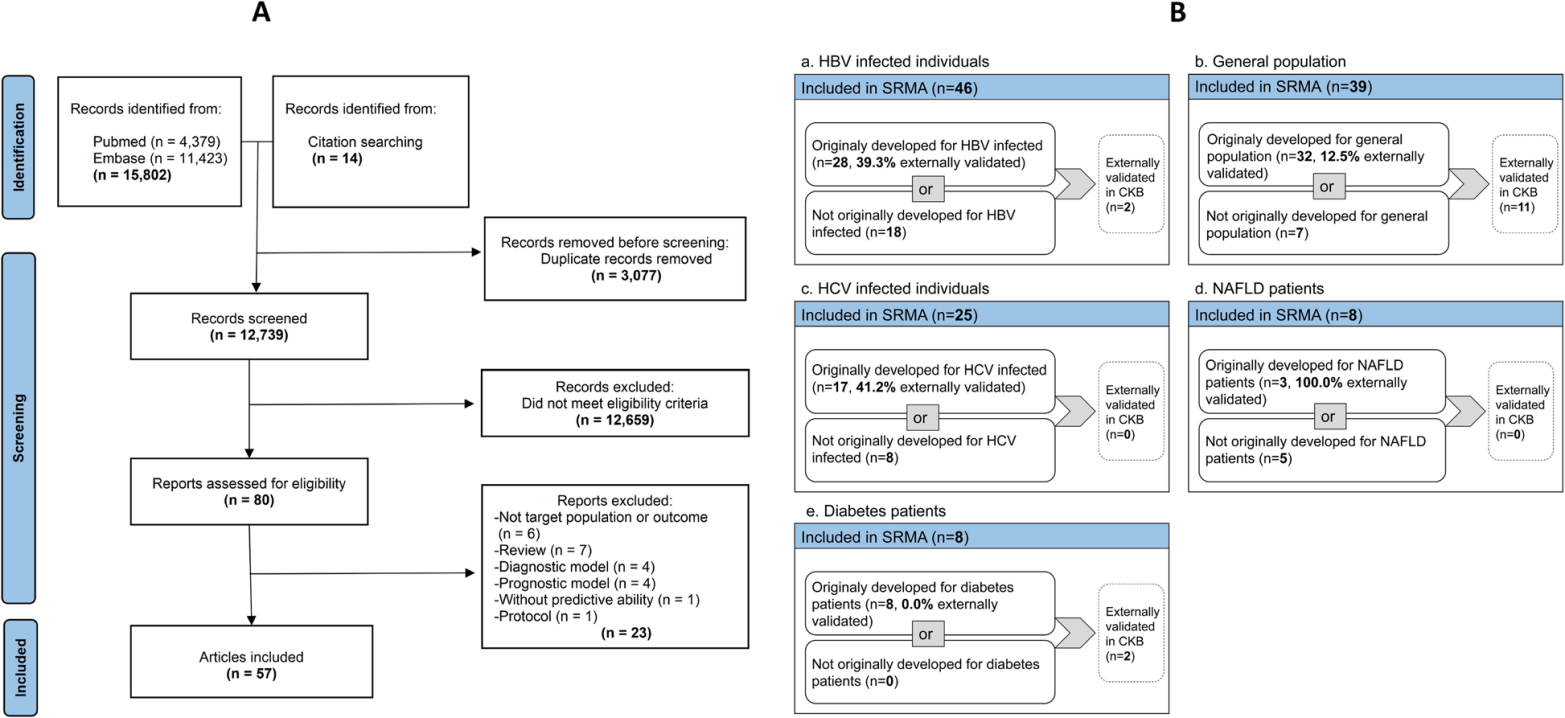
Flow chart. **A** Flow chart for screening eligible publications. **B** Number of models included in the systematic review and external validation of CKB. The larger box corresponds to the aggregate number of models included in the systematic review, including (1) models originally developed for the target population and (2) those previously validated within the target population (not originally developed for the target populations). The smaller box represents models externally validated in CKB, including 11 models validated in the general population and 2 models validated in HBV infected individuals. Abbreviations: CKB, China Kadoorie Biobank; HBV, hepatitis B virus; HCV, hepatitis C virus; NAFLD, non-alcoholic fatty liver disease; SRMA, systematic review and meta-analysis

A total of 12,725 articles were initially screened and 80 relevant articles were included for full-text screening, of which 57 articles were eligible for inclusion (Fig. 1A) [22, 28–83]. The systematic review identified 114 models (39 for general, 46 for HBV, 25 for HCV, 8 for NAFLD, and 8 for diabetes, including 7 models for ≥ 2 populations) (see Additional file 2). After excluding models that were only externally validated but not originally developed for the study population (e.g., BARD initially for NAFLD, later validated in the general population), there were 32, 28, 17, 3, and 8 models developed specifically for general population, HBV infected individuals, HCV infected individuals, NAFLD patients, and diabetes patients, respectively. Among these models, 12.5% (4/32), 39.3% (11/28), 41.2% (7/17), 100.0% (3/3), and 0% (0/8), had been externally validated in the respective populations (Fig. 1B). Overall, only 28.4% of the models underwent external validation. Although there were also prediction models in alcoholic fatty liver (AFLD) patients, ALD patients, drinkers, and obese individuals, the number of models specifically developed in these sub-populations was limited. In terms of time horizon and outcomes, the commonest combinations were 5-year and 10-year HCC. Detailed information on study information and bias assessment was reported in Additional file 1: Table S1.

Three categories of predictors were considered: non-laboratory parameters, HBV/HCV laboratory parameters, and non-HBV/HCV laboratory parameters. Additional file 1: Tables S2–4 show the number of models for these categories in the general population, HBV infected individuals, and other populations. The commonest predictors were age and gender. In prediction models developed for the general population, frequently used predictors also included (descending order by frequency): diabetes (17/32), alcohol (15/32), smoking (13/32), physical activity (12/32), alanine aminotransferase (ALT) (15/32), aspartate aminotransferase (AST) (11/32), and gamma-glutamyl transferase (GGT) (9/32). In contrast, virological parameters were common for HBV infected individuals (HBV DNA 16/28 and hepatitis B e antigen [HBeAg] 14/28).

### CKB external validation

Due to the availability of predictors in the CKB study, 10 articles with 13 models were included for external validation, involving the general population, HBV infected individuals, and type 2 diabetes (T2D) patients. Among the 13 models for external validation, 3 models were based on non-lab predictors (Wen 1–2012, HLI, and CLivD score (non-lab)), while the other 10 models included blood-based biomarkers (Table 1).

**Table 1:** Predictors in 13 CLD risk prediction models validated in CKB

Model	Demographic		Lifestyle						Personal and family history			Blood-based biomarkers			
	Age	Sex	Alcohol	BMI	Diet	PA	Smoking	WHR	Cirrhosis	Diabetes	Hepatitis	ALT	AST	GGT	TG
BARD				**•**						**•**		**•**	**•**		
Cao-2021^a^	**•**	**•**	**•**	**•**					**•**^b^						
CAP-B^c^	**•**	**•**	**•**				**•**			**•**	**•**^d^	**•**		**•**	
CLivD (lab)	**•**	**•**	**•**				**•**	**•**		**•**				**•**	
CLivD (non-lab)	**•**	**•**	**•**				**•**	**•**		**•**					
dAAR	**•**											**•**	**•**		
DM-HCC	**•**													**•**	**•**
HLI			**•**	**•**	**•**	**•**	**•**			**•**	**•**				
Li-2018^e^	**•**	**•**					**•**		**•**		**•**	**•**			
Sinn-2020	**•**	**•**					**•**			**•**		**•**			**•**
Wen 1-2012	**•**	**•**	**•**			**•**	**•**			**•**					
Wen 2-2012	**•**	**•**										**•**	**•**		
Wen 3-2012	**•**	**•**	**•**			**•**	**•**			**•**		**•**	**•**		

*Abbreviations*: *ALT *Alanine aminotransferase, *AST *Aspartate aminotransferase, *BMI *Body mass index, *GGT *Gamma-glutamyl transferase, *HBV *Hepatitis B virus, *HCV *Hepatitis C virus, *PA *Physical activity, *TG *Triglycerides, *WHR *Waist-to-hip ratio

^a^Psychological trauma was included as an additional predictor in Cao-2021

^b^Cirrhosis was defined as medical history of liver diseases in mothers

^c^Additional predictors included income, statin exposure, and antiplatelet

^d^Hepatitis was defined as hepatitis C

^e^Additional predictors included HbA1c, antidiabetes medication, antihyperlipidemia medication, and THR, which was included as an additional predictor in Li-2018

Of the 8 models for HCC, 5 models were developed for the general populations, 2 models for diabetes patients (DM-HCC and Li-2018), and 1 for HBV infected individuals (Cao-2021) (Table 2). DM-HCC and Li-2018 were also validated in the general population in CKB. Among the general population, only the HLI model showed higher discrimination in CKB than in the original development study (0.68 vs 0.64). The Wen 1–2012 and Li-2018 models exhibited favorable discrimination with C-index ≥ 0.70 for both 5-year and 10-year risk. Among patients with T2D, both the DM-HCC and Li-2018 models exhibited high discrimination, but this was probably because of the small number of cases. Among HBV infected individuals, the Cao-2021 model had a C-index of 0.73 for 10-year prediction. Sensitivity analyses with different definitions of HCC (ICD-10: C22.0 and C22) showed similar results (Additional file 1: Table S5).

**Table 2:** Discrimination of 10-year CLD model in the published literature and CKB

Model	Population	Development cohort			Published validation cohort		CKB	
		Area	Events/total	C-index (95% CI)	Events/total	C-index (95% CI)	Events/total	C-index (95% CI)
HCC								
DM-HCC (all)^a^	General	EAS	–	–	–	–	26/15,818	0.66 (0.59–0.73)
HLI	General	EUR	712/477,206	0.64 (0.57–0.70)	–	–	1709/478,930	0.68 (0.67–0.70)
Li-2018 (all)^a^	General	EAS	–	–	–	–	72/17,227	0.74 (0.68–0.80)
Sinn-2020	General	EAS	236/467,206	0.83 (0.77–0.88)	35/91,357	0.92 (0.89–0.95)	72/17,227	0.66 (0.60–0.72)
Wen 1-2012	General	EAS	1252/298,051	0.81 (0.80–0.83)	–	–	1709/478,930	0.72 (0.70–0.73)
Wen 2-2012	General	EAS	1252/298,051	0.90 (0.90–0.92)	–	–	72/17,227	0.67 (0.61–0.74)
Wen 3-2012	General	EAS	1252/298,051	0.91 (0.89–0.93)	–	–	72/17,227	0.68 (0.61–0.74)
Cao-2021	HBV	EAS	–	–	–	–	532/13,723	0.73 (0.71–0.75)
DM-HCC	T2D	EAS	36/2364^b^	0.86 (0.85–0.88)^b^	–	–	6/1348	0.78 (0.53–0.99)
Li-2018	T2D	EAS	493/21,149	0.77 (0.75–0.79)	–	–	7/1540	0.96 (0.93–0.99)
CLD								
BARD^c^	General	EUR	–	–	232/75,303	0.53 (0.50–0.57)	150/17,227	0.55 (0.52–0.58)
CLivD score (lab)	General	EUR	273/25,760^d^	0.84 (0.75–0.93)^d^	64/3049^d^	0.78 (0.71–0.83)^d^	141/15,945	0.74 (0.70–0.78)
CLivD score (non-lab)	General	EUR	273/25,760^d^	0.82 (0.74–0.91)^d^	118/8107^d^	0.70 (0.14–0.97)^d^	3207/478,930	0.71 (0.70–0.72)
dAAR	General	EUR	89/18,067^e^	0.80 (0.74–0.85)^e^	717/126,941	0.72 (0.70–0.74)	150/17,227	0.72 (0.68–0.76)
CAP-B	HBV	EAS	16,492/401,745	0.78 (0.78–0.78)	–	–	28/394	0.77 (0.68–0.86)

*Abbreviations*: *CKB *China Kadoorie Biobank, *EAS *East Asia, *EUR *Europe, *HCC*, Hepatocellular carcinoma, *CLD *Chronic liver disease

^a^Due to the small number of HCC cases among diabetes patients in CKB, the predictive ability of the DM-HCC and Li-2018 models was also evaluated in the general population

^b^The values reported are based on the 5-year HCC outcome, given the absence of the 10-year HCC outcome in the original development cohort

^c^The model was first developed for NAFLD patients in the US (lacking specific development data), but was later validated in the general population

^d^The values reported are based on 15-year risk of CLD, given the absence of the 10-year CLD outcome in the development or external validation cohort

^e^The values reported are based on 8.2-year risk of CLD, given the absence of the 5- or 10-year CLD outcome in the original development cohort

Of the 5 models for CLD, 4 models were developed for the general population and 1 for HBV infected individuals. In the general population, all models showed lower discrimination compared with the development cohort. BARD and CLivD score (non-lab) for 10-year prediction slightly better discrimination compared with previous external validation studies. Among all models, CLivD score (non-lab and lab) and dAAR showed C-index higher than 0.70 for 10-year CLD. For HBV infected individuals, the CAP-B model showed a C-index of 0.77 for predicting 10-year CLD (Table 2). Similar model performance was shown for 5-year HCC and 5-year CLD prediction (Additional file 1: Tables S6–7).

**Fig. 2:**
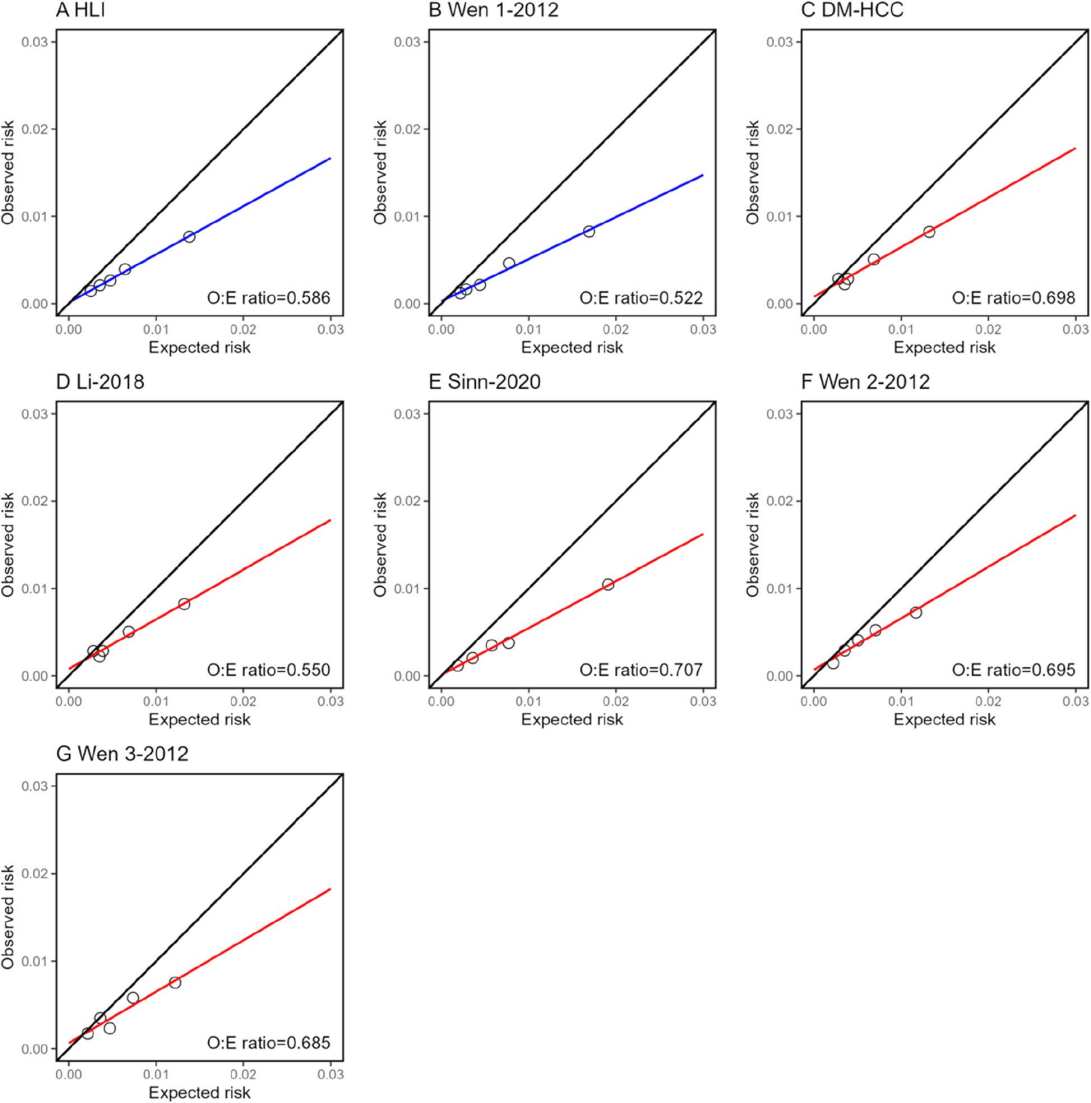
Calibration plots of 10-year HCC risk prediction models in CKB. Non-lab models and lab models are shown using different colors (blue for non-lab models and red for lab models). Observed to expected (O:E) ratio are shown in lower-right corner of each panel

**Fig. 3:**
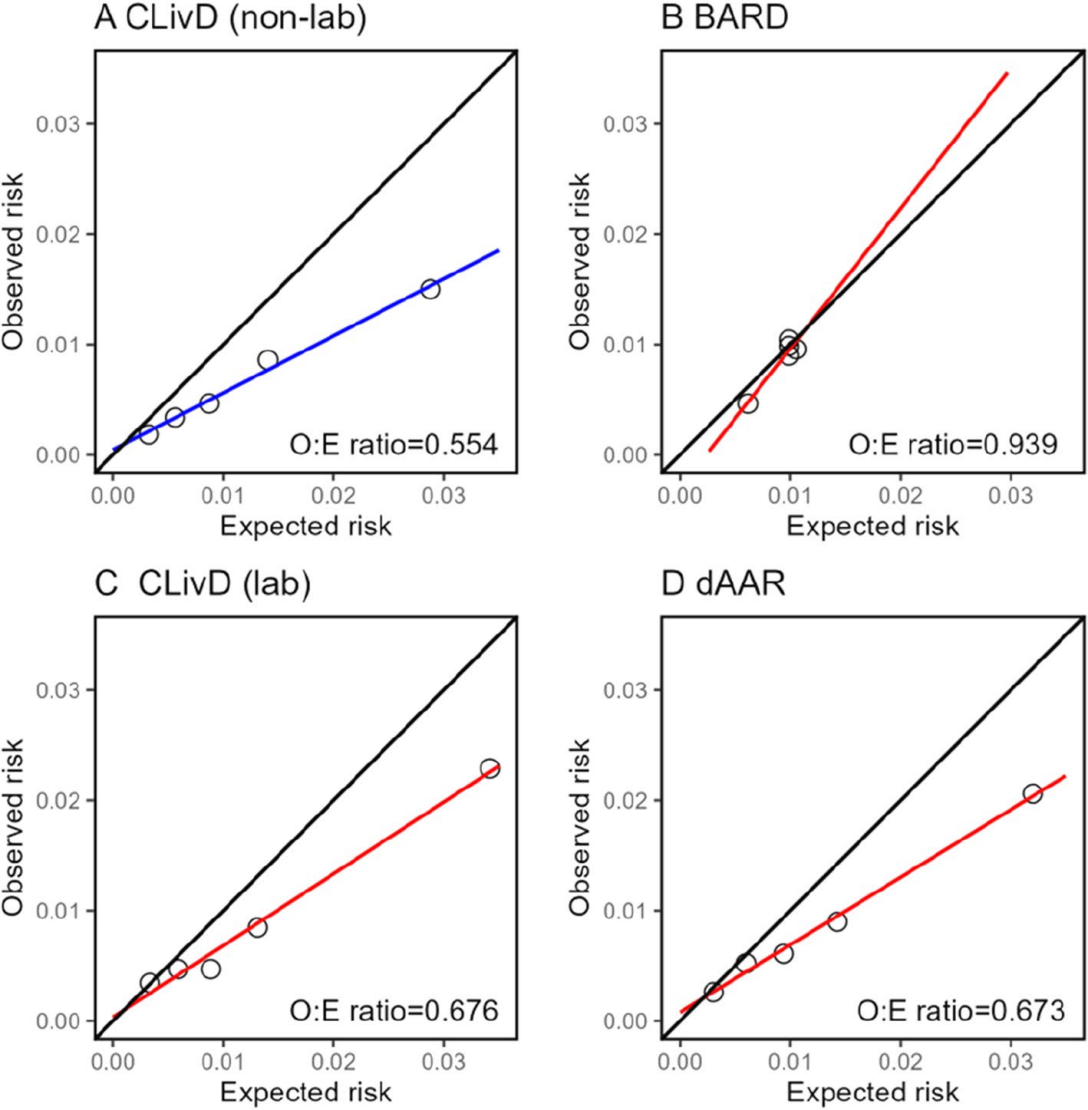
Calibration plots of 10-year CLD risk prediction models in CKB. Convention as in Fig. 2

Model calibration in CKB was shown for HCC and CLD, separately. For 10-year risk of HCC, calibration across all models showed overestimation (Fig. 2). Similar patterns were observed for 5-year calibration (Additional file 1: Fig. S1). For 10-year risk of CLD, the CLivD score (non-lab and lab) and dAAR overestimated the risk, while BARD overestimated the risk at lower levels of observed risk (Fig. 3). The calibration of the BARD and dAAR score was slightly better for 5-year risk of CLD (Additional file 1: Fig. S2).

### Meta-analysis of the model performance

**Fig. 4:**
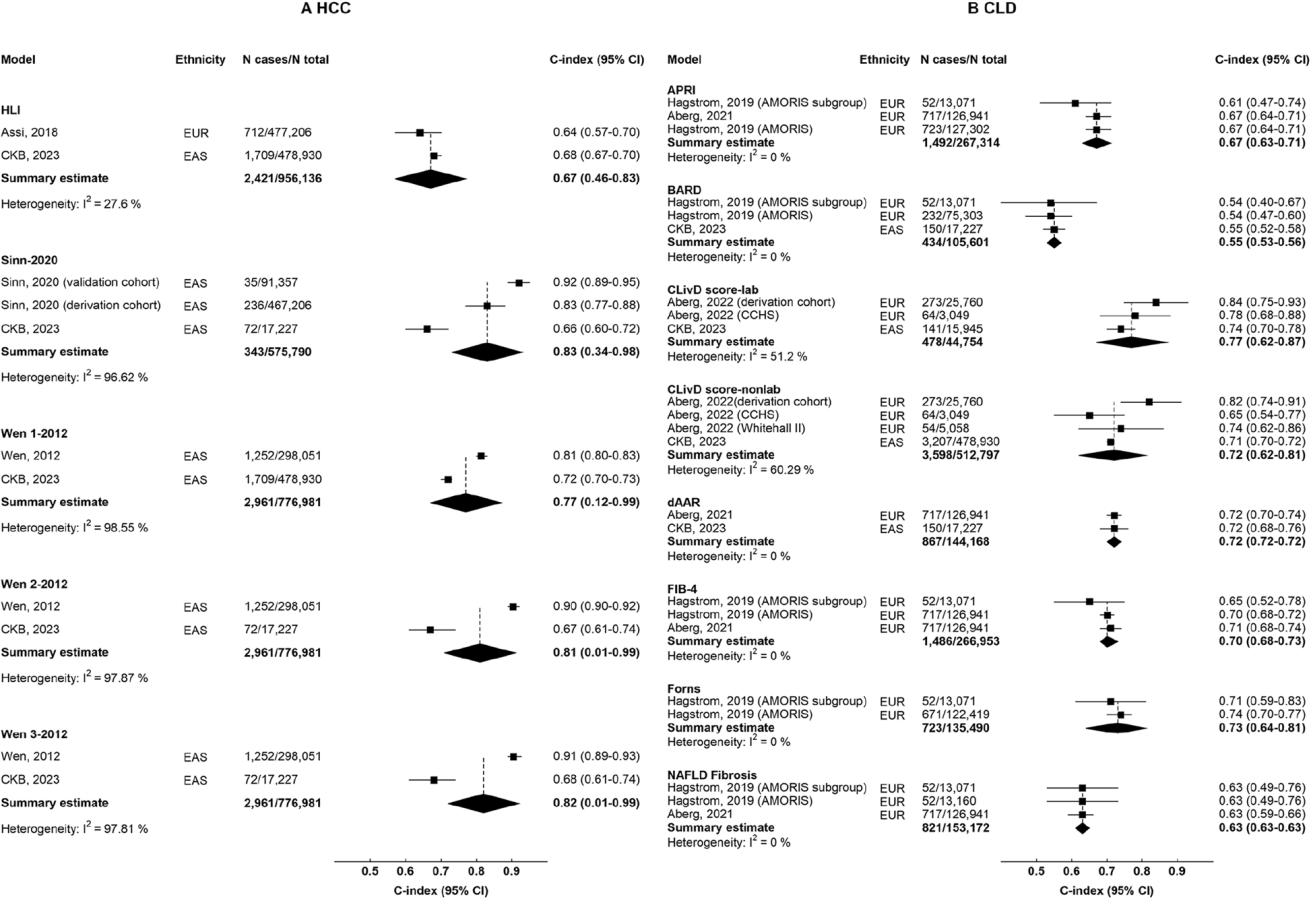
C-index of risk prediction models for HCC and CLD in meta-analysis of CKB and published studies. Boxes represent the C-index for predicting 10-year **A** HCC or **B** CLD in the general population. Diamonds represent summary C-index for each model, with the size of the diamond showing 95% confidence interval. For each model, published studies are sorted according to number of participants. Estimates and 95% CI of the summary C-index are shown in bold. The CIs of the summary estimates for HCC models were truncated because of the relatively low SE calculated using the “metamisc” package

In the general population, there were 5 models for HCC and 8 models for CLD eligible for meta-analysis. For 10-year risk of HCC (Fig. 4A), Sinn-2020, Wen-1 2012, Wen-2 2021, and Wen-3 2012 showed good performance (C-index ≥ 0.70), albeit large heterogeneity between studies. For 10-year risk of CLD (Fig. 4B), CLivD score (lab and non-lab), dAAR, FIB-4, and Forns showed good performance (C-index ≥ 0.70). Model performance for 5-year and > 10-year are shown in Additional file 1: Fig. S3. Detailed results for all models are reported in Additional file 1: Fig. S4.

In HBV infected individuals, there were 13 models for HCC and 2 models for CLD eligible for meta-analysis. The majority of models had C-index above 0.70 in previous external validation. The RWS-HCC, GAG-HCC, and REACH-B IIa models exhibited C-index ≥ 0.80 for 10-year HCC prediction, followed by mREACH-B model (C-index: 0.79–0.83 for < 10-year HCC prediction). FIB-4, Ishak fibrosis, PAGE-B, and PAGE-B + Ishak showed favorable discrimination (C-index ≥ 0.85), but they were only externally validated in one study (Additional file 1: Fig. S5). GAG-HCC, PAGE-B, and PAGE-B + Ishak also showed good discrimination (C-index ≥ 0.80) for predicting 10-year risk of CLD (Additional file 1: Fig. S6).

In HCV infected individuals, there were 5 models for HCC and 3 models for CLD eligible for meta-analysis, with FIB-4 showing the highest discrimination for HCC and CLD across all time frames (C-index 0.76–0.85 for < 5, 5, 5–10 years) (Additional file 1: Fig. S7). In NAFLD patients, there were 3 models for HCC and 1 model for CLD eligible for meta-analysis. LS-Based Model 2 and dAAR showed the highest discrimination for HCC and CLD (C-index 0.78 and 0.84), respectively (Additional file 1: Fig. S8).

For calibration, there was considerable variability in reporting across different studies, including (1) calibration plots (*n* = 13); (2) correlation coefficients between observed and predicted risks (*n* = 2); (3) the O:E ratio (*n* = 1, Kurosaki-2012 [32]); and (4) Brier scores (*n* = 1, An-2021 [70]). The majority of models (78/92) did not report calibration in external validation, and meta-analysis was therefore not feasible.

## Discussion

We conducted a systematic review of CLD prediction models developed for different populations, meta-analyzed their performance, and independently validated selected models in the CKB cohort. Our findings showed substantial variation in model predictors and predictive performance. Lifestyle factors were the commonest predictors for the general population, while virological and biochemical markers were the commonest predictors for individuals with HBV, HCV, and NAFLD. A total of 13 models were included for external validation in the CKB cohort, showing distinct differences in discrimination, with C-index ranging from 0.55 to 0.96. Of all 11 models validated in the general population, Li-2018 and Wen 1–2012 had good discrimination for 10-year HCC, while CLivD score (non-lab and lab) and dAAR had the highest discrimination for 10-year CLD in CKB.

We summarized the best-performing models for 10-year risk of CLD by study populations according to the results of the meta-analysis and external validation in CKB. For the general population, Li-2018, Wen 1–2012, Wen 2–2012, Wen 3–2012, and Sinn-2020 models showed good discrimination for HCC, while CLivD score (non-lab and lab), dAAR, FIB-4, and Forns showed good discrimination for CLD. Additionally, our meta-analysis showed good discrimination of the Forns score for CLD, with higher C-index than other non-invasive scores for fibrosis (i.e., BARD, FIB-4, and APRI); however, validation in CKB was hindered by the lack of timely on-site testing of platelet count in large-scale cohort studies [84]. For individuals with diabetes, both the Li-2018 model and DM-HCC model showed good performance for HCC in the original development and CKB; however, the high C-index may be due to the limited number of CLD cases among diabetes patients in CKB. For HBV infected individuals, our meta-analysis highlighted the favorable performance of the RWS-HCC, REACH-B IIa, and GAG-HCC models. Only Cao-2021 and CAP-B models were externally validated in CKB, both showing good discrimination for CLD. For HCV infected individuals, NAFLD patients, and high-risk populations, there was a lack of adequate studies for model development and validation. Specifically, prediction models for HCV infected individuals lacked long-term prediction (> 10 years). FIB-4 showed the best discrimination for short- to medium-term risks of HCC and CLD. Prediction models for NAFLD patients relied heavily on transient elastography technology and had limited generalizability.

Although 9 out of 13 models showed slightly lower predictive performance in the CKB cohort compared to their previous development cohorts, our study findings supported the transportability of CLD models in Chinese adults by showing generally good performance. Indeed, the discrepancy in model performance between CKB and previous studies may be attributed to differences in the etiology of CLD and risk factor profiles, particularly between East Asians and Caucasians. The GBD 2019 study showed that the leading causes of CLD prevalence were NAFLD (82%) and hepatitis B (9%) in high Socio-Demographic Index (SDI) countries (primarily European and North American countries) and NAFLD (69%) and hepatitis B (26%) in China (Additional file 1: Table S8). Although previous studies in CKB reported lifestyle and metabolic risk factors for CLD similar to those in Western populations (Additional file 1: Table S9) [24, 85–88], the magnitude of associations differed for several risk factors (e.g., adiposity and physical activity). Although the predictive performance in CKB was relatively lower, we showed the transportability of risk prediction models developed in Western populations to the Chinese population. Of note, this favorable performance of CLD models was comparable to risk prediction models for cardiovascular disease (CVD) [89] and colorectal cancer (CRC) [90] in CKB. The WHO risk chart with non-laboratory data to predict 10-year CVD risk achieved a C-statistic of 0.75, where well-established models for CRC involving non-laboratory parameters had C-statistics between 0.65 and 0.70. Despite the different etiologies of CLD and magnitude of associations between primary risk factors and CLD, HLI, Li-2018, and BARD models performed better in the CKB cohort compared to its original development or validation cohorts. Risk prediction models incorporating combined risk factors for CLD, analogous to the Framingham risk score or PCE score used for CVD, would be promising to risk-stratify individuals before clinical onset of advanced liver disease.

Our study findings may have public health implications. Currently, EASL and AASLD recommend ultrasound screening for liver cancer among individuals diagnosed with cirrhosis, CHB, or CHC. In China, the screening criteria also encompass individuals with a family history of liver cancer, and serum AFP is used as an additional screening measure (Additional file 1: Table S10) [5–7]. However, these screening strategies are limited by the high number needed to screen and the reliance on secondary or tertiary healthcare centers. The international community of hepatologists has recommended screening for high-risk individuals by non-invasive tests that are widely available in primary healthcare settings, followed by second-line confirmatory tests (e.g., abdominal ultrasound). In this context, we showed feasibility of combining non-laboratory parameters and routinely measured liver biomarkers in risk stratification of CLD. Two models (Wen 1–2012 and CLivD score (non-lab)) were based on non-laboratory parameters and achieved C-index ≥ 0.70 in the general Chinese population, while CLivD score (lab) incorporated non-laboratory parameters plus GGT also had good performance. Such predictive models can enable early case-finding and individualized follow-up for adults at risk of liver disease in primary care and non-liver healthcare settings. They may also help prevent disease progression by facilitating timely interventions, such as weight loss or alcohol rehabilitation, thereby reducing the risk of severe liver conditions and associated mortality. Furthermore, these models provide valuable data on CLD risk to local policymakers and health authorities, helping the development of public health strategies.

Study limitations included uncertainty in risk estimates when dealing with relatively rare outcomes, especially among patients with diabetes. This may lead to overly optimistic estimation of discrimination. In addition, the eligibility criteria varied across the original development cohorts. Of note, the Sinn-2020 model for 10-year HCC prediction showed the highest discrimination in the meta-analysis but moderate performance in the CKB cohort. This discrepancy could be attributed to the fact that the Sinn-2020 model originated from a health screening cohort where volunteer selection bias might exist, potentially limiting the generalizability. Moreover, only 3 out of 13 models provided complete information to assess model performance, including regression coefficients and baseline survival rates. For this reason, a refitting approach was employed to evaluate calibration, which might lead to underestimation or overestimation because of the inherent limitations or biases in the study design and predictors of the original model. Lastly, the CKB involved 5 urban and 5 rural areas in China and was not nationally representative. However, the large sample size, the diversity of regions covered, the heterogeneity in exposures, and consistent findings from subgroup analyses suggest that our model’s performance results are largely generalizable to the broader Chinese population.

## Conclusions

In conclusion, our meta-analysis and external validation in Chinese adults showed that several models had good discrimination and calibration with potential to identify high-risk populations for CLD, who would be referred to liver clinics for further assessment and be the target population of lifestyle modifications. Future studies are warranted to validate the performance of CLD prediction models in diverse populations and to assess the cost-effectiveness of screening strategies for CLD.

## Supplementary Information

Additional file 1: Method S1 Search strategy. Method S2 China Kadoorie Biobank (CKB) information. Method S3 The measurement and definition of CKB predictor variables. Method S4 ICD-10 of CLD events in CKB. Method S5 Predictors and equations for included models. Table S1 Bias assessment. Table S2 Predictors of prediction models for general population. Table S3 Predictors of prediction models for HBV infected individuals. Table S4 Predictors of prediction models for other populations. Table S5 Sensitivity analysis for PLC and HCC. Table S6 CLD risk model for 5-year HCC discrimination in the published literature and CKB. Table S7 CLD risk model for 5-year CLD discrimination in the published literature and CKB. Table S8 CLD diseases and causes in high-income countries and China in 2019. Table S9 Risk factors (HR (95% CI)) for CLD in CKB and Western populations. Table S10 Summary of liver cancer screening strategies. Fig. S1 Calibration plots of 5-year HCC risk prediction models in the CKB. Fig. S2 Calibration plots of 5-year CLD risk prediction models in the CKB. Fig. S3 Discrimination of CLD risk prediction models in the published literature and CKB. Fig. S4 Discrimination of HCC risk prediction models in the general population. Fig. S5 Discrimination of HCC risk prediction models in the HBV infected individuals. Fig. S6 Discrimination of CLD risk prediction models in the HBV infected individuals. Fig. S7 Discrimination of CLD risk prediction models in the HCV infected individuals. Fig. S8 Discrimination of CLD risk prediction models in the NAFLD patients.

Additional file 2.

## Data Availability

The materials are available upon request; some restrictions will apply.

## Abbreviations

AASLD: American Association for the Study of Liver Diseases
AFLD: Alcoholic fatty liver
AFP: Alpha-fetoprotein
ALD: Alcoholic liver disease
ALT: Alanine aminotransferase
Anti-HCV Ab: Anti-hepatitis C virus antibody
AST: Aspartate aminotransferase
AUC: Area under the ROC curve
BMI: Body mass index
CHB: Chronic hepatitis B
CHC: Chronic hepatitis C
CKB: China Kadoorie Biobank
CLD: Chronic liver disease
CRC: Colorectal cancer
CVD: Cardiovascular disease
EAS: East Asia
EASL: European Association for the Study of the Liver
EUR: Europe
GBD: Global Burden of Disease Study
GGT: Gamma-glutamyl transferase
HBsAg: Hepatitis B surface antigen
HBeAg: Hepatitis B e antigen
HBV: Hepatitis B virus
HCV: Hepatitis C virus
HCC: Hepatocellular carcinoma
ICD: International Classification of Diseases
NAFLD: Non-alcoholic fatty liver disease
PA: Physical activity
PRISMA: Preferred Reporting Items for Systematic Reviews and Meta-Analyses
PROBAST: Prediction model Risk Of Bias Assessment Tool
ROC: Receiver operating characteristic
SDI: Socio-Demographic Index
SLD: Severe liver disease
SRMA: Systematic review and meta-analysis
T2D: Type 2 diabetes
TG: Triglycerides
TRIPOD: Transparent Reporting of a Multivariable Prediction Model for Individual Prognosis or Diagnosis
WHR: Waist-to-hip ratio
