# Supplementary material for: Comparison of models to predict incident chronic liver disease: a systematic review and external validation in Chinese adults
Source: BMC Med. 2024 Dec 31;22:601. doi: 10.1186/s12916-024-03754-9 (PMC11686935; doi:10.1186/s12916-024-03754-9)
Supplement: Supplementary file 1 — Additional file 1: Method S1 Search strategy. Method S2 China Kadoorie Biobank (CKB) information. Method S3 The measurement and definition of CKB predictor variables. Method S4 ICD-10 of CLD events in CKB. Method S5 Predictors and equations for included models. Table S1 Bias assessment. Table S2 Predictors of prediction models for general population. Table S3 Predictors of prediction models for HBV infected individuals. Table S4 Predictors of prediction models for other populations. Table S5 Sensitivity analysis for PLC and HCC. Table S6 CLD risk model for 5-year HCC discrimination in the published literature and CKB. Table S7 CLD risk model for 5-year CLD discrimination in the published literature and CKB. Table S8 CLD diseases and causes in high-income countries and China in 2019. Table S9 Risk factors (HR (95% CI)) for CLD in CKB and Western populations. Table S10 Summary of liver cancer screening strategies. Fig. S1 Calibration plots of 5-year HCC risk prediction models in the CKB. Fig. S2 Calibration plots of 5-year CLD risk prediction models in the CKB. Fig. S3 Discrimination of CLD risk prediction models in the published literature and CKB. Fig. S4 Discrimination of HCC risk prediction models in the general population. Fig. S5 Discrimination of HCC risk prediction models in the HBV infected individuals. Fig. S6 Discrimination of CLD risk prediction models in the HBV infected individuals. Fig. S7 Discrimination of CLD risk prediction models in the HCV infected individuals. Fig. S8 Discrimination of CLD risk prediction models in the NAFLD patients. [file 12916_2024_3754_MOESM1_ESM.pdf]

Supplementary Material

Comparison of models to predict incident chronic liver disease: a systematic review and external validation in Chinese adults

BMC Medicine

Xue Cong, BM[1], Shuyao Song, BM[1], Yingtao Li, MS[1], Kaiyang Song, BA[2], Cameron MacLeod, BA[2], Yujie Cheng, BM[1], Jun Lv, PhD[1,3,4], Canqing Yu, PhD[1,3,4], Dianjianyi Sun, PhD[1,3,4], Pei Pei, MSc[1,3], Ling Yang, PhD[5,6], Yiping Chen, DPhil[5,6], Iona Millwood, DPhil[5,6], Shukuan Wu, BS[7], Xiaoming Yang, PhD[5], Rebecca Stevens, MSc[5], Junshi Chen, MD[8], Zhengming Chen, DPhil[5], Liming Li, MD[1,3,4], Christiana Kartsonaki, DPhil[5,6*]#, Yuanjie Pang, DPhil[1,4*]# on behalf of the China Kadoorie Biobank Collaborative Group**

*Corresponding author (yuanjie.p@gmail.com, christiana.kartsonaki@dph.ox.ac.uk)
#Equal contributors
**The members of steering committee and collaborative group are listed in Supplementary material.

1. Department of Epidemiology & Biostatistics, School of Public Health, Peking University, Beijing 100191, China
2. Medical Sciences Division, University of Oxford, Oxford OX3 9DU, United Kingdom
3. Peking University Center for Public Health and Epidemic Preparedness & Response, Beijing 100191, China
4. Key Laboratory of Epidemiology of Major Diseases (Peking University), Ministry of Education
5. Clinical Trial Service Unit & Epidemiological Studies Unit (CTSU), Nuffield Department of Population Health, University of Oxford
6. Medical Research Council Population Health Research Unit at the University of Oxford, Oxford OX3 7LF, United Kingdom
7. Meilan Center for Disease Control and Prevention, Haikou 570100, China
8. China National Center for Food Safety Risk Assessment, Beijing 100022, China

Address for correspondence:

Dr. Yuanjie Pang
Department of Epidemiology & Biostatistics
School of Public Health
Peking University
38 Xueyuan Road
Beijing, 100191, China
Tel: 86-010-82801528
Fax: 86-010-82801530
yuanjie.p@gmail.com

Dr. Christiana Kartsonaki
Nuffield Department of Population Health
University of Oxford
Old Road Campus
Oxford, OX3 7LF, UK
Tel: 86-010-82801528
Fax: 86-010-82801530
christiana.kartsonaki@dph.ox.ac.uk

Table of contents

Members of the China Kadoorie Biobank collaborative group 3
Method S1 Search strategy 4
Method S2 China Kadoorie Biobank (CKB) information 6
Method S3 The measurement and definition of CKB predictor variables 8
Method S4 ICD-10 of CLD events in CKB 11
Method S5 Predictors and equations for included models 12
Table S1 Bias assessment 20
Table S2 Predictors of prediction models for general population 22
Table S3 Predictors of prediction models for HBV infected individuals 24
Table S4 Predictors of prediction models for other populations 26
Table S5 Sensitivity analysis for PLC and HCC 28
Table S6 CLD risk model for 5-year HCC discrimination in the published literature and CKB 29
Table S7 CLD risk model for 5-year CLD discrimination in the published literature and CKB 30
Table S8 CLD diseases and causes in high income countries and China in 2019 31
Table S9 Risk factors for CLD in CKB and Western populations 32
Table S10 Summary of Liver Cancer Screening Strategies 33
Fig. S1 Calibration plots of 5-year HCC risk prediction models in the CKB 35
Fig. S2 Calibration plots of 5-year CLD risk prediction models in the CKB 36
Fig. S3 Discrimination of CLD risk prediction models in the published literature and CKB 37
Fig. S4 Discrimination of HCC risk prediction models in the general population 38
Fig. S5 Discrimination of HCC risk prediction models in the HBV infected individuals 39
Fig. S6 Discrimination of CLD risk prediction models in the HBV infected individuals 40
Fig. S7 Discrimination of CLD risk prediction models in the HCV infected individuals 41
Fig. S8 Discrimination of CLD risk prediction models in the NAFLD patients 42

Members of the China Kadoorie Biobank collaborative group

International Steering Committee: Junshi Chen, Zhengming Chen (PI), Robert Clarke, Rory Collins, Yu Guo, Liming Li (PI), Jun Lv, Richard Peto, Robin Walters. **International Co-ordinating Centre, Oxford:** Daniel Avery, Derrick Bennett, Ruth Boxall, Sue Burgess, Ka Hung Chan, Yumei Chang, Yiping Chen, Zhengming Chen, Johnathan Clarke, Robert Clarke, Huaidong Du, Ahmed Edris Mohamed, Zammy Fairhurst-Hunter, Hannah Fry, Simon Gilbert, Alex Hacker, Mike Hill, Michael Holmes, Pek Kei Im, Andri Iona, Maria Kakkoura, Christiana Kartsonaki, Rene Kerosi, Kuang Lin, Mohsen Mazidi, Iona Millwood, Sam Morris, Qunhua Nie, Alfred Pozarickij, Paul Ryder, Saredo Said, Sam Sansome, Dan Schmidt, Paul Sherliker, Rajani Sohoni, Becky Stevens, Iain Turnbull, Robin Walters, Lin Wang, Neil Wright, Ling Yang, Xiaoming Yang, Pang Yao.

National Co-ordinating Centre, Beijing: Yu Guo, Xiao Han, Can Hou, Jun Lv, Pei Pei, Chao Liu, Canqing Yu, Qingmei Xia. **10 Regional Co-ordinating Centres: Qingdao CDC:** Zengchang Pang, Ruqin Gao, Shanpeng Li, Haiping Duan, Shaojie Wang, Yongmei Liu, Ranran Du, Yajing Zang, Liang Cheng, Xiaocao Tian, Hua Zhang, Yaoming Zhai, Feng Ning, Xiaohui Sun, Feifei Li. **Licang CDC:** Silu Lv, Junzheng Wang, Wei Hou. **Heilongjiang Provincial CDC:** Wei Sun, Shichun Yan, Xiaoming Cui. **Nangang CDC:** Chi Wang, Zhenyuan Wu,Yanjie Li, Quan Kang. **Hainan Provincial CDC:** Huiming Luo, Tingting Ou. **Meilan CDC:** Xiangyang Zheng, Zhendong Guo, Shukuan Wu, Yilei Li, Huimei Li. **Jiangsu Provincial CDC:** Ming Wu, Yonglin Zhou, Jinyi Zhou, Ran Tao, Jie Yang, Jian Su. **Suzhou CDC:** Fang Liu, Jun Zhang, Yihe Hu, Yan Lu, Liangcai Ma, Aiyu Tang, Shuo Zhang, Jianrong Jin, Jingchao Liu. **Guangxi Provincial CDC:** Mei Lin, Zhenzhen Lu. **Liuzhou CDC:** Lifang Zhou, Changping Xie, Jian Lan, Tingping Zhu, Yun Liu, Liuping Wei, Liyuan Zhou, Ningyu Chen, Yulu Qin, Sisi Wang. **Sichuan Provincial CDC:** Xianping Wu, Ningmei Zhang, Xiaofang Chen, Xiaoyu Chang. **Pengzhou CDC:** Mingqiang Yuan, Xia Wu, Xiaofang Chen, Wei Jiang, Jiaqiu Liu, Qiang Sun. **Gansu Provincial CDC:** Faqing Chen, Xiaolan Ren, Caixia Dong. **Maiji CDC:** Hui Zhang, Enke Mao, Xiaoping Wang, Tao Wang, Xi zhang. **Henan Provincial CDC:** Kai Kang, Shixian Feng, Huizi Tian, Lei Fan. **Huixian CDC:** XiaoLin Li, Huarong Sun, Pan He, Xukui Zhang. **Zhejiang Provincial CDC:** Min Yu, Ruying Hu, Hao Wang. **Tongxiang CDC**: Xiaoyi Zhang, Yuan Cao, Kaixu Xie, Lingli Chen, Dun Shen. **Hunan Provincial CDC:** Xiaojun Li, Donghui Jin, Li Yin, Huilin Liu, Zhongxi Fu. **Liuyang CDC:** Xin Xu, Hao Zhang, Jianwei Chen, Yuan Peng, Libo Zhang, Chan Qu.

Method S1 Search strategy

No.	Entry terms
**Pubmed**	
1	"hepatocellular carcinoma"[Title/Abstract] OR "liver cancer"[Title/Abstract] OR "cirrhosis"[Title/Abstract] OR "chronic liver disease*"[Title/Abstract] OR "severe liver disease*"[Title/Abstract] (n=220,289)
2	"risk assessment"[MeSH Terms] OR "risk prediction"[Title/Abstract] OR "risk score"[Title/Abstract] OR "risk calculation"[Title/Abstract] OR "prediction model"[Title/Abstract] OR "predict index"[Title/Abstract] OR "decision rule"[Title/Abstract] OR "discrimination"[Title/Abstract] OR "roc curve"[Title/Abstract] OR "calibration"[Title/Abstract] OR "AUC"[Title/Abstract] OR "area under the curve"[Title/Abstract] OR "scoring system"[Title/Abstract] OR "outcome prediction"[Title/Abstract] OR "risk classification"[Title/Abstract] OR "forecasting"[Title/Abstract] OR "forecast"[Title/Abstract] OR "decision tree"[Title/Abstract] OR "predictive score"[Title/Abstract] OR "validat*"[Title/Abstract] (n=1,355,701)
3	1 AND 2 Filters: Humans (n=13,548)
4	("prospective study"[Title/Abstract] OR "retrospective study"[Title/Abstract] OR "cohort study"[Title/Abstract] OR "case control study"[Title/Abstract] OR "prospective"[Title/Abstract] OR "retrospective"[Title/Abstract] OR "cohort"[Title/Abstract] OR "case control"[Title/Abstract] OR "case cohort"[Title/Abstract]) AND (humans [Filter]) Filters: Humans (n=1,570,078)
5	("review"[Title] OR "letter"[Publication Type] OR "interview"[Publication Type] OR "comment"[Publication Type] OR "news"[Publication Type] OR "guideline"[Publication Type] OR "editorial"[Publication Type] OR "Bibliography"[Publication Type]) (n=2,995,276)
6	3 AND 4 NOT 5 (n=4,021)
7	3 Filters: meta-analysis Filters: systematic review (n=429)
8	6 OR 7 (n=4,379)
**Embase**	
1	hepatocellular carcinoma.ab. or hepatocellular carcinoma.ti. or liver cancer.ab. or liver cancer.ti. or cirrhosis.ab. or cirrhosis.ti. or chronic liver disease*.ab. or chronic liver disease*.ti. or severe liver disease*.ab. or severe liver disease*.ti. (n=313,023)
2	exp risk assessment/ (n=690,077)
3	risk prediction.ab. or risk prediction.ti. or risk score.ab. or risk score.ti. or risk calculation.ab. or risk calculation.ti. or prediction model.ab. or prediction model.ti. or predict index.ab. or predict index.ti. or decision rule.ab. or decision rule.ti. or discrimination.ab. or discrimination.ti. or roc curve.ab. or roc curve.ti. or calibration.ab. or calibration.ti. or AUC.ab. or AUC.ti. or area under the curve.ab. or area under the curve.ti. or scoring system.ab. or scoring system.ti. or outcome prediction.ab. or outcome prediction.ti. or risk classification.ab. or risk classification.ti. or forecasting.ab. or forecasting.ti. or forecast.ab. or forecast.ti. or decision tree.ab. or decision tree.ti. or predictive score.ab. or predictive score.ti. or

	validat*.ab. or validat*.ti. (n=1,502,514)
4	2 or 3 (n=2,121,759)
5	1 and 4 (n=31,685)
6	limit 5 to human (n=29,515)
7	prospective study.ab. or prospective study.ti. or retrospective study.ab. or retrospective study.ti. or cohort study.ab. or cohort study.ti. or case control study.ab. or case control study.ti. or prospective.ab. or prospective.ti. or retrospective.ab. or retrospective.ti. or cohort.ab. or cohort.ti. or case control.ab. or case control.ti. or case cohort.ab. or case cohort.ti. (n=2,953,555)
8	limit 7 to human (n=2,768,117)
9	review.ti. or letter.pt. or interview.pt. or comment.pt. or news.pt. or guideline.pt. or editorial.pt. or Bibliography.pt. (n=2,689,154)
10	6 and 8 (n=10,777)
11	10 not 9 (n=10,579)
12	limit 6 to meta analysis (n=652)
13	limit 6 to "systematic review" (n=808)
14	12 or 13 (n=1,001)
15	11 or 14 (n=11,427)

Method S2 China Kadoorie Biobank (CKB) information

The external validation population was derived from the China Kadoorie Biobank (CKB), a large prospective Chinese population-based cohort study. The CKB study conducted the baseline survey from June 2004 to July 2008 in five urban and five rural areas of China, recruiting 512,891 adults aged 30 to 79 years[1]. The survey included questionnaire assessments, physical measurements, and collection of blood samples. The questionnaire included sociodemographic characteristics, lifestyle factors, personal and family medical history, and current medication use, etc. Physical measurements included height, weight, hip and waist circumference, blood pressure, and heart rate, etc. See the table below for predictors detail. All survey staff underwent standardized training and implemented computerized data management throughout the project to ensure consistency in survey protocols across regions.

In the CKB validation population, the following individuals were included:

1. General population: The total number of participants was 512,726. Individuals with baseline diagnoses of hepatitis or cirrhosis (n=6,193), cancer (n=2,578), or positive HBsAg results (n=27,285) at the baseline were excluded. The total study cohort included 478,930 individuals. For prediction models incorporating blood-based biomarkers, the sub-population analysis included 15,945 participants with GGT data, and 17,227 participants with ALT and AST data, applying the same exclusion criteria.

2. **HBV Infected individuals:** The total number of participants with positive HBsAg test was 15,552. Individuals with baseline diagnoses of hepatitis or cirrhosis (n=1,748) and cancer (n=98) at baseline were excluded, leaving 13,723 participants for the analysis. For prediction models involving blood-based biomarkers, the sub-population analysis included 394 participants with GGT and ALT data, applying the same exclusion criteria.

3. **Diabetes patient:** The total number of participants with diabetes was 30,301. Exclusion criteria for diabetes patients encompassed baseline diagnoses of hepatitis or cirrhosis (n=398), cancer (n=294), or positive HBsAg results (n=1,190) at the study baseline. The total number of diabetes patients included in the study was 28,540 individuals. In prediction models utilizing blood-based biomarkers, the sub-population analysis incorporated 1,540 participants with ALT biochemistry data and 1,348 participants with GGT biochemistry data, adhering to the established exclusion criteria.

The vital status of each participant was determined periodically through the China CDC's Disease Surveillance Points (DSP) system and the national health insurance system, supplemented by regular checks against local residential and health insurance records, and by annual active confirmation through street committees or village administrators. In addition, information about the occurrence of major diseases and any episodes of hospitalization was collected through linkages, using each participant's unique national identification number, with disease registries and national health insurance claims databases, which have almost universal coverage in the study areas. All events were coded using the International Classification of Diseases, 10th revision (ICD-10), by trained staff who were blinded to baseline information and reviewed centrally for consistency[2]. The ICD-10 of chronic liver disease (CLD) events in CKB is reported in **Method S4**. The present study included incident liver disease from enrollment until December 31, 2018, by which time a total of 56,552 (11.03%) participants had died and 4,028 (0.78%) were lost to follow-up.

Method S3 The measurement and definition of CKB predictor variables

Predictor	Measurements	Units and definitions
Sex	Questionnaire	Female/male
Age	Questionnaire	Unit: year
Body mass index (BMI)	Formula: BMI=weight(kg)/height(m)^2. Instrument: height (self-made instrument), weight (TANITATBF-300GS body composition analyzer); accuracy: one decimal place.	Unit: kg/m^2
Waist circumference (WC)	Measurement position: midpoint level of the line between the anterior superior iliac crest and the 12th costal edge; tool: plastic soft tape; precision: one after the decimal point; measurement time: at the end of normal expiration.	Unit: cm
Waist-to-hip ratio	Measurement position: maximum extension of hip; tool: soft tape; accuracy: one decimal place.	Unit: cm
Systolic Blood Pressure (SBP)	Instrument: UA-799 digital sphygmomanometer. Measurement time: sit for 5 min, repeat twice, and take the average value. If the difference is >10 mmHg, take the third measurement and average over the last two measurements.	Unit: mmHg
Diastolic blood pressure (DBP)	Same SBP	Unit: mmHg
Self-reported history of cirrhosis/chronic hepatitis	Questionnaire<br>Question: Have you been diagnosed with cirrhosis/chronic hepatitis by a doctor in a township/district hospital?	(Yes/no)

Predictor	Measurements	Units and definitions
Self-reported history of diabetes	Questionnaire<br>Question: Have you been diagnosed with diabetes by a doctor in a township/district hospital?	(Yes/no)
Newly detected diabetes during field investigation	Measurement tool: SureStep Plus System (LifeScan); Sample: Venous blood specimen. The object answered "yes" in question 1 was repeated if random blood glucose was 7.8-11.0 mmol/L.	Question 1 answered "no", the newly detected diabetes at site investigation was defined as follows: (1) random glucose value 7.0 mmol/L with fasting time 8 h; or (2) random glucose value 11.1 mmol/L with fasting time < 8 h; or (3) repeated random glucose measurement 7.0 mmol/L on the next day.
Triglyceride (TG)	Measurement of TG Beckman Chemistry Information Sheets: BLOSR6x118.04 2015-04. Beckman reagent (REF OSR6*118), analytical wavelength of primary 660 nm, secondary 800 nm, sample size of 1.6 μL, reagent volume(R1) 66 μL dilution of 57 μL, reagent volume(R2) 17 μL, dilution of 10 c.	Unit: Millimoles/Little
Total cholesterol (TC)	Reference for TC Beckman Coulter Cholesterol Chemistry Information Sheets: BLOSR6X16.07 2015-05. Beckman reagent (REF OSR6116), analyzed at primary 540 nm, secondary 600 nm, sample size of 1.6 μL, reagent volume(R1) of 24 μL, at 96 μL dilution.	Unit: Millimoles/Little
Smoking status	Questionnaire<br>(1) Current frequency of smoking:<br>Question 2 of I: How much time do you smoke once now? (A=non-smoking; B=occasional; C=most days; D=daily)<br>(2) Frequency of past smoking:<br>Question I 6: In the past, have you ever had the habit of smoking every day? (A=never; B=occasionally; C=most days; D=daily)<br>(3) Daily smoking volume	The current daily smoker was defined as: Question 2=D (Yes/No)<br>Quitters were defined as: Question 2=A and Question 6=B+C+D (Yes/No)<br>Daily smoking:<br>Tobacco content conversion: cigarette contains 1 gram of tobacco/cigarette, cigar contains 2 grams of tobacco/cigarette, grams of pipe/hookah, hand cigarette/dry tobacco are converted by the reported value.
Frequency of physical exercise	Questionnaire<br>Question 9: In the past year, how often do you usually take a	Metabolic equivalent (MET) for different types of physical activity was taken from the 2011 Physical Activity Outline.

Predictor	Measurements	Units and definitions
	physical exercise in your spare time? (A=never or almost absent; B=1-3 times/month; C=1-2 times/week; D=3-5 times/week; E=exercise every day or almost every day)	Unit: MET-hour/day

Method S4 ICD-10 of CLD events in CKB

ICD-10	Diagnosis	No. cases
**HCC**		
C22.0	Liver cell carcinoma	354
C22.9	Malignant neoplasm of liver and intrahepatic bile ducts, unspecified	2,916
**Total**		3,017
**CLD**		
K70	alcoholic liver disease, ALD	329
K72.0	Acute liver failure	35
K72.1	Chronic liver failure	12
K72.9	Liver failure unspecified	251
K74	fibrosis and cirrhosis of liver	2,708
K76.7	Hepatorenal syndrome	31
I85.0	Esophageal varices with bleeding	103
I85.9	Esophageal varices without bleeding	45
C22	Liver cancer	3,268
**Total**		5,565

Method S5 Predictors and equations for included models

Predictors and equations for included models

HCC

1. HLI

(1) Predictors: BMI, lifetime alcohol consumption, diet score, physical activity, smoking, hepatitis infection, diabetes

Predictor definition:

a. BMI (continuous, kg/m^2), average lifetime alcohol intake (continuous, grams per day).
b. Diet score (The diet score combined six dietary items including cereal fiber, red and processed meats, ratio of polyunsaturated to saturated fatty acids, margarine (used as a surrogate marker for trans-fat from industrial sources), glycemic load, and fruits and vegetables. continuous).
c. Physical activity (continuous metabolic equivalents of task in metabolic equivalent hours per week).
d. Smoking (never, former smokers quit >10 y, former smokers quit c10 y, current smokers ≤15 cigarettes/d, current smokers >15 cigarettes/d).
e. Hepatitis infection (yes/no), and self-reported diabetes at baseline (yes/no). These are the components of an HLI used in EPIC, modified to include hepatitis and diabetes status.
f. Average lifetime alcohol intake was used instead of alcohol intake at recruitment to address potential bias related to reverse causality.

(2) Equation:

Modified HLI variable	Scoring details
**BMI (kg/m^2)**	
5th quintile (c30)	0
4th quintile (26-29.9)	1
3rd quintile (24-25.9)	2
2nd quintile (22-23.9)	3
1st quintile (<22)	4
**Lifetime alcohol consumption(g/day)**	
m: >30; w: >20	0
m: 15-30; w: 10-20	1
m: 5-15; w: 5-10	2
0.1-5	3
Never	4
**Diet score**	
1st quintile (6-21)	0
2nd quintile (22-25)	1
3rd quintile (26-28)	2
4th quintile (29-33)	3
5th quintile (34-46)	4
**Physical activity (METs-hour/week)**	
1st quintile (<45)	0
2nd quintile (46-69)	1
3rd quintile (70-96)	2
4th quintile (97-133)	3
5th quintile (>=134)	4
**Smoking**	
Current smokers, >15 cigarettes/day	0
Current smokers, <=15 cigarettes/day	1
Former smokers, quit <=10 years	2
Former smokers, quit >10 years	3
Never	4
**Hepatitis Infection**	
Yes	0
No	4
**Diabetes at baseline**	

Yes	0
No	4

(4) Equation in CKB:
3.9369385+0.081874441*bmi_q
-0.19685988*pa_q
-0.097734815*fruit
-0.22615716*cig_category
-0.047279255*alcohol_cat
-0.55157654*hepatitis
-0.16193887*diabetes

2.1 Wen 1-2012

(1) Predictors: sex, age, smoking, drinking, physical activity, diabetes
Predictor definition:
a. Smoking was classified by the number of pack-years (i.e. daily cigarette quantity*duration in years) among every smoker.
b. Alcohol consumption was classified into "regular drinkers" (those who consumed ≥2 drinks/day on ≥3 days/week) and "occasional drinkers" (those who consumed <2 drinks/day on <3 days/week).
c. Physical activity: regarding volume of leisure time physical activity (LTPA) (i.e. the product of intensity measured as metabolic equivalent tasks [MET]*duration of exercise in hours), the MET-hour per week of each individual was classified as: inactive (<3.75 MET-hour), low-active (3.75-7.49 MET-hour), and active (≥7.5 MET-hour; this group met the current LTPA recommendation)

(2) Equation:

Wen 1-2012 variable	Scoring details
**Sex**	
Female	0
Male	2
**Age**, **year**	
20-39	0
40-59	2
≥60	6
**Smoking, pack-years**	
0	0
1-9.9	1
≥10	1
**Drinking**	
None or occasional	0
Regular	1
**Physical Activity, MET-hour**	
<3.75	0
3.75-7.49	-1
≥7.5	-1
Diabetes	
No	0
Yes	2

(3) Equation in CKB:
-1.0610638+0.24293881*data$age_category
+0.31523732*data$sex
+0.096166441*data$alc_category
+0.17316124*data$act_category
+0.47848429*data$diabetes
+0.37469387*data$cig_category

2.2 Wen 2-2012

(1) Predictors: sex, age, AST, ALT

(2) Equation:

Wen 2-2012 variable	Scoring details
**Sex**	
Female	0

Male	2
**Age, year**	
20-39	0
40-59	2
≥60	6
**AST, IU/L**	
<25	0
25-39	5
40-59	9
≥60	13
**ALT, IU/L**	
<25	0
≥25	2

(3) Equation in CKB:
-1.2420188+0.15651695*dat$age_category
+0.3586473*dat$sex
-0.076442133*dat$ALT_category
+0.12480102*dat$AST_category

2.3 Wen 3-2012

(1) Predictors: sex, age, smoking, drinking, physical activity, diabetes, AST, ALT

(2) Equation:

Wen 3-2012 variable	Scoring details
**Sex**	
Female	0
Male	1
**Age, year**	
20-39	0
40-59	2
≥60	6
**Smoking, pack-years**	
0	0
1-9.9	1
≥10	1
**Drinking**	
None or occasional	0
Regular	1
**Physical Activity, MET-hour**	
<3.75	0
3.75-7.49	0
≥7.5	-1
**Diabetes**	
No	0
Yes	1
**AST, IU/L**	
<25	0
25-39	4
40-59	9
≥60	12
**ALT, IU/L**	
<25	0

≥25	1

(3) Equation in CKB:
-1.2300978+0.15784406*dat$age_category
+0.61661131*dat$sex
-0.21056898*dat$ALT_category
+0.14130867*dat$AST_category
+0.11230019*dat$alc_category
+0.089372728*dat$act_category
+0.024911687*dat$diabetes
+0.071970998*dat$cig_category

3. DM-HCC

(1) Predictors: age, GGT, TG

(2) Equation:

DM-HCC variable	Scoring details
Age, years	
>65	11
≤65	0
GGT (IU/L)	
>80	16
41-80	8
≤40	0
TG (mg/dL)	
<150	6
≥150	0

(3) Equation in CKB:
-0.50559487+0.071973603*dat$age_category
+0.10426216*dat$GGT_category
+0.038324225*dat$TG_category

4. Li-2018

(1) Predictors: age, gender, smoking habit, SGPT, variation of HbA1c, comorbidity, antidiabetes medications, antihyperlipidemia medication, THR
Predictor definition:
a. SGPT: serum ALT
b. HbA1c: RPG
c. Medication: as a user of specific medication when his/her number of prescription days for each specific drug was greater than 3 months. The anti diabetes medications of individual patients were further classified into 4 categories: no medication, oral anti diabetes drugs, insulin monotherapy, and insulin plus oral anti diabetes drugs.
d. Antihyperlipidemia medications considered were statins and fibrates.
e. THR: total/high-density lipoprotein (HDL) cholesterol ratio

(2) Equation:
Age (-2 to 8)
+Gender (0 if female, 2 if male)
+Smoking habit (0 if no smoking, 2 if smoking)
+SGPT (u/l) (0 if 6-45, 6 if <6 or >45)
+Variation of HbA1c (%) (0 if <8.5, 0 if 8.5-17.5, 1 if >17.5)
+Comorbidity (9 if Liver cirrhosis, 4 if Hepatitis B, 3 if Hepatitis C)
+Antidiabetes medications (0 if no medication, 0 if oral only, 2 if insulin, 3 if insulin+oral agent)
+Antihyperlipidemia medication:
-No medication:
-THR: male <5; female <4.5: Score 0
-THR: male 5-9.4; female 4.5-7: Score-1
-THR: male ≥9.5; female ≥7: Score 2
-Yes medication:
-THR: male <5; female <4.5: Score-2
-THR: male 5-9.4; female 4.5-7: Score-4
-THR: male ≥9.5; female ≥7: Score-3

(3) Equation in CKB:
-2.0077928+1.6373314*(dat$age_category=="-1")

-0.084629372*(dat$age_category=="0")
+1.5179949*(dat$age_category=="1")
+1.355421*(dat$age_category=="2")
+1.378663*(dat$age_category=="3")
+1.3509516*(dat$age_category=="4")
+1.7908917*(dat$age_category=="5")
+2.0393897*(dat$age_category=="6")
+2.2834965*(dat$age_category=="7")
-2.4928856*(dat$age_category=="8")
+0.37026501*dat$sex
-0.009137821*dat$smoke_category
+0.2810228*dat$SGPT_category+
0.76331689*dat$HbA1c_category
+0.39906606*dat$comorbidity_category
-2.095294*dat$antidiabetes_category
-0.064815973*dat$THR_category

5. Sinn-2020

(1) Predictors: age, gender, body mass index, smoking, diabetes,total cholesterol, ALT
Predictor definition:
a. BMI was classified according to Asian-specific criteria (underweight, BMI of <18.5 kg/m^2; normal weight, BMI of 18.5 to 22.9 kg/m^2; overweight, BMI of 23 to 24.9 kg/ m^2; and obese, BMI ≥25 kg/m^2).
b. Pre-hypertension was defined as a systolic blood pressure 130-<140 mmHg or a diastolic blood pressure 85-<90 mmHg at the baseline screening.
c. Hypertension was defined as the presence of at least one I10-I13 or I15 code during the year preceding the screening, or a systolic blood pressure 140 mmHg or a diastolic blood pressure 90 mmHg at the baseline screening.
d. Pre-diabetes was defined as a fasting glucose level of 100-<126 mg/dL at the baseline screening. Diabetes was defined as the presence of at least one E11-E14 code or a fasting glucose level of 126 mg/dL at the baseline screening.
e. Dyslipidaemia was defined as the presence of an E78 code or a total cholesterol level of >240 mg/dL at the baseline screening.
f. ALT level was classified into three groups: low (<30 U/L for males and <20 U/L for females), mildly elevated (30-89 U/L for males and 20-59 U/L for females) and elevated (90 U/L for males and 60 U/L for females)
g. Current alcohol consumption was categorized into none or modest (<30 g/day in men and <20 g/day in women).

(2) Equation:

DM-HCC variable	Scoring details
**Age**	
<50	0
50-59	3
60-69	6
≥70	7
**Sex**	
Female	0
Male	2
**Smoking**	
Never	0
Past	0
Current	1
**Diabetes**	
No diabetes	0
Pre-diabetes	0
Diabetes	1
**Total cholesterol(mg/dL)**	
<200	2
≥200	0
**Alanine aminotransferasea**	
Low	0

Mildly elevated	2
Elevated	6

(3) Equation in CKB:
-1.0888972+0.15591552*age_category
+0.44051948*sex
-0.042037495*current_smoking
-0.053377929*Diabetes
+0.159525*ALT_category
+1.3294444*Total_cholesterol

6. Cao-2021

(1) Predictor: age, sex, liver diseases in mother, BMI, alcohol consumption, psychological trauma
Predictor definition:
a. Liver diseases in mothers: including liver cancer, chronic hepatitis B, and other liver disease.

(2) Equation:
F(t)=1-S(t)exp(f,M);
f,M=0.058×(age in years)
+0.879 × (Female=0, Male=1)
+0.608 × (liver diseases in mother: Yes=1, No=0)
+0.380 × (BMI<25=1, BMI ≥25=0)
+0.753 × (alcohol consumption ≥550 g/week ethanol: Yes=1, No=0)
+0.480 × (psychological trauma: Yes=1, No=0)-4.010.

CLD

1.1 CLivD score(nonlab)

(1) Predictors: age, WHR, alcohol use (spline variable), GGT, diabetes, smoking status, sex*GGT, sex*smoking status

(2) Equation:
```
data$modellab <-(-6.7922721+0.044744302*data$AGE
+0.32961593*(data$WHR*10)+0.19860813*data$ALCOHOL
-0.0082096868*pmax(data$ALCOHOL-0.1,0)^3
+0.010575035*pmax(data$ALCOHOL-1,0)^3
-0.002004756*pmax(data$ALCOHOL-3,0)^3
-0.00033998925*pmax(data$ALCOHOL-9,0)^3
-2.0602882e-05*pmax(data$ALCOHOL-
33,0)^3+0.011813962*data$GGT+0.18721469*(data$SEX=="2")
+0.55249734*(data$DIABETES=="1")
+0.74679941*(data$SMOKING=="1")
+0.0054325769*data$GGT*(data$SEX=="2")
-0.64903176*(data$SEX=="2")*(data$SMOKING=="1"))
```

(3) Equation in CKB:
```
-1.7996042-0.13849959*data$alcohol_week
+0.0081373039*pmax(data$alcohol_week-1,0)^3
-0.016951927*pmax(data$alcohol_week-3,0)^3
+0.0090844935*pmax(data$alcohol_week-5,0)^3
-0.00025840613*pmax(data$alcohol_week-9,0)^3
-1.1464752e-05*pmax(data$alcohol_week-33,0)^3
+0.050087058*data$age
+0.45957616*data$whr
+0.42501114*data$diabetes
-0.27723986*data$smoking
-0.52580546*data$sex
+0.017439031*data$smoking*data$sex
```

1.2 CLivD score(lab)

(1) Predictors: age, WHR, alcohol use (spline variable), diabetes, smoking status, sex*smoking status

(2) Equation:
```
data$modelnonlab <-(-8.0940103+0.044177151*data$AGE+0.48927753*(data$WHR*10)
+0.19222894*data$ALCOHOL-0.00015029544*pmax(data$ALCOHOL-0.1,0)^3-
0.0021265611*pmax(data$ALCOHOL-1,0)^3+0.0029832769*pmax(data$ALCOHOL-3,0)^3-
0.00068765143*pmax(data$ALCOHOL-9,0)^3-1.8769011e-05*pmax(data$ALCOHOL-
33,0)^3+0.69669285*(data$DIABETES=="1")
```

+0.75968055*(data$SMOKING=="1")<br>+0.63248362*(data$SEX=="2")-0.59146649*(data$SMOKING=="1")*(data$SEX=="2"))
(3) Equation in CKB:<br>-1.0759261-0.04427105*data$alcohol_week<br>+4.3147139e-05*pmax(data$alcohol_week-0.1,0)^3<br>-9.0209853e-05*pmax(data$alcohol_week-15,0)^3<br>+5.0565399e-05*pmax(data$alcohol_week-30,0)^3<br>-3.502685e-06*pmax(data$alcohol_week-48,0)^3<br>+0.044255473*data$age<br>-1.1958386*data$whr<br>+0.01055964*data$ggt<br>-0.49321834*data$sex<br>+0.030192835*data$diabetes<br>+0.099859348*data$smoking<br>+0.0041691027*data$ggt*data$sex<br>-0.077726792*data$sex*data$smoking
**2. BARD**
(1) Predictors: BMI, AST, ALT, T2DM
(2) Equation:<br>(1 if BMI >28 kg/m^2)<br>+(2 if AST/ALT ratio >0.8)<br>+(1 if T2DM)
(3) Equation in CKB:<br>-0.66609144-0.1319787*data$bmi_c<br>+0.33679484*data$diabetes_c<br>+0.34728017*data$ast_c
**3. dAAR**
(1) Predictors: age, ALT, AST
(2) Equation:<br>dataset$riskscore1<-10.129915+0.039811813*dataset$AGE<br>+0.25387407*dataset$ALT<br>-0.0023607234*pmax(dataset$ALT-11,0)^3<br>+0.0079492072*pmax(dataset$ALT-17,0)^3<br>-0.0076811579*pmax(dataset$ALT-22,0)^3<br>+0.0021985068*pmax(dataset$ALT-30,0)^3<br>-0.00010583268*pmax(dataset$ALT-58,0)^3<br>+3.5333535*dataset$astalt<br>-7.3473709*pmax(dataset$astalt-0.63,0)^3<br>+32.911587*pmax(dataset$astalt-0.92,0)^3<br>-44.937707*pmax(dataset$astalt-1.14,0)^3<br>+21.786619*pmax(dataset$astalt-1.41,0)^3<br>-2.4131284*pmax(dataset$astalt-2.13,0)^3
(3) Equation in CKB:<br>-10.334179-0.12283087*data$ALT<br>+0.0004243091*pmax(data$ALT-0.1,0)^3<br>-0.0012106484*pmax(data$ALT-11,0)^3<br>+0.00090314578*pmax(data$ALT-17,0)^3<br>-0.00031645783*pmax(data$ALT-22,0)^3<br>+0.00023916006*pmax(data$ALT-30,0)^3<br>-3.9508734e-05*pmax(data$ALT-58,0)^3<br>+10.649273*data$astalt<br>-4.8201285*pmax(data$astalt-0.1,0)^3<br>+7.4526922*pmax(data$astalt-0.63,0)^3<br>+10.855356*pmax(data$astalt-0.92,0)^3<br>-21.971644*pmax(data$astalt-1.14,0)^3<br>+10.031624*pmax(data$astalt-1.41,0)^3<br>-1.5478998*pmax(data$astalt-2.13,0)^3<br>+0.03380337*data$AGE
**4. CAP-B**

(1)	Predictors: age, sex, income, chronic hepatitis C, diabetes mellitus, statin exposure, antiplatelet exposure, smoking, ALT, GGT<br>Predictor definition:<br>a. Income: medical aid <25%, low income=25-50%, intermediate=50-75%<br>b. Disease history: chronic hepatitis C; diabetes mellitus<br>c. Defined statin exposure as having filled at least 2 prescriptions within a six-month period,<br>d. Aspirin exposure as a prescription for at least once within 2 years prior to the index date<br>e. Smoking: smoking was based on the current status (i.e. yes or no) by patient health questionnaires<br>f. GGT*alcohol consumption:<br>g. Drinking: alcohol drinking was based on the current status (i.e. yes or no) by patient health questionnaires<br>h. Normal GGT: 3-50 U/L
(2)	Equation:<br>0.677*(male)<br>+0.085*(age)<br>+0.310*(medical aid/<25%)<br>+0.249*(low income)<br>+0.146*(intermediate income)<br>+0.066*(chronic hepatitis C)<br>+0.406*(diabetes mellitus)<br>-0.426*(statin exposure)<br>+0.025*(antiplatelet exposure)<br>+0.230*(smoking)+0.539*(ALT >35)<br>+0.079*(normal GGT and drinking or abnormal GGT and & nondrinking)<br>+0.532*(abnormal GGT and drinking)+0.426<br>P(t)=1−S0(t)exp(risk score−0.43)

Table S1 Bias assessment

No	Study	ROB				Applicability			Overall	
		Participants	Predictors	Outcome	Analysis	Participants	Predictors	Outcome	ROB	Applicability
1	Fredrik Åberg (2022)	Low	Low	Low	Low	Low	Low	Low	Low	Low
2	Vincent Wai-Sun Won (2010)	Low	Low	Low	High	Low	Low	Low	High	Low
3	Hwai-I Yan (2010)	Low	Low	Low	Unclear	Low	Low	Low	Unclear	Low
4	Man-Fung Yuen (2009)	Low	Low	Low	Unclear	Low	Low	Low	Unclear	Low
5	Hwai-I Yang (2011)	Low	Unclear	Low	Unclear	Low	Low	Low	Unclear	Low
6	Masayuki Kurosaki (2012)	Low	Unclear	Low	High	Low	Low	Low	High	Low
7	Takehiro Michikawa (2012)	Low	Low	Unclear	Unclear	Low	Low	Low	Unclear	Low
8	Chi-Pang Wen (2012)	Low	Low	Low	Unclear	Low	Low	Low	Unclear	Low
9	Juan Carlos Gavilán (2013)	Low	Unclear	Low	High	Low	Low	Low	High	Low
10	Mei-Hsuan Lee (2013)	Low	Unclear	Low	Unclear	Low	Low	Low	Unclear	Low
11	Yu-Ju Lin (2013)	Low	Low	Low	High	Low	Low	Low	High	Low
12	Mei-Hsuan Lee (2014)	Low	Low	Low	High	Low	Low	Low	High	Low
13	Talita Duarte-Salles (2016)	Low	Unclear	Low	Unclear	Low	Low	Low	Unclear	Low
14	Beomseok Suh (2015)	Low	Unclear	Low	Unclear	Low	Low	Low	Unclear	Low
15	Chunsun Fan (2019)	Low	Unclear	Unclear	High	Low	Low	Low	High	Low
16	Won Keun Si (2016)	Low	Unclear	Low	High	Low	Low	Low	High	Low
17	Adeel A. Butt (2017)	Low	Unclear	High	Unclear	Low	Low	Low	High	Low
18	Wei-Yi Kao (2017)	Low	Unclear	Unclear	Unclear	Low	Low	Low	Unclear	Low
19	M. A. Konerman (2017)	Low	Low	Low	Unclear	Low	Low	Low	Unclear	Low
20	Yeon Seok Seo (2017)	Low	Unclear	Low	High	Low	Low	Low	High	Low
21	Hwai-I Yang (2016)	Low	Low	Low	Low	Low	Low	Low	Low	Low
22	Tsai-Chung Li (2018)	Low	Low	Low	Unclear	Low	Low	Low	Unclear	Low
23	Lilian Yan Liang (2021)	Low	Low	Low	High	Low	Low	Low	High	Low
24	Dong Hyun Sinn (2020)	Low	Low	Low	High	Low	Low	Low	High	Low
25	Dong Hyun Sinn (2019)	Low	Low	High	High	Low	Low	Low	High	Low
26	Fredrik Åberg (2021)	Low	Unclear	Low	Unclear	Low	Low	Low	Unclear	Low
27	Yuting Wang (2021)	Low	Low	Unclear	High	Low	Low	Low	High	Low
28	Mathias Daheim (2016)	Unclear	Unclear	Unclear	High	Low	Low	Low	High	Low

No	Study	ROB				Applicability			Overall	
		Participants	Predictors	Outcome	Analysis	Participants	Predictors	Outcome	ROB	Applicability
29	Ae Jeong Jo (2022)	Low	Low	Low	High	Low	Low	Low	High	Low
30	Hannes Hagström (2019)	Low	Unclear	Low	Unclear	Low	Low	Low	Unclear	Low
31	Dong Hyun Sinn (2016)	High	Unclear	Low	High	Low	Low	Low	High	Low
32	Chansik An (2021)	Low	Low	Low	Low	Low	Low	Low	Low	Low
33	Chengxiao Yu (2021)	Low	Low	Low	High	Low	Low	Low	High	Low
34	Thanachote Kamalapirat (2021)	Low	Unclear	Unclear	High	Low	Low	Low	High	Low
35	Alessandra Porto de Macedo Costa (2022)	Low	Unclear	Low	High	Low	Low	Low	High	Low
36	Nada Assi (2018)	Low	Low	Unclear	Unclear	Low	Low	Low	Unclear	Low
37	Tai-Chung Tseng (2017)	Low	Unclear	Low	Unclear	Low	Low	Low	Unclear	Low
38	Philip J. Johnson (2022)	Low	Unclear	Low	Low	Low	Low	Low	Low	Low
39	Do Young Kim (2013)	Low	Unclear	Low	High	Low	Low	Low	High	Low
40	Monica A. Konerman (2019)	Low	Unclear	High	High	Low	Low	Low	High	Low
41	An K. Le (2021)	Low	Low	Low	Unclear	Low	Low	Low	Unclear	Low
42	Maomao Cao (2021)	Low	Low	Unclear	Unclear	Low	Low	Low	Unclear	Low
43	Hannes Hagström (2019)	Low	Low	Unclear	High	Low	Low	Low	High	Low
44	Jae Seung Lee (2021)	Low	Unclear	Low	High	Low	Low	Low	High	Low
45	Thierry Poynard (2019)	Low	Unclear	Low	High	Low	Low	Low	High	Low
46	Jonathan Thomas (2022)	Low	Low	Unclear	Unclear	Low	Low	Low	Unclear	Low
47	Grace Lai-Hung Wong (2014)	Low	Unclear	Low	High	Low	Low	Low	High	Low
48	Thierry Poynard (2021)	Low	Unclear	Unclear	Unclear	Low	Low	Low	Unclear	Low
49	Hsiao-Hsien Rau (2016)	Low	Low	Low	Unclear	Low	Low	Low	Unclear	Low
50	Seung Hwan Shin (2015)	Low	Unclear	Low	High	Low	Low	Low	High	Low
51	Namyoung Paik (2018)	Low	Unclear	Unclear	Unclear	Low	Low	Low	Unclear	Low
52	Kyu Sik Jung (2015)	Low	Unclear	Low	Unclear	Low	Low	Low	Unclear	Low
53	Mi Young Jeon (2018)	Low	Unclear	Low	Unclear	Low	Low	Low	Unclear	Low
54	Mahmoud Abu-Amara (2016)	Low	Unclear	Unclear	Unclear	Low	Low	Low	Unclear	Low
55	W. P. Brouwer (2017)	Low	Unclear	Low	Unclear	Low	Low	Low	Unclear	Low
56	Zhongxian Poh (2016)	Low	Unclear	Low	High	Low	Low	Low	High	Low
57	Namkyu Kang (2021)	Low	Low	Low	High	Low	Low	Low	High	Low

Table S2 Predictors of prediction models for general population

Model	Demographic		Lifestyle							Blood-based biomarkers														Others
	Age	Sex	Alcohol	BMI	Diet	PA	Smoking	WHR	Diabetes	AFP	ALB	ALP	ALT	AST	BIL	GGT	Glu	HBsAg	HBeAg	HBV DNA	TC	TG	PLT	
AGED	√	√											√						√	√				
An-2021	√	√		√					√				√			√			√	√	√			income etc.‡
CKB-PLR	√	√	√	√		√	√		√								√							Family history of cancer etc.§
CLivD (lab)	√	√	√				√	√	√							√								
CLivD (non-lab)	√	√	√				√	√	√															
dAAR	√												√	√										
Duarte-Salles 1-2016										√														OPN
Duarte-Salles 2-2016										√		√		√		√								OPN
Duarte-Salles 3-2016												√		√		√								
Duarte-Salles 4-2016												√		√		√								OPN
Elaborate Base Model	√	√	√	√	√	√			√															height, general health status
HLI			√	√	√	√	√		√															hepatitis
LCR1	√	√														√								APOA1, Hp, A2M
LCR2	√	√								√						√								APOA1, Hp, A2M
LFS											√	√	√	√	√	√								
LFS+lifestyle signature			√	√	√	√	√		√															hepatitis
LFS+metabolic signature																	√							Metabolic signature¶
Metabolic signature											√	√	√	√	√	√	√							Metabolic signature¶
Michikawa	√	√	√	√					√									√						coffee consumption, Anti-HCV Ab
Parsimonious Base Model	√	√	√	√	√	√			√															general health status
Sinn-020	√	√					√		√				√								√			
Sung(established)-2012								√					√				√					√	√	
Sung(new)-2012								√					√				√					√	√	
Wen 1-2012	√	√	√			√	√		√															
Wen 1'-2012	√	√	√			√	√		√															
Wen 2-2012	√	√											√	√										

Model	Demographic		Lifestyle							Blood-based biomarkers														Others
	Age	Sex	Alcohol	BMI	Diet	PA	Smoking	WHR	Diabetes	AFP	ALB	ALP	ALT	AST	BIL	GGT	Glu	HBsAg	HBeAg	HBV DNA	TC	TG	PLT	
Wen 2'-2012	√	√											√	√										
Wen 3-2012	√	√	√			√	√		√				√	√										
Wen 3'-2012	√	√	√			√	√		√				√	√										
Wen 4-2012	√	√	√			√	√		√	√			√	√				√						
Wen 4'-2012	√	√	√			√	√		√	√			√	√				√						
Wen 5'-2012	√	√	√			√	√		√	√			√	√				√						Anti-HCV Ab

‡: Income, family history of chronic liver disease, disease history (chronic liver disease, chronic hepatitis virus infection, HIV infection, dyslipidemia, or schizophrenic/delusional disorders or mental disorders due to psychoactive substance use)
§: Family history of cancer, residential area, education, disease history (cancer, gallstone or gallbladder disease), cirrhosis
¶: Glutamic acid, Hexoses, SMC16:1, SM(OH)C14:1, SM(OH)C22:2, LysoPC aC28:1, PC aeC30:2
A2M: alpha2 - macroglobulin; AAR: aspartate-to-alanine aminotransferase ratio; AFP: alpha-fetoprotein; ALB: Albumin; ALP: alkaline phosphatase; ALT: alanine transaminase; Anti-HCV Ab: Anti-Hepatitis C Virus Antibody; APOA1: apolipoprotein A1; AST: aspartate transaminase; BIL:Bilirubin; GGT: gamma-glutamyl transferase; Glu: Glutamic acid; HBeAg: Hepatitis B e Antigen; HBsAg: Hepatitis B Surface Antigen; Hp: haptoglobin; PA: physical activity; PLT: Platelet count; TC: total cholesterol; TG concentration: triglyceride concentration; WC: waist circumference; WHR: waist-to-hip rate

Table S3 Predictors of prediction models for HBV infected individuals

Model	Demographic		Lifestyle			Personal and family history			Blood-based biomarkers								Others
	Age	Sex	Alcohol	BMI	Smoking	Diabetes	Cirrhosis	Family history	AFP	ALB	ALT	AST	GGT	HBeAg	HBV DNA	PLT	
Cao-2021	√	√	√	√				√‡									Psychological trauma
CAP-B Score	√	√	√		√	√					√		√				income, CHC, statin exposure, anitplatelet exposure
D^{2}AS	√	√									√			√	√		
GAG-HCC	√	√					√								√		core promoter mutations
GAG-HCC (non-core promoter mutations)	√	√					√								√		
Le-2021	√	√				√					√	√		√			antiviral treatment
Lee-2013	√	√						√			√			√	√		
Liang score	√	√													√		FIB-4
Lin-Model I	√	√								√	√			√	√		
Lin-Model II	√	√							√		√	√	√				alpha-1 globulin
Lin-Model III	√	√							√	√	√	√	√		√		alpha-1 globulin
LSM-HCC	√									√					√		Liver stiffness
LSPS																√	Liver stiffness, spleen diameter
MALE-ABCD	√								√				√				
Methylation Profile																	three methylated sites§
Model 1-regression model	√	√	√					√			√			√			
Model 2-regression model	√	√	√					√			√			√	√		
Model 3-regression model	√	√	√					√			√			√	√		HBV genotype
NGM1-HCC	√	√	√					√			√			√			
NGM2-HCC	√	√	√					√			√			√	√		
NGM3-HCC	√	√	√					√			√			√	√		HBV genotype
REACH-B	√	√									√			√	√		
REACH-B IIa	√	√									√			√	√		
REACH-B IIb	√	√									√			√			
RWS-HCC	√	√					√		√								
Sinn-2019	√	√					√				√			√		√	
Transient elastography-based risk estimation	√	√													√		liver stiffness

Model	Demographic		Lifestyle			Personal and family history			Blood-based biomarkers								Others
	Age	Sex	Alcohol	BMI	Smoking	Diabetes	Cirrhosis	Family history	AFP	ALB	ALT	AST	GGT	HBeAg	HBV DNA	PLT	
Won-2010	√						√			√					√		

‡: Liver diseases in mothers

§: cg00300879, cg06872964, and cg07080864

AFP: α-Fetoprotein; ALB: Albumin; ALT: Alanine Aminotransferase; AST: Aspartate Aminotransferase; CHC: chronic Hepatitis C; GGT: Gamma-Glutamyl Transferase; PLT: Platelet

Table S4 Predictors of prediction models for other populations

Model	Demographic		Lifestyle		Personal and family history				Blood-based biomarkers								other
	Age	Sex	BMI	Smoking	Diabetes	Cirrhosis	Hepatitis	Fatty liver	ALB	ALT	AST	AFP	BR	GGT	HBsAg	PLT	
ANN-model 1	√	√				√	√	√									alcoholic cirrhosis etc.‡
ANN-model 2	√	√				√	√	√									alcoholic cirrhosis etc.§
ANN-model 3	√	√				√	√	√									nonalcoholic cirrhosis etc.¶
CNN																	
CRS																	seven SNPs
CS boosting model																	
DM-HCC	√													√			TG
GRU																	
Li-2018	√	√		√		√	√			√							Hba1C, antidiabetes medication, antihyperlipidemia medication and THR
HCC-4 Risk Score	√											√				√	gammaglobulin
HCC-RS	√	√			√				√	√	√		√			√	Cardiovascular disease etc.•
Liver Volume Index														√			Volume Index
longitudinal boosting model																	
longitudinal Cox model																	
LR-model 1	√	√				√	√	√									alcoholic cirrhosis etc.‡
LR-model 2	√	√				√	√	√									alcoholic cirrhosis etc.§
LR-model 3	√	√				√	√	√									nonalcoholic cirrhosis etc.¶
LS-Based Model 1	√										√					√	Liver Stiffness
LS-Based Model 2	√										√					√	Liver Stiffness
LS-Based Model 3	√										√					√	Liver Stiffness
LSTM																	
Kurosaki-2012	√								√							√	
Lee-2014	√					√				√	√						HCV RNA and HCV Genotype

‡: alcoholic cirrhosis, nonalcoholic cirrhosis, alcoholic hepatitis, viral hepatitis, other types of chronic hepatitis, alcoholic fatty liver disease, other types of fatty liver disease and hyperlipidemia
§: alcoholic cirrhosis, alcoholic hepatitis, alcoholic fatty liver disease
¶: nonalcoholic cirrhosis, viral hepatitis, other types of chronic hepatitis, other types of fatty liver disease, and hyperlipidemia

•: Cardiovascular disease, Colorectal cancer, Lung cancers, Urinary/renal malignancies, Cervical cancer, Breast cancer, Lymphoma, Chronic kidney disease, Osteopenia, Osteoporosis, Hypertension, Anticoagulants, ACEI/ARB, Antiplatelet agents, Beta blockers, Histamine-2 receptor antagonist, Insulin, Immunosuppressant, Sulphonylurea, Thiazides, Other lipid-lowering agents, Other oral hypoglycaemic agents, Proton pump inhibitor, Potassium sparing diuretics, Statins, Loop diuretics, Metformin, NSAID
AFP: α-Fetoprotein; ALB: Albumin; ALT: Alanine Aminotransferase; AST: Aspartate Aminotransferase; BR: Bilirubin; GGT: Gamma-Glutamyl Transferase; HBsAg: Hepatitis B surface antigen; HbA1c: Hemoglobin A1c; PLT: Platelet; TG: Triglycerides

Table S5 Sensitivity analysis for PLC and HCC

Model	Population	Time horizon (years)	PLC (C22)		HCC (C22.0+C22.9)		HCC (C22.0)	
			Events/total	C-index	Events/total	C-index	Events/total	C-index
HLI	General	10	1,888/478,930	0.65 (0.64-0.67)	1,709/478,930	0.68 (0.67-0.70)	163/478,930	0.68 (0.63-0.73)
Wen 1-2012	General	5	875/478,930	0.72 (0.71-0.74)	793/478,930	0.72 (0.70-0.74)	71/478,930	0.76 (0.71-0.81)
Wen 1-2012	General	10	1,888/478,930	0.72 (0.70-0.73)	1,709/478,930	0.72 (0.70-0.73)	163/478,930	0.76 (0.72-0.80)
DM-HCC (all)	General	5	30/15,818	0.69 (0.60-0.79)	26/15,818	0.68 (0.57-0.79)	3/15,818	0.71 (0.60-0.82)
Li-2018 (all)	General	3	17/17,227	0.80 (0.70-0.90)	12/17,227	0.83 (0.75-0.92)	1/17,227	0.98 (0.98-0.98)
Li-2018 (all)	General	5	32/17,227	0.75 (0.66-0.83)	28/17,227	0.76 (0.68-0.84)	3/17,227	0.92 (0.85-0.99)
Li-2018 (all)	General	10	80/17,227	0.74 (0.68-0.79)	72/17,227	0.74 (0.68-0.80)	8/17,227	0.91 (0.84-0.98)
Sinn-2020	General	5	32/17,227	0.69 (0.60-0.77)	28/17,227	0.70 (0.61-0.78)	3/17,227	0.90 (0.81-0.99)
Sinn-2020	General	10	80/17,227	0.67 (0.61-0.73)	72/17,227	0.66 (0.60-0.72)	8/17,227	0.86 (0.77-0.95)
Wen 2-2012	General	5	32/17,227	0.68 (0.59-0.77)	28/17,227	0.70 (0.62-0.79)	3/17,227	0.82 (0.67-0.97)
Wen 2-2012	General	10	80/17,227	0.67 (0.61-0.73)	72/17,227	0.67 (0.61-0.74)	8/17,227	0.81 (0.66-0.96)
Wen 3-2012	General	5	32/17,227	0.69 (0.60-0.78)	28/17,227	0.71 (0.62-0.80)	3/17,227	0.98 (0.95-0.99)
Wen 3-2012	General	10	80/17,227	0.68 (0.62-0.73)	72/17,227	0.68 (0.61-0.74)	8/17,227	0.85 (0.69-0.99)

Table S6 CLD risk model for 5-year HCC discrimination in the published literature and CKB

Model	Population	Time horizon	Development cohort			Published external validation cohort		CKB	
			Area	Events/total	C-index (95% CI)	Events/total	C-index (95% CI)	Events/total	C-index (95% CI)
**HCC**									
DM-HCC (all)	General	5	EAS	-	-	-	-	26/15,818	0.68 (0.57-0.79)
Li-2018 (all)	General	3	EAS	-	-	-	-	12/17,227	0.83 (0.75-0.92)
Li-2018 (all)	General	5	EAS	-	-	-	-	28/17,227	0.76 (0.68-0.84)
Sinn-2020	General	5	EAS	-	-	-	-	28/17,227	0.70 (0.61-0.78)
Wen 1-2012	General	5	EAS	-	-	-	-	793/478,930	0.72 (0.70-0.74)
Wen 2-2012	General	5	EAS	-	-	-	-	28/17,227	0.70 (0.62-0.79)
Wen 3-2012	General	5	EAS	-	-	-	-	28/17,227	0.71 (0.62-0.80)
Cao-2021	HBV infected	3	EAS	203/110,536	0.73 (0.64-0.82)	-	-	141/13,723	0.75 (0.72-0.79)
Cao-2021	HBV infected	5	EAS	-	-	-	-	250/13,723	0.74 (0.71-0.78)
DM-HCC	T2D	5	EAS	36/2,364	0.86 (0.85-0.88)	-	-	2/1,348	0.75 (0.44-0.99)
Li-2018	T2D	5	EAS	493/21,149	0.80 (0.77-0.83)	-	-	2/1,490	0.99 (0.99-0.99)

Table S7 CLD risk model for 5-year CLD discrimination in the published literature and CKB

Model	Population	Time horizon	Development cohort			Published external validation cohort		CKB	
			Area	Events/total	C-index (95% CI)	Events/total	C-index (95% CI)	Events/total	C-index (95% CI)
**CLD**									
BARD	General	5	EUR	-	-	232/75,303	0.57 (0.52-0.63)	74/17,227	0.55 (0.51-0.59)
dAAR	General	5	EUR	89/18,067	0.80 (0.74-0.85)	343/126,941	0.74 (0.71-0.77)	74/17,227	0.74 (0.68-0.81)
CAP-B	HBV infected	3	EAS	5,781/401,745	0.78 (0.78-0.78)	-	-	8/394	0.91 (0.83-0.98)
CAP-B	HBV infected	5	EAS	10,278/401,745	0.78 (0.78-0.78)	-	-	11/394	0.81 (0.67-0.94)

Table S8 CLD diseases and causes in high income countries and China in 2019

Diseases and causes	High income countries		China	
	Number	Percent	Number	Percent
**Liver cancer (total)**	261,313	-	290,373	-
alcohol use	62,969	24%	25,488	9%
hepatitis B	52,212	20%	193,969	67%
hepatitis C	118,240	45%	41,078	14%
NASH	15,984	6%	12,831	4%
other causes	11,908	5%	17,007	6%
**Cirrhosis and other chronic liver diseases (total)**	161,286,494	-	427,983,626	-
alcohol use	3,421,267	2%	2,417,135	1%
hepatitis B	11,557,512	7%	111,315,938	26%
hepatitis C	10,550,277	7%	19,023,979	4%
NAFLD	134,191,713	83%	293,409,373	69%
other causes	1,565,725	1%	1,817,200	0%

Table S9 Risk factors for CLD in CKB and Western populations

Risk factors	CKB liver cancer	CKB cirrhosis	CUP liver cancer
Alcohol per 10 g/day	1.11 (1.07-1.14)[3]	1.18 (1.14-1.22)[4]	1.04 (1.02-1.06)
BMI per 5 kg/m^2	1.05 (0.94-1.15)[5]	-	1.30 (1.16-1.46)
Diabetes	1.49 (1.30-1.70)[6]	1.81 (1.57-2.09)	-
Physical activity	0.81 (0.71-0.93)	0.76 (0.66-0.88)	0.54 (0.23-1.29)
Smoking	1.29 (1.16-1.42)[7]	1.08 (0.95-1.23)	-

Table S10 Summary of Liver Cancer Screening Strategies

	EASL[8]	AASLD[9]	China screening strategy[10]
**Population**	1. Cirrhotic patients, Child-Pugh stage A and B<br>2. Cirrhotic patients, Child-Pugh stage C awaiting liver transplantation<br>3. Non-cirrhotic HBV patients at intermediate or high risk of HCC[a] (according to PAGE-B[b] classes for Caucasian subjects, respectively 10-17 and ≥18 score points)<br>4. Non-cirrhotic F3 patients, regardless of aetiology may be considered for surveillance based on an individual risk assessment	1. Child-Pugh A-B cirrhosis, any etiology<br>-Hepatitis B<br>-Hepatitis C (viremic or post-SVR)<br>-Alcohol associated cirrhosis<br>-Nonalcoholic steatohepatitis<br>-Other etiologies<br>2. Child-Pugh C cirrhosis, transplant candidate<br>3. Non-cirrhotic chronic hepatitis B<br>-Man from endemic country[c] age >40 years<br>-Woman from endemic country[c] age >50 years<br>-Person from Africa at earlier age[d]<br>-Family history of HCC<br>-PAGE-B score ≥10	Males aged 45-74 and females aged 50-74 who meet at least one of the following criteria:<br>1. Positive Hepatitis B surface antigen (HBsAg)<br>2. History of HCV infection<br>3. History of liver cirrhosis<br>4. A first-degree/second-degree relative with a history of liver cancer
**Method**	Abdominal ultrasound	Ultrasound and AFP	Serum alpha-fetoprotein (AFP) test and abdominal ultrasound
**Frequency**	Every six months	Every six months	

a Patients at low HCC risk left untreated for HBV and without regular six months surveillance must be reassessed at least yearly to verify progression of HCC risk.
b PAGE-B (Platelet, Age, Gender, hepatitis B) score is based on decade of age(16-29=0, 30-39=2, 40-49=4, 50-59=6, 60-69=8, ≥70=10), gender(M=6, F=0) and platelet count (≥200,000/ll=0, 100,000-199,999/ll=1, <100,000/ll=2): a total sum of ≤9 is considered at low risk of HCC(almost 0% HCC at five years) a score of 10-17 at intermediate risk(3% incidence HCC at five years) and ≥18 is at high risk(17% HCC at five years).
c Endemic country as defined by AASLD hepatitis B virus guidance.
d Surveillance can be initiated as early as third decade of life given median age 46 years at HCC diagnosis.
e Other risk calculators can be considered, although PAGE-B has been validated in Western populations on antiviral therapy.
EASL: European Association for the Study of the Liver; AASLD: American Association for the Study of Liver Diseases

References

1. Chen Z, Chen J, Collins R, Guo Y, Peto R, Wu F, Li L: China Kadoorie Biobank of 0.5 million people: survey methods, baseline characteristics and long-term follow-up. Int J Epidemiol 2011, 40(6):1652-1666.
2. Yang G, Rao C, Ma J, et al. Validation of verbal autopsy procedures for adult deaths in China. Int J Epidemiol. 2006;35(3):741-748.
3. Im PK, Wright N, Yang L, Chan KH, Chen Y, Guo Y, et al. Alcohol consumption and risks of more than 200 diseases in Chinese men. Nat Med. 2023;29(6):1476-86.
4. Im PK, Millwood IY, Kartsonaki C, Guo Y, Chen Y, Turnbull I, et al. Alcohol drinking and risks of liver cancer and non-neoplastic chronic liver diseases in China: a 10-year prospective study of 0.5 million adults. BMC Med. 2021;19(1):216.
5. Pang Y, Kartsonaki C, Guo Y, Chen Y, Yang L, Bian Z, et al. Central adiposity in relation to risk of liver cancer in Chinese adults: A prospective study of 0.5 million people. Int J Cancer. 2019;145(5):1245-53.
6. Pang Y, Kartsonaki C, Turnbull I, Guo Y, Clarke R, Chen Y, et al. Diabetes, Plasma Glucose, and Incidence of Fatty Liver, Cirrhosis, and Liver Cancer: A Prospective Study of 0.5 Million People. Hepatology. 2018;68(4):1308-18.
7. Chan KH, Wright N, Xiao D, Guo Y, Chen Y, Du H, et al. Tobacco smoking and risks of more than 470 diseases in China: a prospective cohort study. Lancet Public Health. 2022;7(12):e1014-e26.
8. EASL Clinical Practice Guidelines: Management of hepatocellular carcinoma. J Hepatol. 2018;69(1):182-236.
9. Singal AG, Llovet JM, Yarchoan M, Mehta N, Heimbach JK, Dawson LA, et al. AASLD practice guidance on prevention, diagnosis, and treatment of hepatocellular carcinoma. Hepatology. 2023.
10. [Guideline for stratified screening and surveillance of primary liver cancer(2020 Edition)]. Zhonghua Gan Zang Bing Za Zhi. 2021;29(1):25-40.

Fig. S1 Calibration plots of 5-year HCC risk prediction models in the CKB

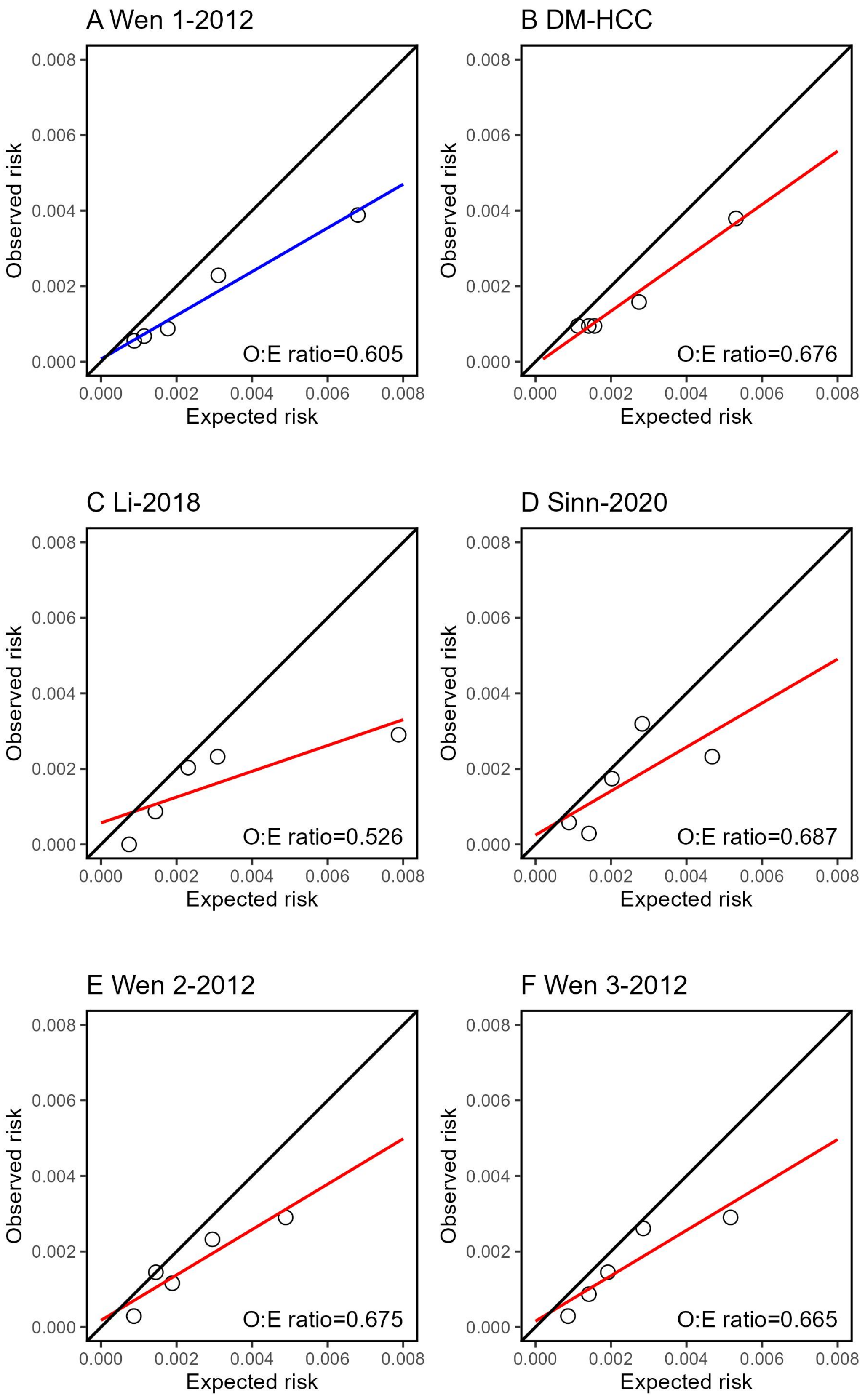

Blue color represents models with non-lab predictors, while red color indicates models that include lab predictors.

Fig. S2 Calibration plots of 5-year CLD risk prediction models in the CKB

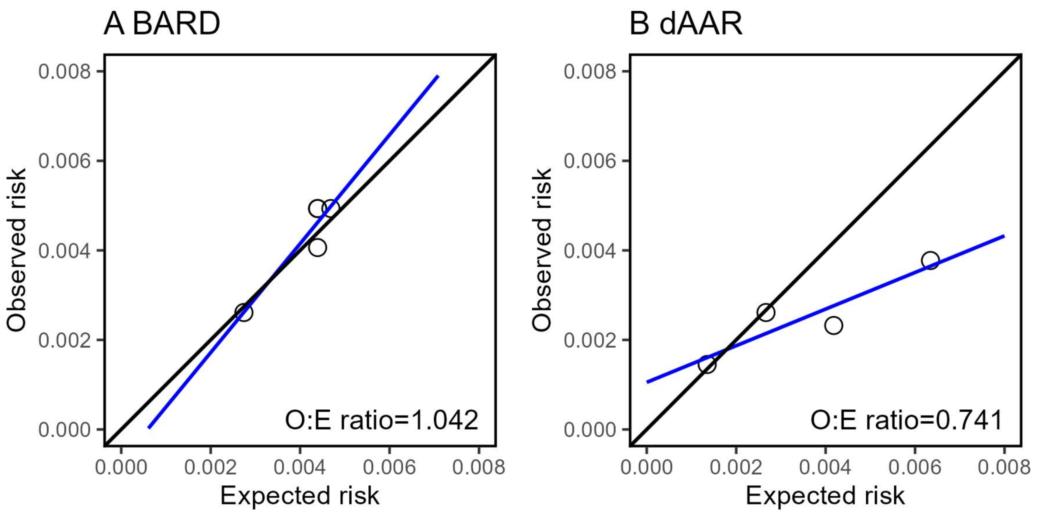

Fig. S3 Discrimination of CLD risk prediction models in the published literature and CKB

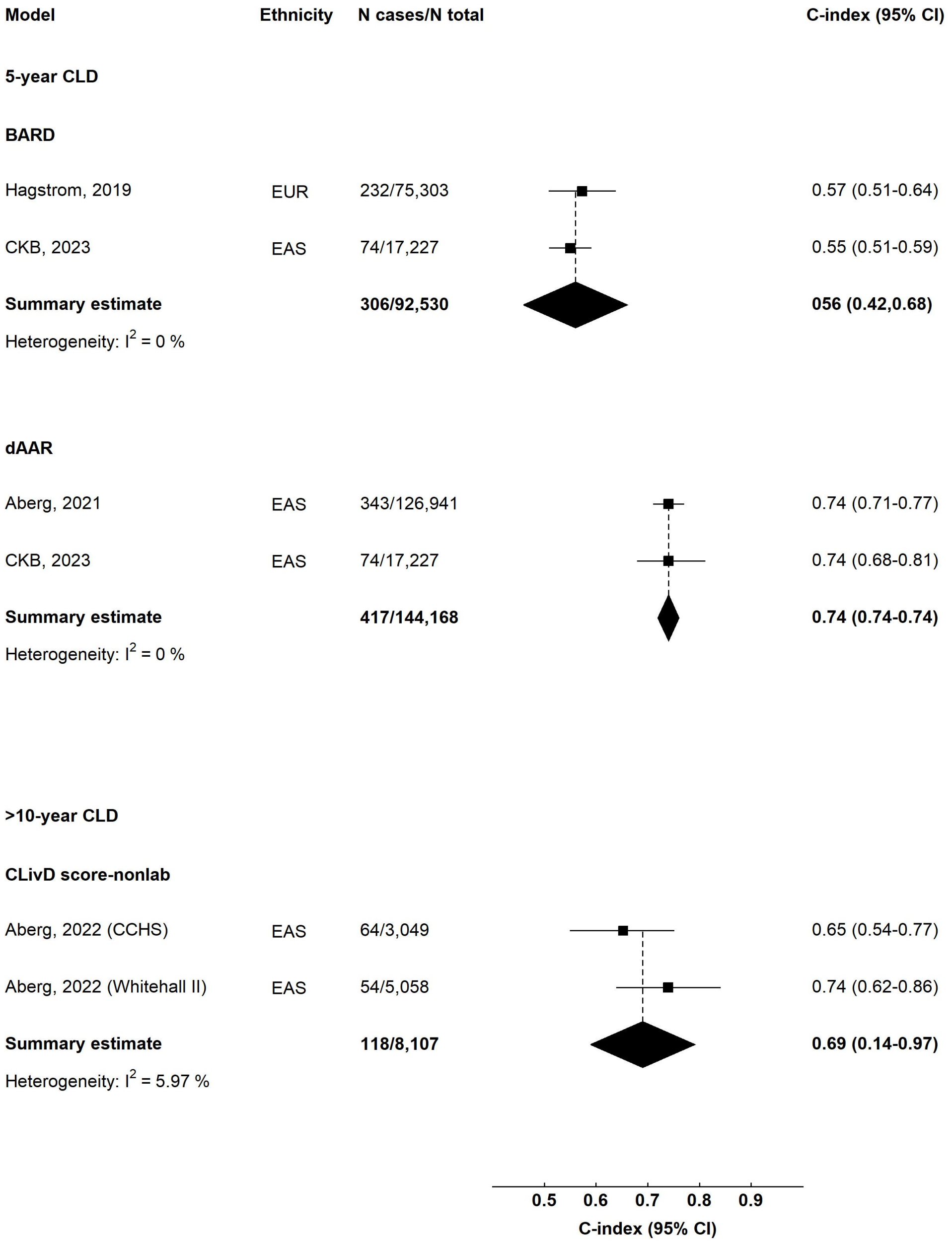

Fig. S4 Discrimination of HCC risk prediction models in the general population

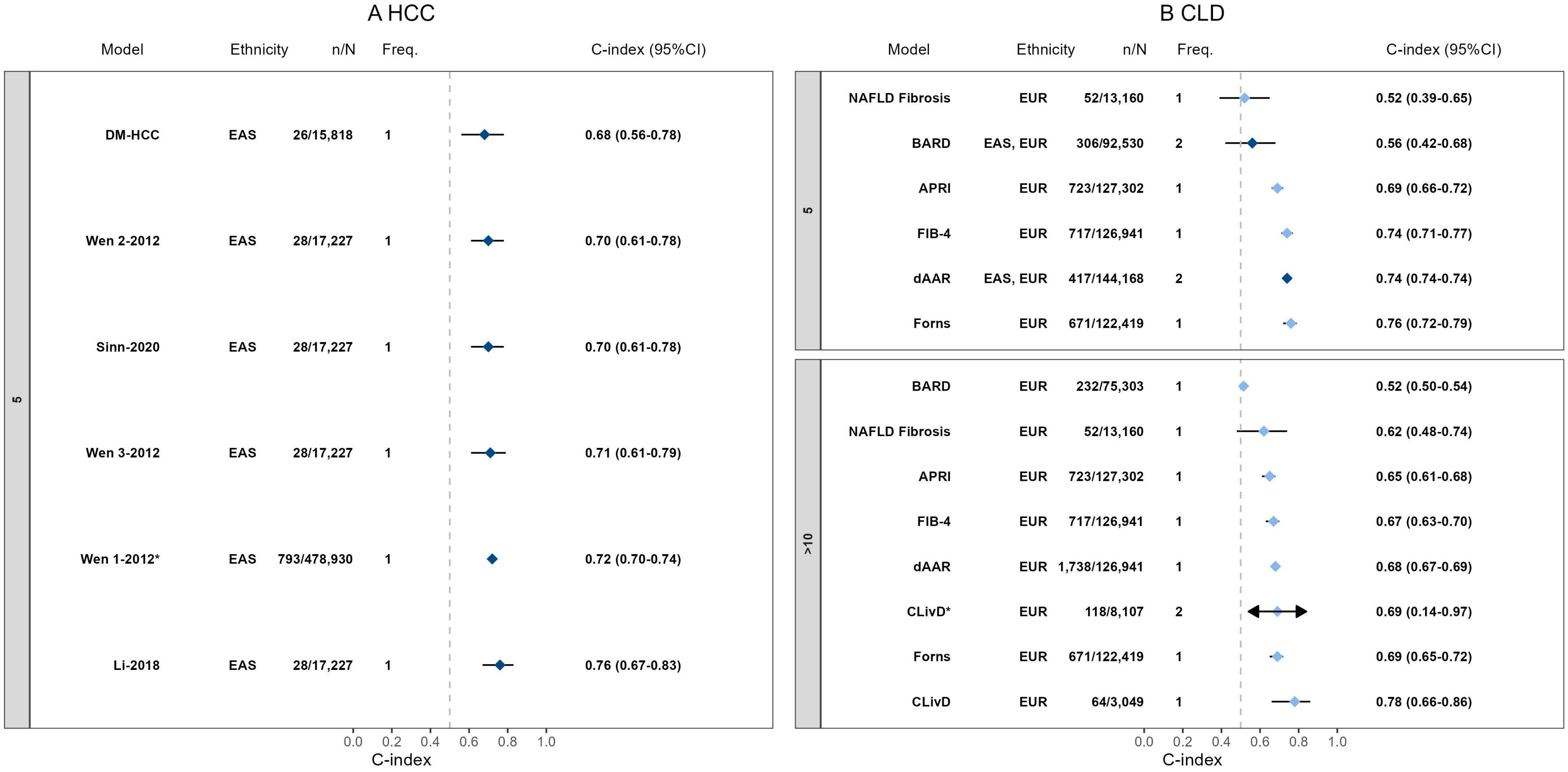

An asterisk (*) represents a predictive model comprising all non-lab predictors. Dark blue color denotes models externally validated in CKB, while light blue color represents models not externally validated in CKB. "Ethnicity" represents the development population, and "Freq." represents the model's external validation frequency. The REAL-B model was not included due to the population being post-treatment or intervention liver disease patients.

Fig. S5 Discrimination of HCC risk prediction models in the HBV infected individuals

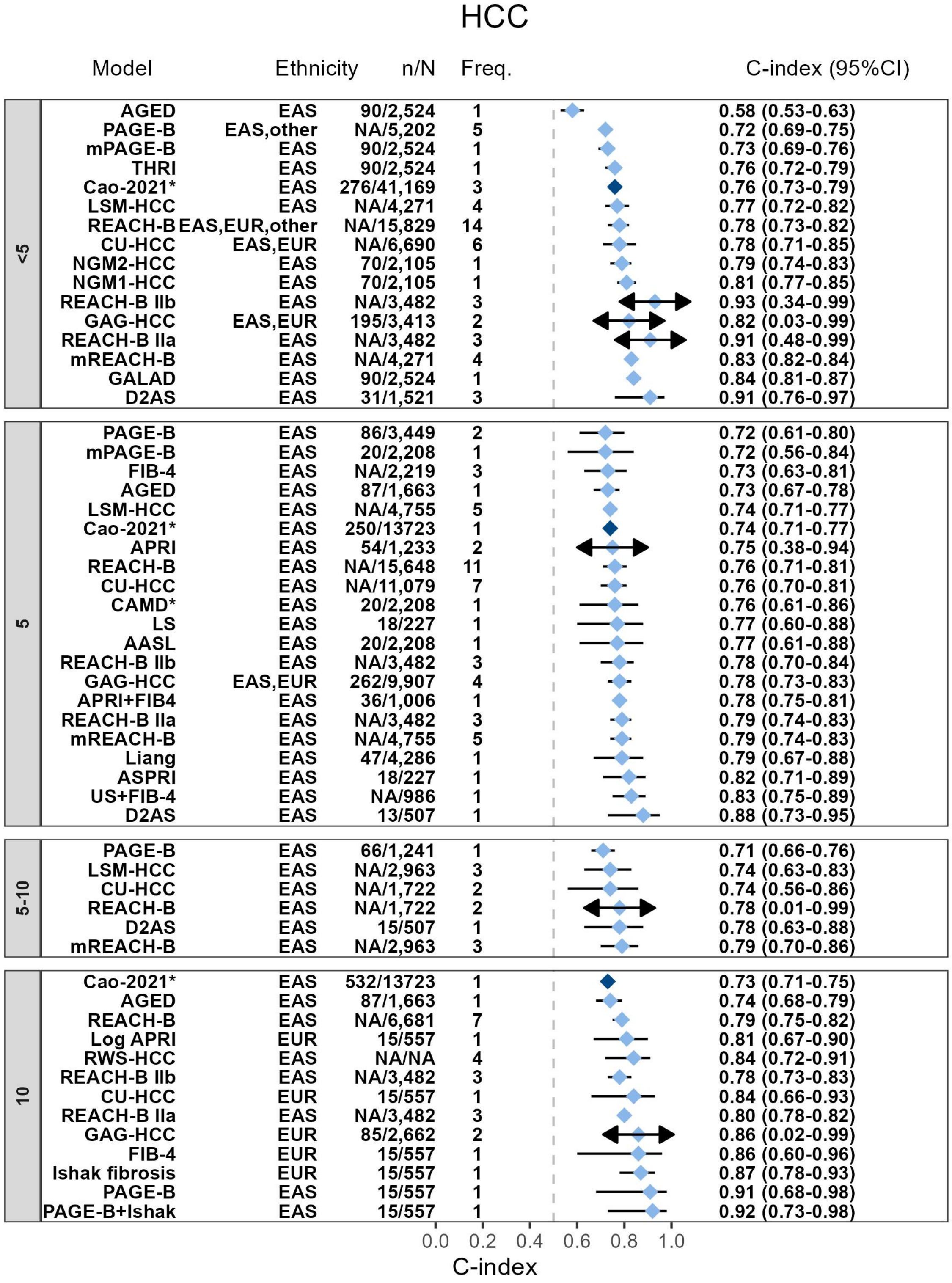

An asterisk (*) represents a predictive model comprising all non-lab predictors. Dark blue color denotes models externally validated in CKB, while light blue color represents models not externally validated in CKB. "Ethnicity" represents the development population, and "Freq." represents the model's external validation frequency. The REAL-B model was not included due to the population being post-treatment or intervention liver disease patients.

Fig. S6 Discrimination of CLD risk prediction models in the HBV infected individuals

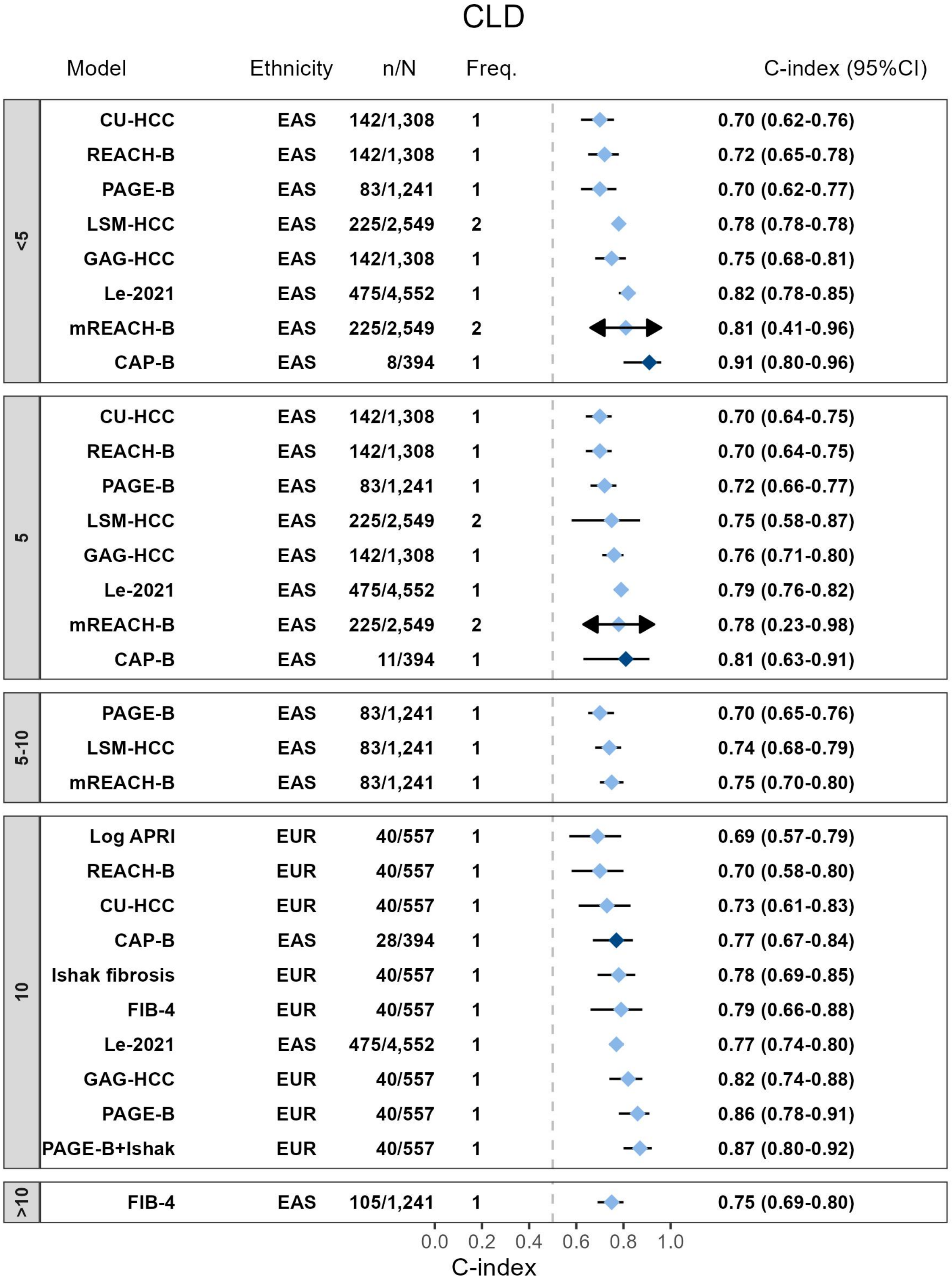

An asterisk (*) represents a predictive model comprising all non-lab predictors. Dark blue color denotes models externally validated in CKB, while light blue color represents models not externally validated in CKB. "Ethnicity" represents the development population, and "Freq." represents the model's external validation frequency. The REAL-B model was not included due to the population being post-treatment or intervention liver disease patients.

Fig. S7 Discrimination of CLD risk prediction models in the HCV infected individuals

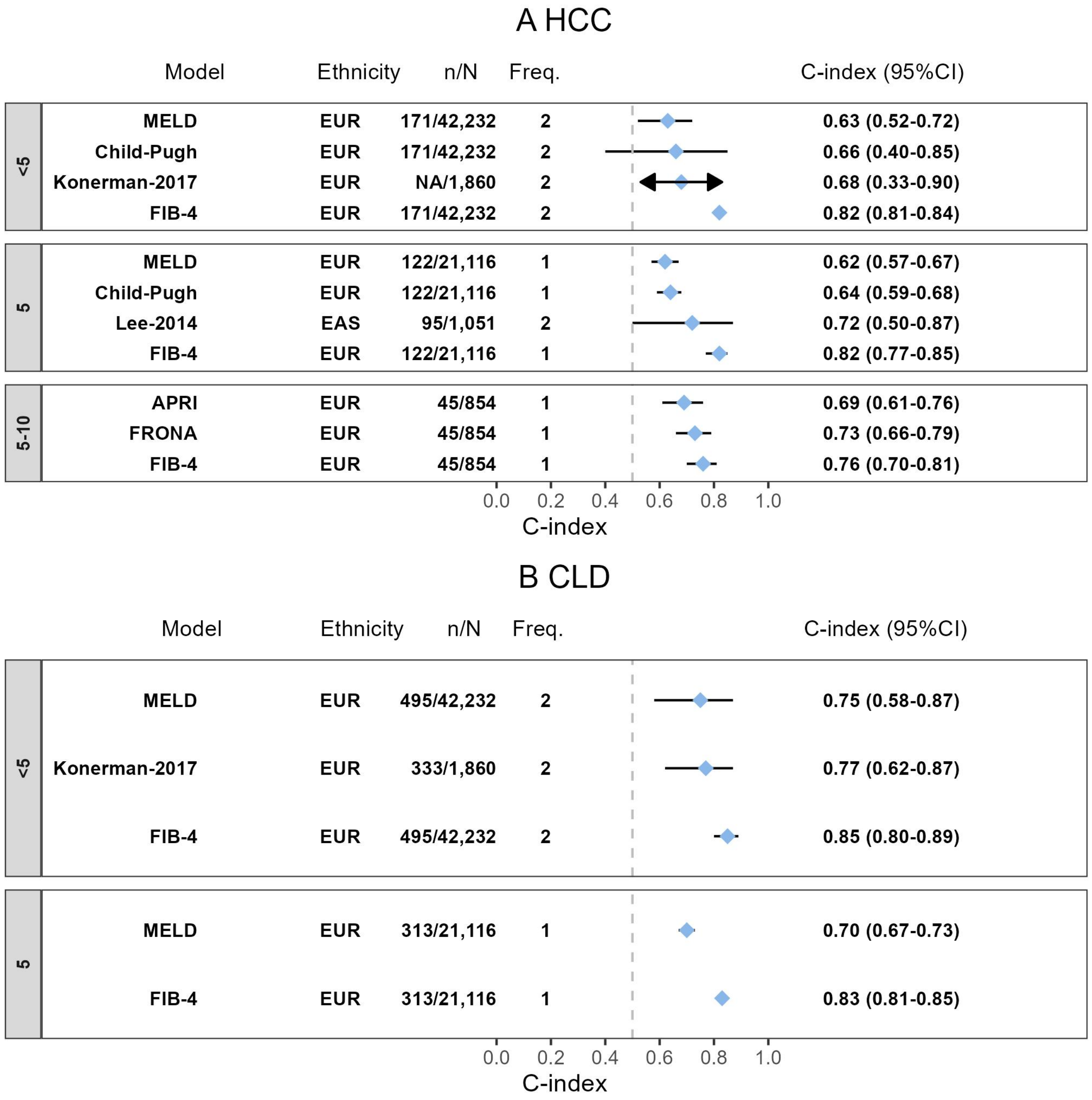

An asterisk (*) represents a predictive model comprising all non-lab predictors. Dark blue color denotes models externally validated in CKB, while light blue color represents models not externally validated in CKB. "Ethnicity" represents the development population, and "Freq." represents the model's external validation frequency. The REAL-B model was not included due to the population being post-treatment or intervention liver disease patients.

Fig. S8 Discrimination of CLD risk prediction models in the NAFLD patients

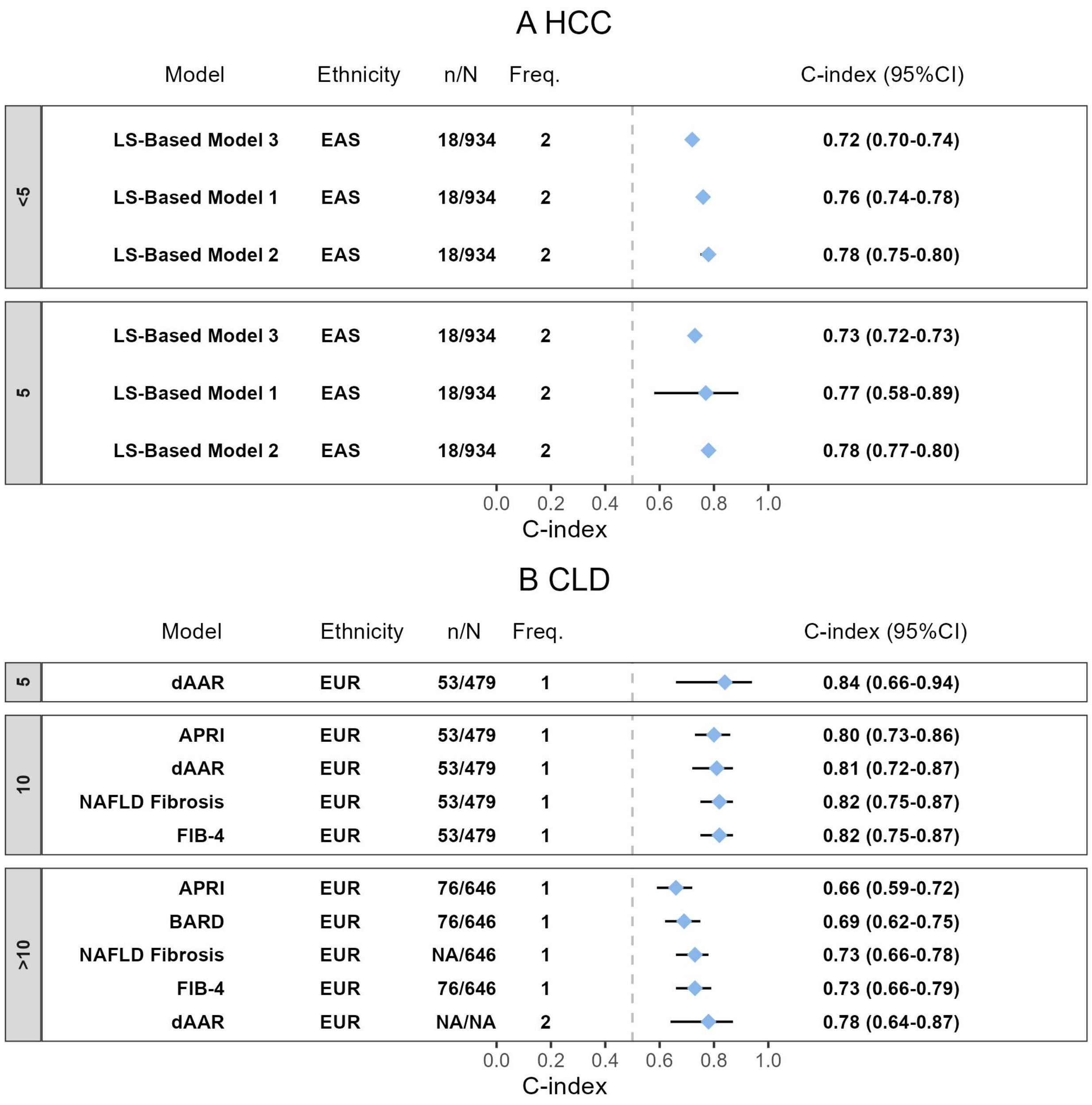

An asterisk (*) represents a predictive model comprising all non-lab predictors. Dark blue color denotes models externally validated in CKB, while light blue color represents models not externally validated in CKB. "Ethnicity" represents the development population, and "Freq." represents the model's external validation frequency. The REAL-B model was not included due to the population being post-treatment or intervention liver disease patients.
