# Supplementary material for: Comparison of models to predict incident chronic liver disease: a systematic review and external validation in Chinese adults
Source: BMC Med. 2024 Dec 31;22:601. doi: 10.1186/s12916-024-03754-9 (PMC11686935; doi:10.1186/s12916-024-03754-9)
Supplement: Supplementary file 2 — Additional file 2. [file 12916_2024_3754_MOESM2_ESM.pdf]

DOI	Model	Objectives	Type of validation	Population	Start of accrual (year)	End of accrual (year)	End of follow-up (year)	Follow-up (average)	Participant type	Location of centres	Outcome (year-disease)	Parameters
10.1016/j.jhep.2022.02.021	CLivD score（non-lab）	D	-	FINRISK and Health 2000	1992	2012	2016	12.9 years	general people	Finland	>10-SLD/advanced liver disease/cirrhosis/liver death	age, WHR, alcohol use (spline variable), diabetes, smoking status, sex*smoking status
	CLivD score（non-lab）	V	Internal	FINRISK and Health 2000	1992	2012	2016	12.9 years	general people	Finland	>10-SLD/advanced liver disease/cirrhosis/liver death	age, WHR, alcohol use (spline variable), diabetes, smoking status, sex*smoking status
	CLivD score（lab）	D	-	FINRISK and Health 2000	1992	2012	2016	12.9 years	general people	Finland	>10-SLD/advanced liver disease/cirrhosis/liver death	age, WHR, alcohol use (spline variable), GGT, diabetes, smoking status, sex*GGT, sex*smoking status
	CLivD score（lab）	V	Internal	FINRISK and Health 2000	1992	2012	2016	12.9 years	general people	Finland	>10-SLD/advanced liver disease/cirrhosis/liver death	age, WHR, alcohol use (spline variable), GGT, diabetes, smoking status, sex*GGT, sex*smoking status
	CLivD score（non-lab）	V	External	Whitehall II	1997	1999	-	21.6 years	general people	UK	>10-SLD/advanced liver disease/cirrhosis/liver death	age, WHR, alcohol use (spline variable), diabetes, smoking status, sex*smoking status
	CLivD score（non-lab）	V	External	CCHS cohorts	2001	2002	-	16.0 years	general people	Denmark	>10-SLD/advanced liver disease/cirrhosis/liver death	age, WHR, alcohol use (spline variable), diabetes, smoking status, sex*smoking status
	CLivD score（lab）	V	External	CCHS cohorts	2001	2002	-	16.0 years	general people	Denmark	>10-SLD/advanced liver disease/cirrhosis/liver death	age, WHR, alcohol use (spline variable), GGT, diabetes, smoking status, sex*GGT, sex*smoking status
	CLivD score（non-lab）	D	-	FINRISK and Health 2000	1992	2012	2016	12.9 years	general people	Finland	>10-SLD/advanced liver disease/cirrhosis/liver death	age, WHR, alcohol use (spline variable), diabetes, smoking status, sex*smoking status
	CLivD score（lab）	D	-	FINRISK and Health 2000	1992	2012	2016	12.9 years	general people	Finland	>10-SLD/advanced liver disease/cirrhosis/liver death	age, WHR, alcohol use (spline variable), GGT, diabetes, smoking status, sex*GGT, sex*smoking status
	CLivD score（non-lab）	D	-	FINRISK and Health 2000	1992	2012	2016	12.9 years	general people	Finland	>10-SLD/advanced liver disease/cirrhosis/liver death	age, WHR, alcohol use (spline variable), diabetes, smoking status, sex*smoking status
	CLivD score（lab）	D	-	FINRISK and Health 2000	1992	2012	2016	12.9 years	general people	Finland	>10-SLD/advanced liver disease/cirrhosis/liver death	age, WHR, alcohol use (spline variable), GGT, diabetes, smoking status, sex*GGT, sex*smoking status
	FIB-4	V	External	FINRISK	1992	2012	2016	12.9 years	general people	Finland	>10-SLD/advanced liver disease/cirrhosis/liver death	age, AST, ALT, and PLT
	APRI	V	External	FINRISK	1992	2012	2016	12.9 years	general people	Finland	>10-SLD/advanced liver disease/cirrhosis/liver death	AST, platelet count
10.1200/jco.2009.26.2675	Won-2010	D	-	Hepatology Clinic at Prince of Wales Hospital (Hong Kong, China), cohort	1997	2000	-	9.94 years	HBV infected	Hong Kang	10-HCC/PLC/liver cancer	age, albumin, bilirubin, HBV DNA, cirrhosis
	Won-2010	V	Internal	Hepatology Clinic at Prince of Wales Hospital (Hong Kong, China),cohort 2	1997	2000	-	10.53 years	HBV infected	Hong Kang	5-HCC/PLC/liver cancer	age, albumin, bilirubin, HBV DNA, cirrhosis
	Won-2010	V	Internal	Hepatology Clinic at Prince of Wales Hospital (Hong Kong, China),cohort 2	1997	2000	-	10.53 years	HBV infected	Hong Kang	10-HCC/PLC/liver cancer	age, albumin, bilirubin, HBV DNA, cirrhosis
	Won-2010	V	Internal	Hepatology Clinic at Prince of Wales Hospital (Hong Kong, China),cohort 2	1997	2000	-	10.53 years	HBV infected	Hong Kang	10-HCC/PLC/liver cancer	age, albumin, bilirubin, HBV DNA, cirrhosis
10.1200/jco.2009.27.4456	Model 1- regression model	D	-	REVEAL-HBV	1991	1992	-	-	HBV infected	Taiwan	5-HCC/PLC/liver cancer	sex, age in 5-year increments, family history of hepatocellular carcinoma, and serum ALT level, HBeAg serostatus
	Model 1- regression model	V	Internal	REVEAL-HBV	1991	1992	-	-	HBV infected	Taiwan	5-HCC/PLC/liver cancer	sex, age in 5-year increments, family history of hepatocellular carcinoma, and serum ALT level, HBeAg serostatus
	Model 1- regression model	D	-	REVEAL-HBV	1991	1992	-	-	HBV infected	Taiwan	10-HCC/PLC/liver cancer	sex, age in 5-year increments, family history of hepatocellular carcinoma, and serum ALT level, HBeAg serostatus
	Model 1- regression model	V	Internal	REVEAL-HBV	1991	1992	-	-	HBV infected	Taiwan	10-HCC/PLC/liver cancer	sex, age in 5-year increments, family history of hepatocellular carcinoma, and serum ALT level, HBeAg serostatus
	Model 2-regression model	D	-	REVEAL-HBV	1991	1992	-	-	HBV infected	Taiwan	5-HCC/PLC/liver cancer	sex, age in 5-year increments, family history of hepatocellular carcinoma, and serum ALT level, a combined variable comprising HBeAg and serum HBV DNA level
	Model 2-regression model	V	Internal	REVEAL-HBV	1991	1992	-	-	HBV infected	Taiwan	5-HCC/PLC/liver cancer	sex, age in 5-year increments, family history of hepatocellular carcinoma, and serum ALT level, a combined variable comprising HBeAg and serum HBV DNA level
	Model 2-regression model	D	-	REVEAL-HBV	1991	1992	-	-	HBV infected	Taiwan	10-HCC/PLC/liver cancer	sex, age in 5-year increments, family history of hepatocellular carcinoma, and serum ALT level, a combined variable comprising HBeAg and serum HBV DNA level
	Model 2-regression model	V	Internal	REVEAL-HBV	1991	1992	-	-	HBV infected	Taiwan	10-HCC/PLC/liver cancer	sex, age in 5-year increments, family history of hepatocellular carcinoma, and serum ALT level, a combined variable comprising HBeAg and serum HBV DNA level
	Model 3-regression model	D	-	REVEAL-HBV	1991	1992	-	-	HBV infected	Taiwan	5-HCC/PLC/liver cancer	sex, age in 5-year increments, family history of hepatocellular carcinoma, and serum ALT level, a combined variable consisting of HBeAg, serum HBV DNA level, and HBV genotype
	Model 3-regression model	V	Internal	REVEAL-HBV	1991	1992	-	-	HBV infected	Taiwan	5-HCC/PLC/liver cancer	sex, age in 5-year increments, family history of hepatocellular carcinoma, and serum ALT level, a combined variable consisting of HBeAg, serum HBV DNA level, and HBV genotype
	Model 3-regression model	D	-	REVEAL-HBV	1991	1992	-	-	HBV infected	Taiwan	10-HCC/PLC/liver cancer	sex, age in 5-year increments, family history of hepatocellular carcinoma, and serum ALT level, a combined variable consisting of HBeAg, serum HBV DNA level, and HBV genotype
	Model 3-regression model	V	Internal	REVEAL-HBV	1991	1992	-	-	HBV infected	Taiwan	10-HCC/PLC/liver cancer	sex, age in 5-year increments, family history of hepatocellular carcinoma, and serum ALT level, a combined variable consisting of HBeAg, serum HBV DNA level, and HBV genotype
	NGM1-HCC	D	-	REVEAL-HBV	1991	1992	-	-	HBV infected	Taiwan	5-HCC/PLC/liver cancer	sex, age in 5-year increments, family history of hepatocellular carcinoma, and serum ALT level, HBeAg serostatus
	NGM1-HCC	V	Internal	REVEAL-HBV	1991	1992	-	-	HBV infected	Taiwan	5-HCC/PLC/liver cancer	sex, age in 5-year increments, family history of hepatocellular carcinoma, and serum ALT level, HBeAg serostatus
	NGM1-HCC	D	-	REVEAL-HBV	1991	1992	-	-	HBV infected	Taiwan	10-HCC/PLC/liver cancer	sex, age in 5-year increments, family history of hepatocellular carcinoma, and serum ALT level, HBeAg serostatus

	NGM1-HCC	V	Internal	REVEAL-HBV	1991	1992	-	-	HBV infected	Taiwan	10-HCC/PLC/liver cancer	sex, age in 5-year increments, family history of hepatocellular carcinoma, and serum ALT level, HBeAg serostatus
	NGM2-HCC	D	-	REVEAL-HBV	1991	1992	-	-	HBV infected	Taiwan	5-HCC/PLC/liver cancer	sex, age in 5-year increments, family history of hepatocellular carcinoma, and serum ALT level, a combined variable comprising HBeAg and serum HBV DNA level
	NGM2-HCC	V	Internal	REVEAL-HBV	1991	1992	-	-	HBV infected	Taiwan	5-HCC/PLC/liver cancer	sex, age in 5-year increments, family history of hepatocellular carcinoma, and serum ALT level, a combined variable comprising HBeAg and serum HBV DNA level
	NGM2-HCC	D	-	REVEAL-HBV	1991	1992	-	-	HBV infected	Taiwan	10-HCC/PLC/liver cancer	sex, age in 5-year increments, family history of hepatocellular carcinoma, and serum ALT level, a combined variable comprising HBeAg and serum HBV DNA level
	NGM2-HCC	V	Internal	REVEAL-HBV	1991	1992	-	-	HBV infected	Taiwan	10-HCC/PLC/liver cancer	sex, age in 5-year increments, family history of hepatocellular carcinoma, and serum ALT level, a combined variable comprising HBeAg and serum HBV DNA level
	NGM3-HCC	D	-	REVEAL-HBV	1991	1992	-	-	HBV infected	Taiwan	5-HCC/PLC/liver cancer	sex, age in 5-year increments, family history of hepatocellular carcinoma, and serum ALT level, a combined variable consisting of HBeAg, serum HBV DNA level, and HBV genotype
	NGM3-HCC	V	Internal	REVEAL-HBV	1991	1992	-	-	HBV infected	Taiwan	5-HCC/PLC/liver cancer	sex, age in 5-year increments, family history of hepatocellular carcinoma, and serum ALT level, a combined variable consisting of HBeAg, serum HBV DNA level, and HBV genotype
	NGM3-HCC	D	-	REVEAL-HBV	1991	1992	-	-	HBV infected	Taiwan	10-HCC/PLC/liver cancer	sex, age in 5-year increments, family history of hepatocellular carcinoma, and serum ALT level, a combined variable consisting of HBeAg, serum HBV DNA level, and HBV genotype
	NGM3-HCC	V	Internal	REVEAL-HBV	1991	1992	-	-	HBV infected	Taiwan	10-HCC/PLC/liver cancer	sex, age in 5-year increments, family history of hepatocellular carcinoma, and serum ALT level, a combined variable consisting of HBeAg, serum HBV DNA level, and HBV genotype
10.1016/j.jhep.2008.07.023	GAG-HCC	D	-	Queen Mary Hospital, Hong Kong,	1995	2005	-	76.8 months	HBV infected	Hong Kang	5-HCC/PLC/liver cancer	sex, age, HBV DNA levels in copies/mL in log, cirrhosis, core promoter mutations
	GAG-HCC	D	-	Queen Mary Hospital, Hong Kong,	1995	2005	-	76.8 months	HBV infected	Hong Kang	5-HCC/PLC/liver cancer	sex, age, HBV DNA levels in copies/mL in log, cirrhosis, core promoter mutations
	GAG-HCC(non core promoter mutations)	D	-	Queen Mary Hospital, Hong Kong,	1995	2005	-	76.8 months	HBV infected	Hong Kang	10-HCC/PLC/liver cancer	sex, age, HBV DNA levels in copies/mL in log, cirrhosis
	GAG-HCC(non core promoter mutations)	D	-	Queen Mary Hospital, Hong Kong,	1995	2005	-	76.8 months	HBV infected	Hong Kang	10-HCC/PLC/liver cancer	sex, age, HBV DNA levels in copies/mL in log, cirrhosis
10.1016/s1470-2045(11)70077-8	REACH-B	D	-	REVEAL-HBV	1991	1992	-	-	HBV infected	Taiwan	<5-HCC/PLC/liver cancer	sex, age, serum ALT concentration, HBeAg status, serum HBV DNA level (by PCR assay)
	REACH-B	D	-	REVEAL-HBV	1991	1992	-	-	HBV infected	Taiwan	5-HCC/PLC/liver cancer	sex, age, serum ALT concentration, HBeAg status, serum HBV DNA level (by PCR assay)
	REACH-B	D	-	REVEAL-HBV	1991	1992	-	-	HBV infected	Taiwan	10-HCC/PLC/liver cancer	sex, age, serum ALT concentration, HBeAg status, serum HBV DNA level (by PCR assay)
	REACH-B	V	External	hospital-based composite international cohort	-	-	-	-	HBV infected	Hong Kong, Yonsei	<5-HCC/PLC/liver cancer	sex, age, serum ALT concentration, HBeAg status, serum HBV DNA level (by PCR assay)
	REACH-B	V	External	hospital-based composite international cohort	-	-	-	-	HBV infected	Hong Kong, Yonsei	5-HCC/PLC/liver cancer	sex, age, serum ALT concentration, HBeAg status, serum HBV DNA level (by PCR assay)
	REACH-B	V	External	hospital-based composite international cohort	-	-	-	-	HBV infected	Hong Kong, Yonsei	10-HCC/PLC/liver cancer	sex, age, serum ALT concentration, HBeAg status, serum HBV DNA level (by PCR assay)
	REACH-B	V	External	University of Hong Kong (UHK)	-	-	-	6.3 years	HBV infected	Hong Kang	<5-HCC/PLC/liver cancer	sex, age, serum ALT concentration, HBeAg status, serum HBV DNA level (by PCR assay)
	REACH-B	V	External	University of Hong Kong (UHK)	-	-	-	6.3 years	HBV infected	Hong Kang	5-HCC/PLC/liver cancer	sex, age, serum ALT concentration, HBeAg status, serum HBV DNA level (by PCR assay)
	REACH-B	V	External	University of Hong Kong (UHK)	-	-	-	6.3 years	HBV infected	Hong Kang	10-HCC/PLC/liver cancer	sex, age, serum ALT concentration, HBeAg status, serum HBV DNA level (by PCR assay)
	REACH-B	V	External	Chinese University of Hong Kong (CUHK)	-	-	-	9.4 years	HBV infected	Hong Kang	<5-HCC/PLC/liver cancer	sex, age, serum ALT concentration, HBeAg status, serum HBV DNA level (by PCR assay)
	REACH-B	V	External	Chinese University of Hong Kong (CUHK)	-	-	-	9.4 years	HBV infected	Hong Kang	5-HCC/PLC/liver cancer	sex, age, serum ALT concentration, HBeAg status, serum HBV DNA level (by PCR assay)
	REACH-B	V	External	Chinese University of Hong Kong (CUHK)	-	-	-	9.4 years	HBV infected	Hong Kang	10-HCC/PLC/liver cancer	sex, age, serum ALT concentration, HBeAg status, serum HBV DNA level (by PCR assay)
	REACH-B	V	External	Yonsei University Hospital (YUH)	-	-	-	7.0 years	HBV infected	Yonsei	<5-HCC/PLC/liver cancer	sex, age, serum ALT concentration, HBeAg status, serum HBV DNA level (by PCR assay)
	REACH-B	V	External	Yonsei University Hospital (YUH)	-	-	-	7.0 years	HBV infected	Yonsei	5-HCC/PLC/liver cancer	sex, age, serum ALT concentration, HBeAg status, serum HBV DNA level (by PCR assay)
	REACH-B	V	External	Yonsei University Hospital (YUH)	-	-	-	7.0 years	HBV infected	Yonsei	10-HCC/PLC/liver cancer	sex, age, serum ALT concentration, HBeAg status, serum HBV DNA level (by PCR assay)
10.1016/j.jhep.2011.09.011	Kurosaki-2012	D	-	Musashino Red Cross Hospital	-	-	-	-	HCV infected	Japan	5-HCC/PLC/liver cancer	age, albumin levels,platelet count
	Kurosaki-2012	V	External	patients in University of Yamanashi, Tokyo Medical and Dental University, Osaka University, Osaka City University, Nagoya City University, or Toranomon Hospital.	-	-	-	-	HCV infected	Japan	5-HCC/PLC/liver cancer	age, albumin levels,platelet count

10.1016/j.ypmed.2012.05.017	**Kurosaki-2012**	**D**	-	JPHC Study Cohort II	1993	1994	2006	12.6 years	general people	Japan	10-HCC/PLC/liver cancer	age, sex, alcohol consumption, BMI, diabetes, coffee consumption, HBV/HCV infection status.
	**Kurosaki-2012**	**V**	**Internal**	JPHC Study Cohort II	1993	1994	2006	12.6 years	general people	Japan	10-HCC/PLC/liver cancer	age, sex, alcohol consumption, BMI, diabetes, coffee consumption, HBV/HCV infection status.
10.1093/jnci/djs372	**Wen 1-2012**	**D**	-	MJ cohort	1994	-	2008	8.5 years	general people	Taiwan	10-HCC/PLC/liver cancer	age, sex, pack-year of smoking, alcohol drinking, physical activity, diabetes
	**Wen 2-2012**	**D**	-	MJ cohort	1994	-	2008	8.5 years	general people	Taiwan	10-HCC/PLC/liver cancer	age, sex, AST, ALT
	**Wen 3-2012**	**D**	-	MJ cohort	1994	-	2008	8.5 years	general people	Taiwan	10-HCC/PLC/liver cancer	age, sex, pack-year of smoking, alcohol drinking, physical activity, diabetes, AST, and ALT
	**Wen 4-2012**	**D**	-	MJ cohort	1994	-	2008	8.5 years	general people	Taiwan	10-HCC/PLC/liver cancer	age, sex, pack-year of smoking, alcohol drinking, physical activity, diabetes, AST, ALT, AFP, and HBV
	**Wen 1-2012**	**V**	**Internal**	MJ cohort	1994	-	2008	8.5 years	general people	Taiwan	10-HCC/PLC/liver cancer	age, sex, pack-year of smoking, alcohol drinking, physical activity, diabetes
	**Wen 2-2012**	**V**	**Internal**	MJ cohort	1994	-	2008	8.5 years	general people	Taiwan	10-HCC/PLC/liver cancer	age, sex, AST, ALT
	**Wen 3-2012**	**V**	**Internal**	MJ cohort	1994	-	2008	8.5 years	general people	Taiwan	10-HCC/PLC/liver cancer	age, sex, pack-year of smoking, alcohol drinking, physical activity, diabetes, AST, and ALT
	**Wen 4-2012**	**V**	**Internal**	MJ cohort	1994	-	2008	8.5 years	general people	Taiwan	10-HCC/PLC/liver cancer	age, sex, pack-year of smoking, alcohol drinking, physical activity, diabetes, AST, ALT, AFP, and HBV
	**Wen 1-2012**	**D+V**	-	MJ cohort	1994	-	2008	8.5 years	general people	Taiwan	10-HCC/PLC/liver cancer	age, sex, pack-year of smoking, alcohol drinking, physical activity, diabetes
	**Wen 2-2012**	**D+V**	-	MJ cohort	1994	-	2008	8.5 years	general people	Taiwan	10-HCC/PLC/liver cancer	age, sex, AST, ALT
	**Wen 3-2012**	**D+V**	-	MJ cohort	1994	-	2008	8.5 years	general people	Taiwan	10-HCC/PLC/liver cancer	age, sex, pack-year of smoking, alcohol drinking, physical activity, diabetes, AST, and ALT
	**Wen 4-2012**	**D+V**	-	MJ cohort	1994	-	2008	8.5 years	general people	Taiwan	10-HCC/PLC/liver cancer	age, sex, pack-year of smoking, alcohol drinking, physical activity, diabetes, AST, ALT, AFP, and HBV
	**Wen 1'-2012**	**D**	-	MJ cohort	1994	-	2008	8.5 years	general people	Taiwan	10-HCC/PLC/liver cancer	age, sex, pack-year of smoking, alcohol drinking, physical activity, diabetes
	**Wen 2'-2012**	**D**	-	MJ cohort	1994	-	2008	8.5 years	general people	Taiwan	10-HCC/PLC/liver cancer	age, sex, AST, ALT
	**Wen 3'-2012**	**D**	-	MJ cohort	1994	-	2008	8.5 years	general people	Taiwan	10-HCC/PLC/liver cancer	age, sex, pack-year of smoking, alcohol drinking, physical activity, diabetes, AST, and ALT
	**Wen 4'-2012**	**D**	-	MJ cohort	1994	-	2008	8.5 years	general people	Taiwan	10-HCC/PLC/liver cancer	age, sex, pack-year of smoking, alcohol drinking, physical activity, diabetes, AST, ALT, AFP, and HBV
	**Wen 5'-2012**	**D**	-	MJ cohort	1994	-	2008	8.5 years	general people	Taiwan	10-HCC/PLC/liver cancer	age, sex, pack-year of smoking, alcohol drinking, physical activity, diabetes, AST, ALT, AFP, HBV and HCV
	**Wen 1'-2012**	**V**	**Internal**	MJ cohort	1994	-	2008	8.5 years	general people	Taiwan	10-HCC/PLC/liver cancer	age, sex, pack-year of smoking, alcohol drinking, physical activity, diabetes
	**Wen 2'-2012**	**V**	**Internal**	MJ cohort	1994	-	2008	8.5 years	general people	Taiwan	10-HCC/PLC/liver cancer	age, sex, AST, ALT
	**Wen 3'-2012**	**V**	**Internal**	MJ cohort	1994	-	2008	8.5 years	general people	Taiwan	10-HCC/PLC/liver cancer	age, sex, pack-year of smoking, alcohol drinking, physical activity, diabetes, AST, and ALT
	**Wen 4'-2012**	**V**	**Internal**	MJ cohort	1994	-	2008	8.5 years	general people	Taiwan	10-HCC/PLC/liver cancer	age, sex, pack-year of smoking, alcohol drinking, physical activity, diabetes, AST, ALT, AFP, and HBV
	**Wen 5'-2012**	**V**	**Internal**	MJ cohort	1994	-	2008	8.5 years	general people	Taiwan	10-HCC/PLC/liver cancer	age, sex, pack-year of smoking, alcohol drinking, physical activity, diabetes, AST, ALT, AFP, HBV and HCV
	**Wen 1'-2012**	**D+V**	-	MJ cohort	1994	-	2008	8.5 years	general people	Taiwan	10-HCC/PLC/liver cancer	age, sex, pack-year of smoking, alcohol drinking, physical activity, diabetes
	**Wen 2'-2012**	**D+V**	-	MJ cohort	1994	-	2008	8.5 years	general people	Taiwan	10-HCC/PLC/liver cancer	age, sex, AST, ALT
	**Wen 3'-2012**	**D+V**	-	MJ cohort	1994	-	2008	8.5 years	general people	Taiwan	10-HCC/PLC/liver cancer	age, sex, pack-year of smoking, alcohol drinking, physical activity, diabetes, AST, and ALT
	**Wen 4'-2012**	**D+V**	-	MJ cohort	1994	-	2008	8.5 years	general people	Taiwan	10-HCC/PLC/liver cancer	age, sex, pack-year of smoking, alcohol drinking, physical activity, diabetes, AST, ALT, AFP, and HBV
	**Wen 5'-2012**	**D+V**	-	MJ cohort	1994	-	2008	8.5 years	general people	Taiwan	10-HCC/PLC/liver cancer	age, sex, pack-year of smoking, alcohol drinking, physical activity, diabetes, AST, ALT, AFP, HBV and HCV
10.1016/j.ejim.2013.06.010	**HCC-4 Risk Score**	**D**	-	the hepatology section of the Internal Medicine Department in Hospital Virgen de laVictoria in Málaga, Spain	1993	2010	-	82 months	HCV infected	Spain	5~10-HCC/PLC/liver cancer	age, platelet, gammaglobulin and baseline AFP
	**FRONA**	**V**	**External**	the hepatology section of the Internal Medicine Department in Hospital Virgen de laVictoria in Málaga, Spain	1993	2010	-	82 months	HCV infected	Spain	5~10-HCC/PLC/liver cancer	-
	**APRI**	**V**	**External**	the hepatology section of the Internal Medicine Department in Hospital Virgen de laVictoria in Málaga, Spain	1993	2010	-	82 months	HCV infected	Spain	5~10-HCC/PLC/liver cancer	AST, platelet count
	**FIB-4**	**V**	**External**	the hepatology section of the Internal Medicine Department in Hospital Virgen de laVictoria in Málaga, Spain	1993	2010	-	82 months	HCV infected	Spain	5~10-HCC/PLC/liver cancer	age, AST, ALT, and PLT
	**Lee-2013**	**D**	-	REVEAL-HBV	1991	1992	-	-	HBV infected	Taiwan	<5-SLD/advanced liver disease/cirrhosis/liver death	age, gender, HBVe antigen (HBeAg) serostatus, serum levels of HBV DNA,and alanine aminotransferase (ALT)
	**Lee-2013**	**D**	-	REVEAL-HBV	1991	1992	-	-	HBV infected	Taiwan	5-SLD/advanced liver disease/cirrhosis/liver death	age, gender, HBVe antigen (HBeAg) serostatus, serum levels of HBV DNA,and alanine aminotransferase (ALT)
	**Lee-2013**	**D**	-	REVEAL-HBV	1991	1992	-	-	HBV infected	Taiwan	10-SLD/advanced liver disease/cirrhosis/liver death	age, gender, HBVe antigen (HBeAg) serostatus, serum levels of HBV DNA,and alanine aminotransferase (ALT)
	**Lee-2013**	**V**	**Internal**	REVEAL-HBV	1991	1992	-	-	HBV infected	Taiwan	<5-SLD/advanced liver disease/cirrhosis/liver death	age, gender, HBVe antigen (HBeAg) serostatus, serum levels of HBV DNA,and alanine aminotransferase (ALT)

10.1002/hep.26385	Lee-2013	V	Internal	REVEAL-HBV	1991	1992	-	-	HBV infected	Taiwan	5-SLD/advanced liver disease/cirrhosis/liver death	age, gender, HBVe antigen (HBeAg) serostatus, serum levels of HBV DNA,and alanine aminotransferase (ALT)
	Lee-2013	V	Internal	REVEAL-HBV	1991	1992	-	-	HBV infected	Taiwan	10-SLD/advanced liver disease/cirrhosis/liver death	age, gender, HBVe antigen (HBeAg) serostatus, serum levels of HBV DNA,and alanine aminotransferase (ALT)
	Lee-2013	D	-	REVEAL-HBV	1991	1992	-	-	HBV infected	Taiwan	<5-HCC/PLC/liver cancer	age, gender, HBVe antigen (HBeAg) serostatus, serum levels of HBV DNA,alanine aminotransferase (ALT) and family history
	Lee-2013	D	-	REVEAL-HBV	1991	1992	-	-	HBV infected	Taiwan	5-HCC/PLC/liver cancer	age, gender, HBVe antigen (HBeAg) serostatus, serum levels of HBV DNA,alanine aminotransferase (ALT) and family history
	Lee-2013	D	-	REVEAL-HBV	1991	1992	-	-	HBV infected	Taiwan	10-HCC/PLC/liver cancer	age, gender, HBVe antigen (HBeAg) serostatus, serum levels of HBV DNA,alanine aminotransferase (ALT) and family history
	Lee-2013	V	Internal	REVEAL-HBV	1991	1992	-	-	HBV infected	Taiwan	<5-HCC/PLC/liver cancer	age, gender, HBVe antigen (HBeAg) serostatus, serum levels of HBV DNA,alanine aminotransferase (ALT) and family history
	Lee-2013	V	Internal	REVEAL-HBV	1991	1992	-	-	HBV infected	Taiwan	5-HCC/PLC/liver cancer	age, gender, HBVe antigen (HBeAg) serostatus, serum levels of HBV DNA,alanine aminotransferase (ALT) and family history
	Lee-2013	V	Internal	REVEAL-HBV	1991	1992	-	-	HBV infected	Taiwan	10-HCC/PLC/liver cancer	age, gender, HBVe antigen (HBeAg) serostatus, serum levels of HBV DNA,alanine aminotransferase (ALT) and family history
10.1371/journal.pone.0061448	Lin-Model I	D	-	REVEAL-HBV	1991	1992	2008	5.9 year	HBV infected	Taiwan	5~10-HCC/PLC/liver cancer	age, gender, serum ALT levels, and HBV seromarkers
	Lin-Model II	D	-	REVEAL-HBV	1991	1992	2008	5.9 year	HBV infected	Taiwan	5~10-HCC/PLC/liver cancer	age, gender, and the liver-related seromarkers ALT, AAR, AFP, GGT, albumin, and alpha-1 globulin
	Lin-Model III	D	-	REVEAL-HBV	1991	1992	2008	5.9 year	HBV infected	Taiwan	5~10-HCC/PLC/liver cancer	age, gender, and the liver-related seromarkers ALT, AAR, AFP, GGT, albumin, alpha-1 globulin and HBV seromarkers
10.1371/journal.pone.0094760	Lee-2014	D	-	R.E.V.E.A.L.-HCV Cohort	1991	1992	2008	-	HCV infected	Taiwan	5-HCC/PLC/liver cancer	age, alanine aminotransferase (ALT), the ratio of aspirate aminotransferase to ALT, serum HCV RNA levels and cirrhosis status and HCV genotype
	Lee-2014	D	-	R.E.V.E.A.L.-HCV Cohort	1991	1992	2008	-	HCV infected	Taiwan	10-HCC/PLC/liver cancer	age, alanine aminotransferase (ALT), the ratio of aspirate aminotransferase to ALT, serum HCV RNA levels and cirrhosis status and HCV genotype
	Lee-2014	D	-	R.E.V.E.A.L.-HCV Cohort	1991	1992	2008	-	HCV infected	Taiwan	>10-HCC/PLC/liver cancer	age, alanine aminotransferase (ALT), the ratio of aspirate aminotransferase to ALT, serum HCV RNA levels and cirrhosis status and HCV genotype
	Lee-2014	D	-	R.E.V.E.A.L.-HCV Cohort	1991	1992	2008	-	HCV infected	Taiwan	5-HCC/PLC/liver cancer	age, alanine aminotransferase (ALT), the ratio of aspirate aminotransferase to ALT, serum HCV RNA levels and cirrhosis status and HCV genotype
	Lee-2014	D	-	R.E.V.E.A.L.-HCV Cohort	1991	1992	2008	-	HCV infected	Taiwan	10-HCC/PLC/liver cancer	age, alanine aminotransferase (ALT), the ratio of aspirate aminotransferase to ALT, serum HCV RNA levels and cirrhosis status and HCV genotype
	Lee-2014	D	-	R.E.V.E.A.L.-HCV Cohort	1991	1992	2008	-	HCV infected	Taiwan	>10-HCC/PLC/liver cancer	age, alanine aminotransferase (ALT), the ratio of aspirate aminotransferase to ALT, serum HCV RNA levels and cirrhosis status and HCV genotype
	Lee-2014	V	External	High risk validation cohor	2004	2005	2008	-	HCV infected	Taiwan	5-HCC/PLC/liver cancer	age, alanine aminotransferase (ALT), the ratio of aspirate aminotransferase to ALT, serum HCV RNA levels and cirrhosis status and HCV genotype
	Lee-2014	V	External	High risk validation cohor	2004	2005	2008	-	HCV infected	Taiwan	5-HCC/PLC/liver cancer	age, alanine aminotransferase (ALT), the ratio of aspirate aminotransferase to ALT, serum HCV RNA levels and cirrhosis status and HCV genotype
10.1158/1940-6207.CAPR-15-0434	Duarte-Salles 1-2016	D	-	EPIC cohort	1991	2000	2006	4.8 years	general people	Western European	5-HCC/PLC/liver cancer	OPN, AFP
	Duarte-Salles 2-2016	D	-	EPIC cohort	1991	2000	2006	4.8 years	general people	Western European	5-HCC/PLC/liver cancer	OPN, GGT, ALP, and AST
	Duarte-Salles 3-2016	D	-	EPIC cohort	1991	2000	2006	4.8 years	general people	Western European	5-HCC/PLC/liver cancer	OPN, AFP, GGT, ALP, and AST
	Duarte-Salles 4-2016	D	-	EPIC cohort	1991	2000	2006	4.8 years	general people	Western European	5-HCC/PLC/liver cancer	GGT, ALP, and AST
	Duarte-Salles 1-2016	V	Internal	EPIC cohort	1991	2000	2006	4.8 years	general people	Western European	5-HCC/PLC/liver cancer	OPN, AFP
	Duarte-Salles 2-2016	V	Internal	EPIC cohort	1991	2000	2006	4.8 years	general people	Western European	5-HCC/PLC/liver cancer	OPN, GGT, ALP, and AST
	Duarte-Salles 3-2016	V	Internal	EPIC cohort	1991	2000	2006	4.8 years	general people	Western European	5-HCC/PLC/liver cancer	OPN, AFP, GGT, ALP, and AST
	Duarte-Salles 1-2016	D	-	EPIC cohort	1991	2000	2006	4.8 years	general people	Western European	<5-HCC/PLC/liver cancer	OPN, AFP
	Duarte-Salles 2-2016	D	-	EPIC cohort	1991	2000	2006	4.8 years	general people	Western European	<5-HCC/PLC/liver cancer	OPN, GGT, ALP, and AST
	Duarte-Salles 3-2016	D	-	EPIC cohort	1991	2000	2006	4.8 years	general people	Western European	<5-HCC/PLC/liver cancer	OPN, AFP, GGT, ALP, and AST
	Duarte-Salles 4-2016	D	-	EPIC cohort	1991	2000	2006	4.8 years	general people	Western European	<5-HCC/PLC/liver cancer	GGT, ALP, and AST
10.1002/hep.27654	FIB-4	V	External	Seoul National University Hospital	2003	2010	2010	5.4 years	HBV infected	Korea	5-HCC/PLC/liver cancer	age, AST, ALT, and PLT
	US+FIB-4	V	External	Seoul National University Hospital	2003	2011	2011	5.4 years	HBV infected	Korea	5-HCC/PLC/liver cancer	age, AST, ALT, PLT and US (ultrasound)
10.1136/gutjnl-2018-316525	AGED	D	-	a community-based prospective cohort (qidong hepatitis B infection cohort (QBC)	1996		2017		general people	China	5-HCC/PLC/liver cancer	age, gender, ALT, HBVe antigen (HBeAg) and HBV DNA
	AGED	D	-	a community-based prospective cohort (qidong hepatitis B infection cohort (QBC)	1996		2017		general people	China	10-HCC/PLC/liver cancer	age, gender, ALT, HBVe antigen (HBeAg) and HBV DNA
	AGED	D	-	a community-based prospective cohort (qidong hepatitis B infection cohort (QBC)	1996		2017		general people	China	>10-HCC/PLC/liver cancer	age, gender, ALT, HBVe antigen (HBeAg) and HBV DNA
	AGED	D	-	a community-based prospective cohort (qidong hepatitis B infection cohort (QBC)	1996		2017		general people	China	>10-HCC/PLC/liver cancer	age, gender, ALT, HBVe antigen (HBeAg) and HBV DNA
	AGED	V	External	-	2007				HBV infected	China	5-HCC/PLC/liver cancer	age, gender, ALT, HBVe antigen (HBeAg) and HBV DNA
	AGED	V	External	-	2007				HBV infected	China	10-HCC/PLC/liver cancer	age, gender, ALT, HBVe antigen (HBeAg) and HBV DNA

10.1371/journal.pone.0158066	DM-HCC risk score	D	-	Seoul National University Bundang Hospital (SNUBH), a tertiary referral center located at Seongnam, Republic of Korea	2003	-	2014	-	patients with diabetes	Korea	5-HCC/PLC/liver cancer	age, GGT, TG
	DM-HCC risk score	V	Internal	Seoul National University Bundang Hospital (SNUBH), a tertiary referral center located at Seongnam, Republic of Korea	2003	-	2014	-	patients with diabetes	Korea	5-HCC/PLC/liver cancer	age, GGT, TG
10.1093/cid/cix224	MELD	V	External	ERCHIVES	2001	-	-	-	HCV infected	U.S.	<5-SLD/advanced liver disease/cirrhosis/liver death	bilirubin, INR, creatinine
	FIB-4	V	External	ERCHIVES	2001	-	-	-	HCV infected	U.S.	<5-SLD/advanced liver disease/cirrhosis/liver death	age, AST, ALT, and PLT
	Child-Pugh;	V	External	ERCHIVES	2001	-	-	-	HCV infected	U.S.	<5-HCC/PLC/liver cancer	total bilirubin, serum albumin, international malized ratio (INR), and presence and severity of ascites and hepatic encephalopathy
	MELD	V	External	ERCHIVES	2001	-	-	-	HCV infected	U.S.	<5-HCC/PLC/liver cancer	bilirubin, INR, creatinine
	FIB-4	V	External	ERCHIVES	2001	-	-	-	HCV infected	U.S.	<5-HCC/PLC/liver cancer	age, AST, ALT, and PLT
	MELD	V	External	ERCHIVES	2001	-	-	-	HCV infected	U.S.	<5-SLD/advanced liver disease/cirrhosis/liver death	bilirubin, INR, creatinine
	FIB-4	V	External	ERCHIVES	2001	-	-	-	HCV infected	U.S.	<5-SLD/advanced liver disease/cirrhosis/liver death	age, AST, ALT, and PLT
	Child-Pugh;	V	External	ERCHIVES	2001	-	-	-	HCV infected	U.S.	<5-HCC/PLC/liver cancer	total bilirubin, serum albumin, international malized ratio (INR), and presence and severity of ascites and hepatic encephalopathy
	MELD	V	External	ERCHIVES	2001	-	-	-	HCV infected	U.S.	<5-HCC/PLC/liver cancer	bilirubin, INR, creatinine
	FIB-4	V	External	ERCHIVES	2001	-	-	-	HCV infected	U.S.	<5-HCC/PLC/liver cancer	age, AST, ALT, and PLT
	MELD	V	External	ERCHIVES	2001	-	-	-	HCV infected	U.S.	5-SLD/advanced liver disease/cirrhosis/liver death	bilirubin, INR, creatinine
	FIB-4	V	External	ERCHIVES	2001	-	-	-	HCV infected	U.S.	5-SLD/advanced liver disease/cirrhosis/liver death	age, AST, ALT, and PLT
	Child-Pugh;	V	External	ERCHIVES	2001	-	-	-	HCV infected	U.S.	5-HCC/PLC/liver cancer	total bilirubin, serum albumin, international malized ratio (INR), and presence and severity of ascites and hepatic encephalopathy
	MELD	V	External	ERCHIVES	2001	-	-	-	HCV infected	U.S.	5-HCC/PLC/liver cancer	bilirubin, INR, creatinine
	FIB-4	V	External	ERCHIVES	2001	-	-	-	HCV infected	U.S.	5-HCC/PLC/liver cancer	age, AST, ALT, and PLT
10.1002/mc.22505	Methylation Profile Score	D	-	GECC, CGMH	1988	1992	-	-	HBV infected	Taiwan	>10-HCC/PLC/liver cancer	three probes cg00300879, cg06872964, and cg07080864 related methylated sites (in the CNKSR1, IFI44L, and PENK gene)
	Methylation Profile Score	D	-	GECC, CGMH	1988	1992	-	-	HBV infected	Taiwan	>10-HCC/PLC/liver cancer	three probes cg00300879, cg06872964, and cg07080864 related methylated sites (in the CNKSR1, IFI44L, and PENK gene)
	Methylation Profile Score	D	-	multicenter study	1998	2009	-	-	HBV infected	Taiwan	>10-HCC/PLC/liver cancer	three probes cg00300879, cg06872964, and cg07080864 related methylated sites (in the CNKSR1, IFI44L, and PENK gene)
10.1371/journal.pone.0187344	Konerman-2017	V	External	UMHS cohort	1998	2014	2014	-	HCV infected	U.S.	<5-SLD/advanced liver disease/cirrhosis/liver death	APRI, Mean Baseline Platelets, Mean Platelets, Max Platelets, Mean Albumin, Max APRI, Mean Alk Phos, Mean MELD, Diff Mean Albumin, Mean INR, Diff Mean Bilirubin, Mean AFP, Diff Mean INR, Max MELD Baseline APRI Diff Mean MELD, Max AFP, Diff Mean AFP, Baseline AFP, Max Albumin
	Konerman-2017	V	External	UMHS cohort	1998	2014	2014	-	HCV infected	U.S.	<5-SLD/advanced liver disease/cirrhosis/liver death	APRI, Mean Baseline Platelets, Mean Platelets, Max Platelets, Mean Albumin, Max APRI, Mean Alk Phos, Mean MELD, Diff Mean Albumin, Mean INR, Diff Mean Bilirubin, Mean AFP, Diff Mean INR, Max MELD Baseline APRI Diff Mean MELD, Max AFP, Diff Mean AFP, Baseline AFP, Max Albumin
	Konerman-2017	V	External	UMHS cohort	1998	2014	2014	-	HCV infected	U.S.	<5-HCC/PLC/liver cancer	APRI, Mean Baseline Platelets, Mean Platelets, Max Platelets, Mean Albumin, Max APRI, Mean Alk Phos, Mean MELD, Diff Mean Albumin, Mean INR, Diff Mean Bilirubin, Mean AFP, Diff Mean INR, Max MELD Baseline APRI Diff Mean MELD, Max AFP, Diff Mean AFP, Baseline AFP, Max Albumin
	Konerman-2017	V	External	UMHS cohort	1998	2014	2014	-	HCV infected	U.S.	<5-HCC/PLC/liver cancer	APRI, Mean Baseline Platelets, Mean Platelets, Max Platelets, Mean Albumin, Max APRI, Mean Alk Phos, Mean MELD, Diff Mean Albumin, Mean INR, Diff Mean Bilirubin, Mean AFP, Diff Mean INR, Max MELD Baseline APRI Diff Mean MELD, Max AFP, Diff Mean AFP, Baseline AFP, Max Albumin
	mREACH-B	V	External	Chronic Hepatitis B patients who underwent transient elastography at tertiary centres	2006	2012	-	-	HBV infected	South Korea	<5-SLD/advanced liver disease/cirrhosis/liver death	age, gender, Alanine aminotransferase, HBeAg, Liver stiffness
	PAGE-B	V	External	Chronic Hepatitis B patients who underwent transient elastography at tertiary centres	2006	2012	-	-	HBV infected	South Korea	<5-SLD/advanced liver disease/cirrhosis/liver death	age, gender, Platelet count

	**LSM-HCC**	**V**	**External**	Chronic Hepatitis B patients who underwent transient elastography at tertiary centres	2006	2012	-	-	HBV infected	South Korea	<5-SLD/advanced liver disease/cirrhosis/liver death	LS values, age, serum albumin level, and HBV DNA level
	**mREACH-B**	**V**	**External**	Chronic Hepatitis B patients who underwent transient elastography at tertiary centres	2006	2012	-	-	HBV infected	South Korea	5-SLD/advanced liver disease/cirrhosis/liver death	age, gender, Alanine aminotransferase, HBeAg, Liver stiffness
	**PAGE-B**	**V**	**External**	Chronic Hepatitis B patients who underwent transient elastography at tertiary centres	2006	2012	-	-	HBV infected	South Korea	5-SLD/advanced liver disease/cirrhosis/liver death	age, gender, Platelet count
	**LSM-HCC**	**V**	**External**	Chronic Hepatitis B patients who underwent transient elastography at tertiary centres	2006	2012	-	-	HBV infected	South Korea	5-SLD/advanced liver disease/cirrhosis/liver death	LS values, age, serum albumin level, and HBV DNA level
	**mREACH-B**	**V**	**External**	Chronic Hepatitis B patients who underwent transient elastography at tertiary centres	2006	2012	-	-	HBV infected	South Korea	5~10-SLD/advanced liver disease/cirrhosis/liver death	age, gender, Alanine aminotransferase, HBeAg, Liver stiffness
	**PAGE-B**	**V**	**External**	Chronic Hepatitis B patients who underwent transient elastography at tertiary centres	2006	2012	-	-	HBV infected	South Korea	5~10-SLD/advanced liver disease/cirrhosis/liver death	age, gender, Platelet count
	**LSM-HCC**	**V**	**External**	Chronic Hepatitis B patients who underwent transient elastography at tertiary centres	2006	2012	-	-	HBV infected	South Korea	5~10-SLD/advanced liver disease/cirrhosis/liver death	LS values, age, serum albumin level, and HBV DNA level
**10.18632/oncotarget.22375**	**mREACH-B**	**V**	**External**	Chronic Hepatitis B patients who underwent transient elastography at tertiary centres	2006	2012	-	-	HBV infected	South Korea	<5-HCC/PLC/liver cancer	age, gender, Alanine aminotransferase, HBeAg, Liver stiffness
	**PAGE-B**	**V**	**External**	Chronic Hepatitis B patients who underwent transient elastography at tertiary centres	2006	2012	-	-	HBV infected	South Korea	<5-HCC/PLC/liver cancer	age, gender, Platelet count
	**LSM-HCC**	**V**	**External**	Chronic Hepatitis B patients who underwent transient elastography at tertiary centres	2006	2012	-	-	HBV infected	South Korea	<5-HCC/PLC/liver cancer	LS values, age, serum albumin level, and HBV DNA level
	**mREACH-B**	**V**	**External**	Chronic Hepatitis B patients who underwent transient elastography at tertiary centres	2006	2012	-	-	HBV infected	South Korea	5-HCC/PLC/liver cancer	age, gender, Alanine aminotransferase, HBeAg, Liver stiffness
	**PAGE-B**	**V**	**External**	Chronic Hepatitis B patients who underwent transient elastography at tertiary centres	2006	2012	-	-	HBV infected	South Korea	5-HCC/PLC/liver cancer	age, gender, Platelet count

	**LSM-HCC**	**V**	**External**	Chronic Hepatitis B patients who underwent transient elastography at tertiary centres	2006	2012	-	-	HBV infected	South Korea	5-HCC/PLC/liver cancer	LS values, age, serum albumin level, and HBV DNA level
	**mREACH-B**	**V**	**External**	Chronic Hepatitis B patients who underwent transient elastography at tertiary centres	2006	2012	-	-	HBV infected	South Korea	5~10-HCC/PLC/liver cancer	age, gender, Alanine aminotransferase, HBeAg, Liver stiffness
	**PAGE-B**	**V**	**External**	Chronic Hepatitis B patients who underwent transient elastography at tertiary centres	2006	2012	-	-	HBV infected	South Korea	5~10-HCC/PLC/liver cancer	age, gender, Platelet count
	**LSM-HCC**	**V**	**External**	Chronic Hepatitis B patients who underwent transient elastography at tertiary centres	2006	2012	-	-	HBV infected	South Korea	5~10-HCC/PLC/liver cancer	LS values, age, serum albumin level, and HBV DNA level
**10.1016/j.cgh.2015.10.033**	**REACH-B IIa**	**D**	-	(REVEAL)-HBV cohort	1991	1992	2008	-	HBV infected	Taiwan	<5-HCC/PLC/liver cancer	sex, age (in 5-year increments), serum ALT level, a combined variable for HBeAg serostatus, and serum HBV DNA and HBsAg levels
	**REACH-B IIb**	**D**	-	(REVEAL)-HBV cohort	1991	1992	2008	-	HBV infected	Taiwan	<5-HCC/PLC/liver cancer	sex, age, serum ALT levels, and combined variables of HBeAg and serum HBsAg levels
	**REACH-B**	**V**	**External**	(ERADICATE-B) study	1985	2000	2010	-	HBV infected	Taiwan	<5-HCC/PLC/liver cancer	Gender,age,ALT, HBeAg status, hepatitis B virus(HBV)DNA load(copies/mL)
	**REACH-B IIa**	**V**	**External**	(ERADICATE-B) study	1985	2000	2010	-	HBV infected	Taiwan	<5-HCC/PLC/liver cancer	sex, age (in 5-year increments), serum ALT level, a combined variable for HBeAg serostatus, and serum HBV DNA and HBsAg levels
	**REACH-B IIb**	**V**	**External**	(ERADICATE-B) study	1985	2000	2010	-	HBV infected	Taiwan	<5-HCC/PLC/liver cancer	sex, age, serum ALT levels, and combined variables of HBeAg and serum HBsAg levels
	**REACH-B**	**V**	**External**	(ERADICATE-B) study	1985	2000	2010	-	HBV infected	Taiwan	5-HCC/PLC/liver cancer	Gender,age,ALT, HBeAg status, hepatitis B virus(HBV)DNA load(copies/mL)
	**REACH-B IIa**	**V**	**External**	(ERADICATE-B) study	1985	2000	2010	-	HBV infected	Taiwan	5-HCC/PLC/liver cancer	sex, age (in 5-year increments), serum ALT level, a combined variable for HBeAg serostatus, and serum HBV DNA and HBsAg levels
	**REACH-B IIb**	**V**	**External**	(ERADICATE-B) study	1985	2000	2010	-	HBV infected	Taiwan	5-HCC/PLC/liver cancer	sex, age, serum ALT levels, and combined variables of HBeAg and serum HBsAg levels
	**REACH-B**	**V**	**External**	(ERADICATE-B) study	1985	2000	2010	-	HBV infected	Taiwan	10-HCC/PLC/liver cancer	Gender,age,ALT, HBeAg status, hepatitis B virus(HBV)DNA load(copies/mL)
	**REACH-B IIa**	**V**	**External**	(ERADICATE-B) study	1985	2000	2010	-	HBV infected	Taiwan	10-HCC/PLC/liver cancer	sex, age (in 5-year increments), serum ALT level, a combined variable for HBeAg serostatus, and serum HBV DNA and HBsAg levels
	**REACH-B IIb**	**V**	**External**	(ERADICATE-B) study	1985	2000	2010	-	HBV infected	Taiwan	10-HCC/PLC/liver cancer	sex, age, serum ALT levels, and combined variables of HBeAg and serum HBsAg levels
	**REACH-B**	**V**	**External**	(CUHK) study	1997	2000	2008	-	HBV infected	Hong Kang	<5-HCC/PLC/liver cancer	Gender,age,ALT, HBeAg status, hepatitis B virus(HBV)DNA load(copies/mL)
	**REACH-B IIa**	**V**	**External**	(CUHK) study	1997	2000	2008	-	HBV infected	Hong Kang	<5-HCC/PLC/liver cancer	sex, age (in 5-year increments), serum ALT level, a combined variable for HBeAg serostatus, and serum HBV DNA and HBsAg levels
	**REACH-B IIb**	**V**	**External**	(CUHK) study	1997	2000	2008	-	HBV infected	Hong Kang	<5-HCC/PLC/liver cancer	sex, age, serum ALT levels, and combined variables of HBeAg and serum HBsAg levels
	**REACH-B**	**V**	**External**	(CUHK) study	1997	2000	2008	-	HBV infected	Hong Kang	5-HCC/PLC/liver cancer	Gender,age,ALT, HBeAg status, hepatitis B virus(HBV)DNA load(copies/mL)
	**REACH-B IIa**	**V**	**External**	(CUHK) study	1997	2000	2008	-	HBV infected	Hong Kang	5-HCC/PLC/liver cancer	sex, age (in 5-year increments), serum ALT level, a combined variable for HBeAg serostatus, and serum HBV DNA and HBsAg levels
	**REACH-B IIb**	**V**	**External**	(CUHK) study	1997	2000	2008	-	HBV infected	Hong Kang	5-HCC/PLC/liver cancer	sex, age, serum ALT levels, and combined variables of HBeAg and serum HBsAg levels
	**REACH-B**	**V**	**External**	(CUHK) study	1997	2000	2008	-	HBV infected	Hong Kang	10-HCC/PLC/liver cancer	Gender,age,ALT, HBeAg status, hepatitis B virus(HBV)DNA load(copies/mL)
	**REACH-B IIa**	**V**	**External**	(CUHK) study	1997	2000	2008	-	HBV infected	Hong Kang	10-HCC/PLC/liver cancer	sex, age (in 5-year increments), serum ALT level, a combined variable for HBeAg serostatus, and serum HBV DNA and HBsAg levels
	**REACH-B IIb**	**V**	**External**	(CUHK) study	1997	2000	2008	-	HBV infected	Hong Kang	10-HCC/PLC/liver cancer	sex, age, serum ALT levels, and combined variables of HBeAg and serum HBsAg levels
	**REACH-B IIa**	**V**	**External**	(CUHK) study	1997	2000	2008	-	HBV infected	Hong Kang	<5-HCC/PLC/liver cancer	sex, age (in 5-year increments), serum ALT level, a combined variable for HBeAg serostatus, and serum HBV DNA and HBsAg levels
	**REACH-B IIa**	**V**	**External**	(CUHK) study	1997	2000	2008	-	HBV infected	Hong Kang	5-HCC/PLC/liver cancer	sex, age (in 5-year increments), serum ALT level, a combined variable for HBeAg serostatus, and serum HBV DNA and HBsAg levels
	**REACH-B IIa**	**V**	**External**	(CUHK) study	1997	2000	2008	-	HBV infected	Hong Kang	10-HCC/PLC/liver cancer	sex, age (in 5-year increments), serum ALT level, a combined variable for HBeAg serostatus, and serum HBV DNA and HBsAg levels
	**REACH-B IIb**	**V**	**External**	(CUHK) study	1997	2000	2008	-	HBV infected	Hong Kang	<5-HCC/PLC/liver cancer	sex, age, serum ALT levels, and combined variables of HBeAg and serum HBsAg levels
	**REACH-B IIb**	**V**	**External**	(CUHK) study	1997	2000	2008	-	HBV infected	Hong Kang	5-HCC/PLC/liver cancer	sex, age, serum ALT levels, and combined variables of HBeAg and serum HBsAg levels
	**REACH-B IIb**	**V**	**External**	(CUHK) study	1997	2000	2008	-	HBV infected	Hong Kang	10-HCC/PLC/liver cancer	sex, age, serum ALT levels, and combined variables of HBeAg and serum HBsAg levels
	**Li-2018**	**D**	-	Patients with type 2 diabetes	2001	2004	2011	8.33 years	patients with diabetes	Taiwan	<5-HCC/PLC/liver cancer	Age, gender, smoking, SGPT (u/l) Hba1C, comorbidity (liver cirrhosis, hepatitis B, hepatitis C), antidiabetes medication, antihyperlipidemia medication (total/high density lipoprotein cholesterol ratio)
	**Li-2018**	**D**	-	Patients with type 2 diabetes	2001	2004	2011	8.33 years	patients with diabetes	Taiwan	5-HCC/PLC/liver cancer	Age, gender, smoking, SGPT (u/l) Hba1C, ALT, comorbidity (liver cirrhosis, hepatitis B, hepatitis C), antidiabetes medication, antihyperlipidemia medication (total/high density lipoprotein cholesterol ratio)

10.1053/j.seminoncol.2018.07.006	**Li-2018**	**D**	-	Patients with type 2 diabetes	2001	2004	2011	8.33 years	patients with diabetes	Taiwan	10-HCC/PLC/liver cancer	Age, gender, smoking, SGPT (u/l) Hba1C, ALT, comorbidity (liver cirrhosis, hepatitis B, hepatitis C), antidiabetes medication, antihyperlipidemia medication (total/high density lipoprotein cholesterol ratio)
	**Li-2018**	**V**	**Internal**	Patients with type 2 diabetes	2001	2004	2011	8.33 years	patients with diabetes	Taiwan	<5-HCC/PLC/liver cancer	Age, gender, smoking, SGPT (u/l) Hba1C, ALT, comorbidity (liver cirrhosis, hepatitis B, hepatitis C), antidiabetes medication, antihyperlipidemia medication (total/high density lipoprotein cholesterol ratio)
	**Li-2018**	**V**	**Internal**	Patients with type 2 diabetes	2001	2004	2011	8.33 years	patients with diabetes	Taiwan	5-HCC/PLC/liver cancer	Age, gender, smoking, SGPT (u/l) Hba1C, ALT, comorbidity (liver cirrhosis, hepatitis B, hepatitis C), antidiabetes medication, antihyperlipidemia medication (total/high density lipoprotein cholesterol ratio)
	**Li-2018**	**V**	**Internal**	Patients with type 2 diabetes	2001	2004	2011	8.33 years	patients with diabetes	Taiwan	10-HCC/PLC/liver cancer	Age, gender, smoking, SGPT (u/l) Hba1C, ALT, comorbidity (liver cirrhosis, hepatitis B, hepatitis C), antidiabetes medication, antihyperlipidemia medication (total/high density lipoprotein cholesterol ratio)
10.3350/cmh.2020.0333	**Liang score**	**D**	-	Consesutive adult Chronic Hep B pt (CDARS data from Hospital Authority (Hong Kong))	2000	2018	-	52 months	HBV infected	Hong Kong	5-HCC/PLC/liver cancer	age, gender, HBV DNA, FIB-4
	**Liang score**	**V**	**External**	Treatment naive Korean Chronic Hep B cohort	2000	2017	-	50 months	HBV infected	South Korea	5-HCC/PLC/liver cancer	age, gender, HBV DNA, FIB-4
	**GAG-HCC**	**V**	**External**	Treatment naive Korean Chronic Hep B cohort	2000	2017	-	50 months	HBV infected	South Korea	5-HCC/PLC/liver cancer	Age, gender, core promoter mutaiton, level of HBV DNA, cirrhosis
	**REACH-B**	**V**	**External**	Treatment naive Korean Chronic Hep B cohort	2000	2017	-	50 months	HBV infected	South Korea	5-HCC/PLC/liver cancer	Age, gender, ALT, HBeAg, HBV DNA
	**CU-HCC**	**V**	**External**	Treatment naive Korean Chronic Hep B cohort	2000	2017	-	50 months	HBV infected	South Korea	5-HCC/PLC/liver cancer	-
10.1093/ije/dyaa089	**Sinn-2020**	**D**	-	National Health Insurance Service (NHIS)-National Sample Cohort (NSC) (representative sample of 2.2% of Korean citizens enroled in NHIS) - all men and women 20 years of age participating in the NHIS-NSC cohort with at least one health screening between 1 January 2003 and 31 December 2013	2003	2013	-	-	general people	South Korea	10-HCC/PLC/liver cancer	Age, gender, smoking, diabetes, ALT, total cholesterol
	**Sinn-2020**	**V**	**External**	Samsung Medical Center Health Promotion Center Cohort (SMC-HPCC) - men and women 20 years of age who underwent comprehensive health check-up examinations between January 2003 and December 2013, and additional follow-up between January 2003 and November 2017 at Samsung Medical Center,	2003	2013	2017	-	general people	South Korea	10-HCC/PLC/liver cancer	Age, gender, smoking, diabetes, ALT, total cholesterol

10.1111/jvh.13185	Sinn-2019	D	-	Across four academic institutions in South Korea: patients who met all of the following inclusion criteria: (a) adults aged 18 years or older, (b) patients with chronic hepatitis B, defined by presence of hepatitis B surface antigen (HBsAg) for more than 6 months or compatible history, (c) HBV treatment-naïve, (d) no co-infection with hepatitis C virus or human immunodeficiency virus and (e) no history of malignancy including HCC at baseline	2006	2011	-	-	HBV infected	South Korea	5-HCC/PLC/liver cancer	Age, gender, HBeAg, ALT level, platelet count, cirrhosis
10.1002/hep4.1700	dAAR score	D	-	FINRISK	2002	2012	-	-	general people	Finland	5~10-SLD/advanced liver disease/cirrhosis/liver death	age, AAR(aspartate-to-alanine aminotransferase ratio), and ALT
	dAAR score	V	External	Swedish Apolipoprotein Mortality Risk [AMORIS] subcohort	1985	1996	-	-	general people	Sweden	5-SLD/advanced liver disease/cirrhosis/liver death	age, AAR, and ALT
	dAAR score	V	External	Swedish Apolipoprotein Mortality Risk [AMORIS] subcohort	1985	1996	-	-	general people	Sweden	10-SLD/advanced liver disease/cirrhosis/liver death	age, AAR, and ALT
	dAAR score	V	External	Swedish Apolipoprotein Mortality Risk [AMORIS] subcohort	1985	1996	-	-	general people	Sweden	>10-SLD/advanced liver disease/cirrhosis/liver death	age, AAR, and ALT
	FIB-4	V	External	Swedish Apolipoprotein Mortality Risk [AMORIS] subcohort	1985	1996	-	-	general people	Sweden	10-SLD/advanced liver disease/cirrhosis/liver death	age, AST, ALT, and PLT
	APRI	V	External	Swedish Apolipoprotein Mortality Risk [AMORIS] subcohort	1985	1996	-	-	general people	Sweden	10-SLD/advanced liver disease/cirrhosis/liver death	AST, platelet count
	NAFLD Fibrosis Score	V	External	Swedish Apolipoprotein Mortality Risk [AMORIS] subcohort	1985	1996	-	-	general people	Sweden	10-SLD/advanced liver disease/cirrhosis/liver death	age (years) ; BMI (kg/m2) ; diabetes ; AST/ALT ; platelet count ;albumin (g/dL)
	dAAR score	V	External	Swedish Apolipoprotein Mortality Risk [AMORIS] subcohort	1985	2009	-	-	patients with NAFLD	Sweden	5-SLD/advanced liver disease/cirrhosis/liver death	age, AAR, and ALT
	dAAR score	V	External	Swedish Apolipoprotein Mortality Risk [AMORIS] subcohort	1985	2009	-	-	patients with NAFLD	Sweden	10-SLD/advanced liver disease/cirrhosis/liver death	age, AAR, and ALT
	dAAR score	V	External	Swedish Apolipoprotein Mortality Risk [AMORIS] subcohort	1985	2009	-	-	patients with NAFLD	Sweden	>10-SLD/advanced liver disease/cirrhosis/liver death	age, AAR, and ALT
	FIB-4	V	External	Swedish Apolipoprotein Mortality Risk [AMORIS] subcohort	1985	2009	-	-	patients with NAFLD	Sweden	10-SLD/advanced liver disease/cirrhosis/liver death	age, AST, ALT, and PLT
	APRI	V	External	Swedish Apolipoprotein Mortality Risk [AMORIS] subcohort	1985	2009	-	-	patients with NAFLD	Sweden	10-SLD/advanced liver disease/cirrhosis/liver death	AST, platelet count
	NAFLD Fibrosis Score	V	External	Swedish Apolipoprotein Mortality Risk [AMORIS] subcohort	1985	2009	-	-	patients with NAFLD	Sweden	10-SLD/advanced liver disease/cirrhosis/liver death	age (years) ; BMI (kg/m2) ; diabetes ; AST/ALT ; platelet count ;albumin (g/dL)

	**dAAR score**	**V**	**External**	patients with biopsy-proven NAFLD at Karolinska University Hospital, Huddinge, and Linköping University Hospital	1985	2009	-	-	patients with NAFLD	Sweden	>10-SLD/advanced liver disease/cirrhosis/liver death	age, AAR, and ALT
**10.21147/J.ISSN.1000-9604.2021.03.07**	**MALE-ABCD**	**D**	**-**	CCOP-LC Cohort	2017	-	2019	2 years	HBV infected	China	<5-HCC/PLC/liver cancer	Age, AFP, DCP, GGT, PLT and WBC
	**MALE-ABCD**	**V**	**Internal**	CCOP-LC Cohort	2017	-	2019	2 years	HBV infected	China	<5-HCC/PLC/liver cancer	Age, AFP, DCP, GGT, PLT and WBC
	**AGED**	**V**	**External**	CCOP-LC Cohort	2017	-	2019	2 years	HBV infected	China	<5-HCC/PLC/liver cancer	sex, age, HbeAg, HBV DNA(log copies/mL)
	**REACH-B**	**V**	**External**	CCOP-LC Cohort	2017	-	2019	2 years	HBV infected	China	<5-HCC/PLC/liver cancer	sex, age, ALT, HBeAg status and HBV DNA
	**PAGE-B**	**V**	**External**	CCOP-LC Cohort	2017	-	2019	2 years	HBV infected	China	<5-HCC/PLC/liver cancer	age, sex and platelets
	**mPAGE-B**	**V**	**External**	CCOP-LC Cohort	2017	-	2019	2 years	HBV infected	China	<5-HCC/PLC/liver cancer	platelet, age, sex, albumin
	**THRI**	**V**	**External**	CCOP-LC Cohort	2017	-	2019	2 years	HBV infected	China	<5-HCC/PLC/liver cancer	Age, Etiology, Gender, Platelets
	**GALAD**	**V**	**External**	CCOP-LC Cohort	2017	-	2019	2 years	HBV infected	China	<5-HCC/PLC/liver cancer	age, sex, AFP, AFP-L3 (an isoform of AFP characterized by the presence of an a 1-6–linked residue on the AFP carbohydrate side chain), DCP
**10.1136/gutjnl-2016-312993**	**RWS-HCC**	**V**	**External**	non-Asian：Gastroenterology and Hepatology, University Hospital of Cologne, Germany	1994	2015	-	-	HBV infected	Germany	10-HCC/PLC/liver cancer	age, gender, cirrhosis,serum AFP
**10.1111/jvh.13631**	**CAP-B Score**	**D**	**-**	Adult patients with CHB diagnosis from the NHIS database	2005	2015	2017	-	HBV infected	South Korea	<5-SLD/advanced liver disease/cirrhosis/liver death	Sex, age, medical aid, income, chronic hepatitis C, diabetes mellitus, statin exposure, anitplatelet exposure, smoking, ALT, GGT*alcohol consumption
	**CAP-B Score**	**D**	**-**	Adult patients with CHB diagnosis from the NHIS database	2005	2015	2017	-	HBV infected	South Korea	5-SLD/advanced liver disease/cirrhosis/liver death	Sex, age, medical aid, income, chronic hepatitis C, diabetes mellitus, statin exposure, anitplatelet exposure, smoking, ALT, GGT*alcohol consumption
	**CAP-B Score**	**D**	**-**	Adult patients with CHB diagnosis from the NHIS database	2005	2015	2017	-	HBV infected	South Korea	10-SLD/advanced liver disease/cirrhosis/liver death	Sex, age, medical aid, income, chronic hepatitis C, diabetes mellitus, statin exposure, anitplatelet exposure, smoking, ALT, GGT*alcohol consumption
	**CAP-B Score**	**V**	**Internal**	Adult patients with CHB diagnosis from the NHIS database	2005	2015	2017	-	HBV infected	South Korea	<5-SLD/advanced liver disease/cirrhosis/liver death	Sex, age, medical aid, low income, intermediate income, chronic hepatitis C, diabetes mellitus, statin exposure, anitplatelet exposure, smoking, ALT, GGT*alcohol consumption
	**CAP-B Score**	**V**	**Internal**	Adult patients with CHB diagnosis from the NHIS database	2005	2015	2017	-	HBV infected	South Korea	5-SLD/advanced liver disease/cirrhosis/liver death	Sex, age, medical aid, low income, intermediate income, chronic hepatitis C, diabetes mellitus, statin exposure, anitplatelet exposure, smoking, ALT, GGT*alcohol consumption
	**CAP-B Score**	**V**	**Internal**	Adult patients with CHB diagnosis from the NHIS database	2005	2015	2017	-	HBV infected	South Korea	10-SLD/advanced liver disease/cirrhosis/liver death	Sex, age, medical aid, low income, intermediate income, chronic hepatitis C, diabetes mellitus, statin exposure, anitplatelet exposure, smoking, ALT, GGT*alcohol consumption
**10.1053/j.gastro.2019.09.008**	**APRI**	**V**	**External**	AMORIS	1985	1996	-	-	general people	Sweden	5-SLD/advanced liver disease/cirrhosis/liver death	AST, platelet count
	**APRI**	**V**	**External**	AMORIS	1985	1996	-	-	general people	Sweden	10-SLD/advanced liver disease/cirrhosis/liver death	AST, platelet count
	**APRI**	**V**	**External**	AMORIS	1985	1996	-	-	general people	Sweden	>10-SLD/advanced liver disease/cirrhosis/liver death	AST, platelet count
	**BARD**	**V**	**External**	AMORIS	1985	1996	-	-	general people	Sweden	5-SLD/advanced liver disease/cirrhosis/liver death	BMI, AST, ALT, diabetes
	**BARD**	**V**	**External**	AMORIS	1985	1996	-	-	general people	Sweden	10-SLD/advanced liver disease/cirrhosis/liver death	BMI, AST, ALT, diabetes
	**BARD**	**V**	**External**	AMORIS	1985	1996	-	-	general people	Sweden	>10-SLD/advanced liver disease/cirrhosis/liver death	BMI, AST, ALT, diabetes
	**FIB-4**	**V**	**External**	AMORIS	1985	1996	-	-	general people	Sweden	5-SLD/advanced liver disease/cirrhosis/liver death	Age, AST, platelets, ALT
	**FIB-4**	**V**	**External**	AMORIS	1985	1996	-	-	general people	Sweden	10-SLD/advanced liver disease/cirrhosis/liver death	Age, AST, platelets, ALT
	**FIB-4**	**V**	**External**	AMORIS	1985	1996	-	-	general people	Sweden	>10-SLD/advanced liver disease/cirrhosis/liver death	Age, AST, platelets, ALT
	**Forns Score**	**V**	**External**	AMORIS	1985	1996	-	-	general people	Sweden	5-SLD/advanced liver disease/cirrhosis/liver death	Platelet count, GT, age, cholesterol
	**Forns Score**	**V**	**External**	AMORIS	1985	1996	-	-	general people	Sweden	10-SLD/advanced liver disease/cirrhosis/liver death	Platelet count, GT, age, cholesterol
	**Forns Score**	**V**	**External**	AMORIS	1985	1996	-	-	general people	Sweden	>10-SLD/advanced liver disease/cirrhosis/liver death	Platelet count, GT, age, cholesterol
	**NAFLD Fibrosis Score**	**V**	**External**	AMORIS	1985	1996	-	-	general people	Sweden	5-SLD/advanced liver disease/cirrhosis/liver death	age, BMI, diabetes, AST, ALT, platelet count, albumin
	**NAFLD Fibrosis Score**	**V**	**External**	AMORIS	1985	1996	-	-	general people	Sweden	10-SLD/advanced liver disease/cirrhosis/liver death	age, BMI, diabetes, AST, ALT, platelet count, albumin
	**NAFLD Fibrosis Score**	**V**	**External**	AMORIS	1985	1996	-	-	general people	Sweden	>10-SLD/advanced liver disease/cirrhosis/liver death	age, BMI, diabetes, AST, ALT, platelet count, albumin
	**APRI**	**V**	**External**	AMORIS	1985	1996	-	-	general people	Sweden	10-SLD/advanced liver disease/cirrhosis/liver death	AST, platelet count
	**BARD**	**V**	**External**	AMORIS	1985	1996	-	-	general people	Sweden	10-SLD/advanced liver disease/cirrhosis/liver death	BMI, AST, ALT, diabetes
	**FIB-4**	**V**	**External**	AMORIS	1985	1996	-	-	general people	Sweden	10-SLD/advanced liver disease/cirrhosis/liver death	Age, AST, platelets, ALT
	**Forns Score**	**V**	**External**	AMORIS	1985	1996	-	-	general people	Sweden	10-SLD/advanced liver disease/cirrhosis/liver death	Platelet count, GT, age, cholesterol
	**NAFLD Fibrosis Score**	**V**	**External**	AMORIS	1985	1996	-	-	general people	Sweden	10-SLD/advanced liver disease/cirrhosis/liver death	age, BMI, diabetes, AST, ALT, platelet count, albumin
	**D^2AS**	**D**	**-**	patients who received care at Samsung Medical Center, Seoul, Korea	2006	2011	2014	-	HBV infected	South Korea	<5-HCC/PLC/liver cancer	Sex, age, ALT, HBeAg and HBV DNA levels
	**D^2AS**	**D**	**-**	patients who received care at Samsung Medical Center, Seoul, Korea	2006	2011	2014	-	HBV infected	South Korea	<5-HCC/PLC/liver cancer	Sex, age, ALT, HBeAg and HBV DNA levels
	**D^2AS**	**D**	**-**	patients who received care at Samsung Medical Center, Seoul, Korea	2006	2011	2014	-	HBV infected	South Korea	<5-HCC/PLC/liver cancer	Sex, age, ALT, HBeAg and HBV DNA levels

10.5009/gnl16403	D^2AS	D	-	patients who received care at Samsung Medical Center, Seoul, Korea	2006	2011	2014	-	HBV infected	South Korea	5-HCC/PLC/liver cancer	Sex, age, ALT, HBeAg and HBV DNA levels
	D^2AS	D	-	patients who received care at Samsung Medical Center, Seoul, Korea	2006	2011	2014	-	HBV infected	South Korea	5~10-HCC/PLC/liver cancer	Sex, age, ALT, HBeAg and HBV DNA levels
	D^2AS	V	External	f patients who received care at Seoul National University Hospital	2006	2011	2014	-	HBV infected	South Korea	<5-HCC/PLC/liver cancer	Sex, age, ALT, HBeAg and HBV DNA levels
	D^2AS	V	External	f patients who received care at Seoul National University Hospital	2006	2011	2014	-	HBV infected	South Korea	<5-HCC/PLC/liver cancer	Sex, age, ALT, HBeAg and HBV DNA levels
	D^2AS	V	External	f patients who received care at Seoul National University Hospital	2006	2011	2014	-	HBV infected	South Korea	<5-HCC/PLC/liver cancer	Sex, age, ALT, HBeAg and HBV DNA levels
	D^2AS	V	External	f patients who received care at Seoul National University Hospital	2006	2011	2014	-	HBV infected	South Korea	5-HCC/PLC/liver cancer	Sex, age, ALT, HBeAg and HBV DNA levels
	D^2AS	V	External	f patients who received care at Seoul National University Hospital	2006	2011	2014	-	HBV infected	South Korea	5~10-HCC/PLC/liver cancer	Sex, age, ALT, HBeAg and HBV DNA levels
10.1186/s12885-021-08498-w	An-2021	D	-	NHIS-2020-2-146	2004	2007	2015	11.1 years	general people	South Korea	5~10-HCC/PLC/liver cancer	age, sex, obesity, income level, the family history of chronic liver disease, ALT, GGT, total blood cholesterol level, and preexisting chronic liver disease, chronic hepatitis virus infection, HIV infection, DM, dyslipidemia, or schizophrenic/delusional disorders or mental disorders due to psychoactive substance use
	An-2021	V	Internal	NHIS-2020-2-146	2004	2007	2015	9.1 years	general people	South Korea	5~10-HCC/PLC/liver cancer	age, sex, obesity, liver function tests, the family history of chronic liver disease, underlying chronic liver disease, chronic hepatitis virus or human immunodeficiency virus infection, and diabetes mellitus income, total cholesterol, and underlying dyslipidemia or schizophrenic/delusional disorders
10.1002/ijc.33487	CKB-PLR	D	-	China Kadoorie Biobank	2004	2008	2017	-	general people	China	10-HCC/PLC/liver cancer	Age, gender, residential area, level of education, previous cancer diagnosis,family histroy of cancer, history of cirrhosis or chronic hepatitis, history of gall stone or gall bladder disease, BMI, physical activity, random glucose, alcohol, smoking
	CKB-PLR	V	Internal	China Kadoorie Biobank	2004	2008	2017	-	general people	China	10-HCC/PLC/liver cancer	Age, gender, residential area, level of education, previous cancer diagnosis,family histroy of cancer, history of cirrhosis or chronic hepatitis, history of gall stone or gall bladder disease, BMI, physical activity, random glucose, alcohol, smoking
	GAG-HCC	V	External	Screening and Surveillance of Hepatocellular Carcinoma Project at Chulabhorn Hospital, Bangkok, Thailand, during July 2010	2010	2017	-	-	HBV infected	Thailand	5-HCC/PLC/liver cancer	liver cirrhosis, age, sex, hepatitis B virus DNA load
	CU-HCC	V	External	Screening and Surveillance of Hepatocellular Carcinoma Project at Chulabhorn Hospital, Bangkok, Thailand, during July 2010	2010	2017	-	-	HBV infected	Thailand	5-HCC/PLC/liver cancer	serum albumin, bilirubin levels, liver stiffness
	REACH-B	V	External	Screening and Surveillance of Hepatocellular Carcinoma Project at Chulabhorn Hospital, Bangkok, Thailand, during July 2010	2010	2017	-	-	HBV infected	Thailand	5-HCC/PLC/liver cancer	sex, age, ALT, HBeAg status and HBVDNA
	PAGE-B	V	External	Screening and Surveillance of Hepatocellular Carcinoma Project at Chulabhorn Hospital, Bangkok, Thailand, during July 2010	2010	2017	-	-	HBV infected	Thailand	5-HCC/PLC/liver cancer	platelet, age, sex

10.1111/jvh.13517	**mPAGE-B**	**V**	**External**	Screening and Surveillance of Hepatocellular Carcinoma Project at Chulabhorn Hospital, Bangkok, Thailand, during July 2010	2010	2017	-	-	HBV infected	Thailand	5-HCC/PLC/liver cancer	platelet, age, sex, albumin
	**CAMD**	**V**	**External**	Screening and Surveillance of Hepatocellular Carcinoma Project at Chulabhorn Hospital, Bangkok, Thailand, during July 2010	2010	2017	-	-	HBV infected	Thailand	5-HCC/PLC/liver cancer	cirrhosis, age, sex, diabetes
	**AASL**	**V**	**External**	Screening and Surveillance of Hepatocellular Carcinoma Project at Chulabhorn Hospital, Bangkok, Thailand, during July 2010	2010	2017	-	-	HBV infected	Thailand	5-HCC/PLC/liver cancer	age, albumin, sex, cirrhosis
	**LSM-HCC**	**V**	**External**	Screening and Surveillance of Hepatocellular Carcinoma Project at Chulabhorn Hospital, Bangkok, Thailand, during July 2010	2010	2017	-	-	HBV infected	Thailand	5-HCC/PLC/liver cancer	liver stiffness, age, albumin, HBV DNA
	**mREACH-B**	**V**	**External**	Screening and Surveillance of Hepatocellular Carcinoma Project at Chulabhorn Hospital, Bangkok, Thailand, during July 2010	2010	2017	-	-	HBV infected	Thailand	5-HCC/PLC/liver cancer	sex, age, ALT, HBeAg, liver stiffness
10.3390/v14040732	**PAGE-B**	**V**	**External**	Liver Studies Center of the University Hospital of the Federal University of Maranhão	-	-	-	-	HBV infected	Brazil	<5-HCC/PLC/liver cancer	age, sex and platelets
	**PAGE-B**	**V**	**External**	Liver Studies Center of the University Hospital of the Federal University of Maranhão	-	-	-	-	HBV infected	Brazil	<5-HCC/PLC/liver cancer	age, sex and platelets
	**PAGE-B**	**V**	**External**	Liver Studies Center of the University Hospital of the Federal University of Maranhão	-	-	-	-	HBV infected	Brazil	<5-HCC/PLC/liver cancer	age, sex and platelets
	**REACH-B**	**V**	**External**	Liver Studies Center of the University Hospital of the Federal University of Maranhão	-	-	-	-	HBV infected	Brazil	<5-HCC/PLC/liver cancer	sex, age, ALT, HBeAg status and HBVDNA
	**REACH-B**	**V**	**External**	Liver Studies Center of the University Hospital of the Federal University of Maranhão	-	-	-	-	HBV infected	Brazil	<5-HCC/PLC/liver cancer	sex, age, ALT, HBeAg status and HBVDNA
	**REACH-B**	**V**	**External**	Liver Studies Center of the University Hospital of the Federal University of Maranhão	-	-	-	-	HBV infected	Brazil	<5-HCC/PLC/liver cancer	sex, age, ALT, HBeAg status and HBVDNA
	**Modified HLI**	**V**	**Internal**	(EPIC) cohort	1992	2000	2010	-	general people	Denmark, France, Ger many, Greece, Italy, Netherlands, Norway, Spain, Sweden, and the United Kingdom	10-HCC/PLC/liver cancer	diet, BMI, physical activity, lifetime alcohol, smoking, diabetes, and hepatitis
	**Lifestyle signature**	**D**	-	(EPIC) cohort	1992	2000	2010	-	general people	Denmark, France, Ger many, Greece, Italy, Netherlands, Norway, Spain, Sweden, and the United Kingdom	10-HCC/PLC/liver cancer	diet, BMI, physical activity, lifetime alcohol, smoking, diabetes, and hepatitis

10.1093/ajcn/nqy074	**Metabolic signature**	**D**	-	(EPIC) cohort	1992	2000	2010	-	general people	Denmark, France, Germany, Greece, Italy, Netherlands, Norway, Spain, Sweden, and the United Kingdom	10-HCC/PLC/liver cancer	Glutamic acid, Hexoses, SM(OH)C14:1, SM(OH)C16:1, SM(OH)C22:2, PCaaC32:1, Liver function score
	**LFS**	**D**	-	(EPIC) cohort	1992	2000	2010	-	general people	Denmark, France, Germany, Greece, Italy, Netherlands, Norway, Spain, Sweden, and the United Kingdom	10-HCC/PLC/liver cancer	ALT,AST, GGT, alkaline phosphatase, albumin ,total bilirubin
	**LFS + lifestyle signature**	**D**	-	(EPIC) cohort	1992	2000	2010	-	general people	Denmark, France, Germany, Greece, Italy, Netherlands, Norway, Spain, Sweden, and the United Kingdom	10-HCC/PLC/liver cancer	diet, BMI, physical activity, lifetime alcohol, smoking, diabetes, and hepatitis
	**LFS + metabolic signature**	**D**	-	(EPIC) cohort	1992	2000	2010	-	general people	Denmark, France, Germany, Greece, Italy, Netherlands, Norway, Spain, Sweden, and the United Kingdom	10-HCC/PLC/liver cancer	Glutamic acid, Hexoses, SMC16:1, SM(OH)C14:1, SM(OH)C22:2, LysoPC aC28:1, PC aeC30:2
10.1038/ajg.2017.254	**FIB-4**	**V**	**External**	ERADICATE-B	1985	2000	-	-	HBV infected	Taiwan	>10-SLD/advanced liver disease/cirrhosis/liver death	age, AST, platelet counts, ALT
10.1038/s41416-022-01851-1	**aMAP score**	**V**	**External**	HCC surveillance programme at the Ogaki Municipal Hospital	1998	2014	2021	11.8 years	general people	Japan	>10-HCC/PLC/liver cancer	Age, sex , bilirubin ,albumin, platelets
10.2147/ott.S51986	**Transient elastography-based risk estimation**	**D**	-	-	2005	2007	-	-	HBV infected	Korea	<5-HCC/PLC/liver cancer	age, male gender, and liver stiffness values,hepatitis B virus DNA
	**Transient elastography-based risk estimation**	**V**	**Internal**	-	2005	2007	-	-	HBV infected	Korea	<5-HCC/PLC/liver cancer	age, male gender, and liver stiffness values,hepatitis B virus DNA
10.1371/journal.pone.0208141	**CS Cox model (average)**	**D**	-	VHA data	2000	2016	-	7 years	HCV infected	US	<5-SLD/advanced liver disease/cirrhosis/liver death	Variable selection frequency for CS Cox model：see supplementary
	**CS Cox model (average)**	**D**	-	VHA data	2000	2016	-	7 years	HCV infected	US	<5-SLD/advanced liver disease/cirrhosis/liver death	Variable selection frequency for CS Cox model：see supplementary
	**CS Cox model (average)**	**D**	-	VHA data	2000	2016	-	7 years	HCV infected	US	5-SLD/advanced liver disease/cirrhosis/liver death	Variable selection frequency for CS Cox model：see supplementary
	**longitudinal Cox model (average)**	**D**	-	VHA data	2000	2016	-	7 years	HCV infected	US	<5-SLD/advanced liver disease/cirrhosis/liver death	Variable selection frequency for CS Cox model：see supplementary
	**longitudinal Cox model (average)**	**D**	-	VHA data	2000	2016	-	7 years	HCV infected	US	<5-SLD/advanced liver disease/cirrhosis/liver death	Variable selection frequency for CS Cox model：see supplementary
	**longitudinal Cox model (average)**	**D**	-	VHA data	2000	2016	-	7 years	HCV infected	US	5-SLD/advanced liver disease/cirrhosis/liver death	Variable selection frequency for CS Cox model：see supplementary
	**CS boosting model (average)**	**D**	-	VHA data	2000	2016	-	7 years	HCV infected	US	<5-SLD/advanced liver disease/cirrhosis/liver death	Variable selection frequency for CS Cox model：see supplementary
	**CS boosting model (average)**	**D**	-	VHA data	2000	2016	-	7 years	HCV infected	US	<5-SLD/advanced liver disease/cirrhosis/liver death	Variable selection frequency for CS Cox model：see supplementary
	**CS boosting model (average)**	**D**	-	VHA data	2000	2016	-	7 years	HCV infected	US	5-SLD/advanced liver disease/cirrhosis/liver death	Variable selection frequency for CS Cox model：see supplementary
	**longitudinal boosting model (average)**	**D**	-	VHA data	2000	2016	-	7 years	HCV infected	US	<5-SLD/advanced liver disease/cirrhosis/liver death	Variable selection frequency for CS Cox model：see supplementary
	**longitudinal boosting model (average)**	**D**	-	VHA data	2000	2016	-	7 years	HCV infected	US	<5-SLD/advanced liver disease/cirrhosis/liver death	Variable selection frequency for CS Cox model：see supplementary
	**longitudinal boosting model (average)**	**D**	-	VHA data	2000	2016	-	7 years	HCV infected	US	5-SLD/advanced liver disease/cirrhosis/liver death	Variable selection frequency for CS Cox model：see supplementary
	**CS Cox model (closest)**	**D**	-	VHA data	2000	2016	-	7 years	HCV infected	US	<5-SLD/advanced liver disease/cirrhosis/liver death	Variable selection frequency for CS Cox model：see supplementary
	**CS boosting model (closest)**	**D**	-	VHA data	2000	2016	-	7 years	HCV infected	US	<5-SLD/advanced liver disease/cirrhosis/liver death	Variable selection frequency for CS Cox model：see supplementary
	**longitudinal Cox model (closest)**	**D**	-	VHA data	2000	2016	-	7 years	HCV infected	US	<5-SLD/advanced liver disease/cirrhosis/liver death	Variable selection frequency for CS Cox model：see supplementary
	**longitudinal boosting model (closest)**	**D**	-	VHA data	2000	2016	-	7 years	HCV infected	US	<5-SLD/advanced liver disease/cirrhosis/liver death	Variable selection frequency for CS Cox model：see supplementary
	**CS Cox model (closest)**	**D**	-	VHA data	2000	2016	-	7 years	HCV infected	US	<5-SLD/advanced liver disease/cirrhosis/liver death	Variable selection frequency for CS Cox model：see supplementary
	**CS boosting model (closest)**	**D**	-	VHA data	2000	2016	-	7 years	HCV infected	US	<5-SLD/advanced liver disease/cirrhosis/liver death	Variable selection frequency for CS Cox model：see supplementary
	**longitudinal Cox model (closest)**	**D**	-	VHA data	2000	2016	-	7 years	HCV infected	US	<5-SLD/advanced liver disease/cirrhosis/liver death	Variable selection frequency for CS Cox model：see supplementary
	**longitudinal boosting model (closest)**	**D**	-	VHA data	2000	2016	-	7 years	HCV infected	US	<5-SLD/advanced liver disease/cirrhosis/liver death	Variable selection frequency for CS Cox model：see supplementary
	**CS Cox model (closest)**	**D**	-	VHA data	2000	2016	-	7 years	HCV infected	US	5-SLD/advanced liver disease/cirrhosis/liver death	Variable selection frequency for CS Cox model：see supplementary
	**CS boosting model (closest)**	**D**	-	VHA data	2000	2016	-	7 years	HCV infected	US	5-SLD/advanced liver disease/cirrhosis/liver death	Variable selection frequency for CS Cox model：see supplementary
	**longitudinal Cox model (closest)**	**D**	-	VHA data	2000	2016	-	7 years	HCV infected	US	5-SLD/advanced liver disease/cirrhosis/liver death	Variable selection frequency for CS Cox model：see supplementary
	**longitudinal boosting model (closest)**	**D**	-	VHA data	2000	2016	-	7 years	HCV infected	US	5-SLD/advanced liver disease/cirrhosis/liver death	Variable selection frequency for CS Cox model：see supplementary
	**Le-2021**	**D**	-	San Francisco Bay Area Cohort	2000	2016	-	-	HBV infected	US	<5-SLD/advanced liver disease/cirrhosis/liver death	Sex, age, diabetes, antiviral treatment status/duration, hepatitis B e-antigen, ALT, AST
	**Le-2021**	**D**	-	San Francisco Bay Area Cohort	2000	2016	-	-	HBV infected	US	5-SLD/advanced liver disease/cirrhosis/liver death	Sex, age, diabetes, antiviral treatment status/duration, hepatitis B e-antigen, ALT, AST
	**Le-2021**	**D**	-	San Francisco Bay Area Cohort	2000	2016	-	-	HBV infected	US	10-SLD/advanced liver disease/cirrhosis/liver death	Sex, age, diabetes, antiviral treatment status/duration, hepatitis B e-antigen, ALT, AST
10.1093/infdis/jiaa330	**Le-2021**	**V**	**External**	Kaohsiung Medical University Hospital in Kaohsiung, Taiwan	-	-	-	-	HBV infected	Taiwan	<5-SLD/advanced liver disease/cirrhosis/liver death	Sex, age, diabetes, antiviral treatment status/duration, hepatitis B e-antigen, ALT, AST

	**Le-2021**	**V**	**External**	Kaohsiung Medical University Hospital in Kaohsiung, Taiwan	-	-	-	-	HBV infected	Taiwan	5-SLD/advanced liver disease/cirrhosis/liver death	Sex, age, diabetes, antiviral treatment status/duration, hepatitis B e-antigen, ALT, AST
	**Le-2021**	**V**	**External**	Kaohsiung Medical University Hospital in Kaohsiung, Taiwan	-	-	-	-	HBV infected	Taiwan	10-SLD/advanced liver disease/cirrhosis/liver death	Sex, age, diabetes, antiviral treatment status/duration, hepatitis B e-antigen, ALT, AST
**10.3389/fonc.2021.762662**	**Cao-2021**	**D**	-	CCOP-LC cohort	2017	-	2020	-	HBV infected	Binhai county,Lingbi county, Mengcheng county, Sheyang county, Shenqiu county, Dancheng county, and Yingdong county	<5-HCC/PLC/liver cancer	Sex, Age ,BMI ,Alcohol consumption (g/week ethanol), Liver diseases in mothers, Psychological trauma
	**Cao-2021**	**D**	-	CCOP-LC cohort	2017	-	2020	-	HBV infected	Binhai county,Lingbi county, Mengcheng county, Sheyang county, Shenqiu county, Dancheng county, and Yingdong county	<5-HCC/PLC/liver cancer	Sex, Age ,BMI ,Alcohol consumption (g/week ethanol), Liver diseases in mothers, Psychological trauma
	**Cao-2021**	**D**	-	CCOP-LC cohort	2017	-	2020	-	HBV infected	Binhai county,Lingbi county, Mengcheng county, Sheyang county, Shenqiu county, Dancheng county, and Yingdong county	<5-HCC/PLC/liver cancer	Sex, Age ,BMI ,Alcohol consumption (g/week ethanol), Liver diseases in mothers, Psychological trauma
	**Cao-2021**	**V**	**Internal**	CCOP-LC cohort	2017	-	2020	-	HBV infected	Binhai county,Lingbi county, Mengcheng county, Sheyang county, Shenqiu county, Dancheng county, and Yingdong county	<5-HCC/PLC/liver cancer	Sex, Age ,BMI ,Alcohol consumption (g/week ethanol), Liver diseases in mothers, Psychological trauma
**10.1016/j.cgh.2018.11.030**	**NAFLD Fibrosis Score**	**V**	**External**	Karolinska University Hospital (Huddinge) and Link-öping University Hospital	1971	2009	2014	19.9 years	patients with NAFLD	Sweden	>10-SLD/advanced liver disease/cirrhosis/liver death	age, BMI, hyperglycemia or diabetes, AST/ALT ratio, platelet, albumin
	**FIB-4**	**V**	**External**	Karolinska University Hospital (Huddinge) and Link-öping University Hospital	1971	2009	2014	19.9 years	patients with NAFLD	Sweden	>10-SLD/advanced liver disease/cirrhosis/liver death	Platelets, age, aspartate aminotransferase (AST), ALT
	**APRI**	**V**	**External**	Karolinska University Hospital (Huddinge) and Link-öping University Hospital	1971	2009	2014	19.9 years	patients with NAFLD	Sweden	>10-SLD/advanced liver disease/cirrhosis/liver death	AST, platelet count
	**BARD**	**V**	**External**	Karolinska University Hospital (Huddinge) and Link-öping University Hospital	1971	2009	2014	19.9 years	patients with NAFLD	Sweden	>10-SLD/advanced liver disease/cirrhosis/liver death	BMI, AST, ALT, diabetes
	**LS-Based Model 1**	**D**	-	Severance Hospital, Samsung Medical Center, and Gangnam Severance Hospital	2012	2020	-	60.7 months	patients with NAFLD	Korea	5-HCC/PLC/liver cancer	age, platelet count, and categorized LS,together with AST level (>34 IU/mL)
	**LS-Based Model 1**	**D**	-	Severance Hospital, Samsung Medical Center, and Gangnam Severance Hospital	2012	2020	-	60.7 months	patients with NAFLD	Korea	<5-HCC/PLC/liver cancer	age, platelet count, and categorized LS,together with AST level (>34 IU/mL)
	**LS-Based Model 1**	**D**	-	Severance Hospital, Samsung Medical Center, and Gangnam Severance Hospital	2012	2020	-	60.7 months	patients with NAFLD	Korea	<5-HCC/PLC/liver cancer	age, platelet count, and categorized LS,together with AST level (>34 IU/mL)
	**LS-Based Model 1**	**D**	-	Severance Hospital, Samsung Medical Center, and Gangnam Severance Hospital	2012	2020	-	60.7 months	patients with NAFLD	Korea	5-HCC/PLC/liver cancer	age, platelet count, and categorized LS,together with AST level (>34 IU/mL)
	**LS-Based Model 2**	**D**	-	Severance Hospital, Samsung Medical Center, and Gangnam Severance Hospital	2012	2020	-	60.7 months	patients with NAFLD	Korea	5-HCC/PLC/liver cancer	age, platelet count, and categorized LS,together with AST level (>34 IU/mL)
	**LS-Based Model 2**	**D**	-	Severance Hospital, Samsung Medical Center, and Gangnam Severance Hospital	2012	2020	-	60.7 months	patients with NAFLD	Korea	<5-HCC/PLC/liver cancer	age, platelet count, and categorized LS,together with AST level (>34 IU/mL)
	**LS-Based Model 2**	**D**	-	Severance Hospital, Samsung Medical Center, and Gangnam Severance Hospital	2012	2020	-	60.7 months	patients with NAFLD	Korea	<5-HCC/PLC/liver cancer	age, platelet count, and categorized LS,together with AST level (>34 IU/mL)

10.3390/cancers13184567	LS-Based Model 2	D	-	Severance Hospital, Samsung Medical Center, and Gangnam Severance Hospital	2012	2020	-	60.7 months	patients with NAFLD	Korea	5-HCC/PLC/liver cancer	age, platelet count, and categorized LS,together with AST level (>34 IU/mL)
	LS-Based Model 3	D	-	Severance Hospital, Samsung Medical Center, and Gangnam Severance Hospital	2012	2020	-	60.7 months	patients with NAFLD	Korea	5-HCC/PLC/liver cancer	age, platelet count, and categorized LS,together with AST level (>34 IU/mL)
	LS-Based Model 3	D	-	Severance Hospital, Samsung Medical Center, and Gangnam Severance Hospital	2012	2020	-	60.7 months	patients with NAFLD	Korea	<5-HCC/PLC/liver cancer	age, platelet count, and categorized LS,together with AST level (>34 IU/mL)
	LS-Based Model 3	D	-	Severance Hospital, Samsung Medical Center, and Gangnam Severance Hospital	2012	2020	-	60.7 months	patients with NAFLD	Korea	<5-HCC/PLC/liver cancer	age, platelet count, and categorized LS,together with AST level (>34 IU/mL)
	LS-Based Model 3	D	-	Severance Hospital, Samsung Medical Center, and Gangnam Severance Hospital	2012	2020	-	60.7 months	patients with NAFLD	Korea	5-HCC/PLC/liver cancer	age, platelet count, and categorized LS,together with AST level (>34 IU/mL)
	LS-Based Model 1	V	Internal	Severance Hospital, Samsung Medical Center, and Gangnam Severance Hospital	2012	2020	-	60.7 months	patients with NAFLD	Korea	5-HCC/PLC/liver cancer	age, platelet count, and categorized LS,together with AST level (>34 IU/mL)
	LS-Based Model 1	V	Internal	Severance Hospital, Samsung Medical Center, and Gangnam Severance Hospital	2012	2020	-	60.7 months	patients with NAFLD	Korea	<5-HCC/PLC/liver cancer	age, platelet count, and categorized LS,together with AST level (>34 IU/mL)
	LS-Based Model 1	V	Internal	Severance Hospital, Samsung Medical Center, and Gangnam Severance Hospital	2012	2020	-	60.7 months	patients with NAFLD	Korea	<5-HCC/PLC/liver cancer	age, platelet count, and categorized LS,together with AST level (>34 IU/mL)
	LS-Based Model 1	V	Internal	Severance Hospital, Samsung Medical Center, and Gangnam Severance Hospital	2012	2020	-	60.7 months	patients with NAFLD	Korea	5-HCC/PLC/liver cancer	age, platelet count, and categorized LS,together with AST level (>34 IU/mL)
	LS-Based Model 2	V	Internal	Severance Hospital, Samsung Medical Center, and Gangnam Severance Hospital	2012	2020	-	60.7 months	patients with NAFLD	Korea	5-HCC/PLC/liver cancer	age, platelet count, and categorized LS,together with AST level (>34 IU/mL)
	LS-Based Model 2	V	Internal	Severance Hospital, Samsung Medical Center, and Gangnam Severance Hospital	2012	2020	-	60.7 months	patients with NAFLD	Korea	<5-HCC/PLC/liver cancer	age, platelet count, and categorized LS,together with AST level (>34 IU/mL)
	LS-Based Model 2	V	Internal	Severance Hospital, Samsung Medical Center, and Gangnam Severance Hospital	2012	2020	-	60.7 months	patients with NAFLD	Korea	<5-HCC/PLC/liver cancer	age, platelet count, and categorized LS,together with AST level (>34 IU/mL)
	LS-Based Model 2	V	Internal	Severance Hospital, Samsung Medical Center, and Gangnam Severance Hospital	2012	2020	-	60.7 months	patients with NAFLD	Korea	5-HCC/PLC/liver cancer	age, platelet count, and categorized LS,together with AST level (>34 IU/mL)
	LS-Based Model 3	V	Internal	Severance Hospital, Samsung Medical Center, and Gangnam Severance Hospital	2012	2020	-	60.7 months	patients with NAFLD	Korea	5-HCC/PLC/liver cancer	age, platelet count, and categorized LS,together with AST level (>34 IU/mL)
	LS-Based Model 3	V	Internal	Severance Hospital, Samsung Medical Center, and Gangnam Severance Hospital	2012	2020	-	60.7 months	patients with NAFLD	Korea	<5-HCC/PLC/liver cancer	age, platelet count, and categorized LS,together with AST level (>34 IU/mL)
	LS-Based Model 3	V	Internal	Severance Hospital, Samsung Medical Center, and Gangnam Severance Hospital	2012	2020	-	60.7 months	patients with NAFLD	Korea	<5-HCC/PLC/liver cancer	age, platelet count, and categorized LS,together with AST level (>34 IU/mL)
	LS-Based Model 3	V	Internal	Severance Hospital, Samsung Medical Center, and Gangnam Severance Hospital	2012	2020	-	60.7 months	patients with NAFLD	Korea	5-HCC/PLC/liver cancer	age, platelet count, and categorized LS,together with AST level (>34 IU/mL)
	LS-Based Model 1	V	External	Kyungpook National University Hospital	-	-	-	28.2 months	patients with NAFLD	Korea	5-HCC/PLC/liver cancer	age, platelet count, and categorized LS,together with AST level (>34 IU/mL)
	LS-Based Model 1	V	External	Kyungpook National University Hospital	-	-	-	28.2 months	patients with NAFLD	Korea	<5-HCC/PLC/liver cancer	age, platelet count, and categorized LS,together with AST level (>34 IU/mL)
	LS-Based Model 1	V	External	Kyungpook National University Hospital	-	-	-	28.2 months	patients with NAFLD	Korea	<5-HCC/PLC/liver cancer	age, platelet count, and categorized LS,together with AST level (>34 IU/mL)
	LS-Based Model 1	V	External	Kyungpook National University Hospital	-	-	-	28.2 months	patients with NAFLD	Korea	5-HCC/PLC/liver cancer	age, platelet count, and categorized LS,together with AST level (>34 IU/mL)
	LS-Based Model 2	V	External	Kyungpook National University Hospital	-	-	-	28.2 months	patients with NAFLD	Korea	5-HCC/PLC/liver cancer	age, platelet count, and categorized LS,together with AST level (>34 IU/mL)
	LS-Based Model 2	V	External	Kyungpook National University Hospital	-	-	-	28.2 months	patients with NAFLD	Korea	<5-HCC/PLC/liver cancer	age, platelet count, and categorized LS,together with AST level (>34 IU/mL)

	LS-Based Model 2	V	External	Kyungpook National University Hospital	-	-	-	28.2 months	patients with NAFLD	Korea	<5-HCC/PLC/liver cancer	age, platelet count, and categorized LS,together with AST level (>34 IU/mL)
	LS-Based Model 2	V	External	Kyungpook National University Hospital	-	-	-	28.2 months	patients with NAFLD	Korea	5-HCC/PLC/liver cancer	age, platelet count, and categorized LS,together with AST level (>34 IU/mL)
	LS-Based Model 3	V	External	Kyungpook National University Hospital	-	-	-	28.2 months	patients with NAFLD	Korea	5-HCC/PLC/liver cancer	age, platelet count, and categorized LS,together with AST level (>34 IU/mL)
	LS-Based Model 3	V	External	Kyungpook National University Hospital	-	-	-	28.2 months	patients with NAFLD	Korea	<5-HCC/PLC/liver cancer	age, platelet count, and categorized LS,together with AST level (>34 IU/mL)
	LS-Based Model 3	V	External	Kyungpook National University Hospital	-	-	-	28.2 months	patients with NAFLD	Korea	<5-HCC/PLC/liver cancer	age, platelet count, and categorized LS,together with AST level (>34 IU/mL)
	LS-Based Model 3	V	External	Kyungpook National University Hospital	-	-	-	28.2 months	patients with NAFLD	Korea	5-HCC/PLC/liver cancer	age, platelet count, and categorized LS,together with AST level (>34 IU/mL)
10.1111/apt.15082	LCR1	D	-	Groupe Hospitalier Pitié Salpêtrière cohort	1997	2012	-	-	general people	France	10-HCC/PLC/liver cancer	hepatoprotective proteins (apolipoproteinA1, haptoglobin) ， gender, age, gammaglutamyltranspeptidase, marker of fibrosis (alpha2-macroglobulin)
	LCR1	V	Internal	Groupe Hospitalier Pitié Salpêtrière cohort	1997	2012	-	-	general people	France	10-HCC/PLC/liver cancer	hepatoprotective proteins (apolipoproteinA1, haptoglobin) ， gender, age, gammaglutamyltranspeptidase, marker of fibrosis (alpha2-macroglobulin)
	LCR1	D+V	-	Groupe Hospitalier Pitié Salpêtrière cohort	1997	2012	-	-	general people	France	10-HCC/PLC/liver cancer	hepatoprotective proteins (apolipoproteinA1, haptoglobin) ， gender, age, gammaglutamyltranspeptidase, marker of fibrosis (alpha2-macroglobulin)
	LCR2	D	-	Groupe Hospitalier Pitié Salpêtrière cohort	1997	2012	-	-	general people	France	5-HCC/PLC/liver cancer	hepatoprotective proteins (apolipoproteinA1, haptoglobin) ， gender, age, gammaglutamyltranspeptidase, marker of fibrosis (alpha2-macroglobulin)
	LCR2	V	Internal	Groupe Hospitalier Pitié Salpêtrière cohort	1997	2012	-	-	general people	France	5-HCC/PLC/liver cancer	hepatoprotective proteins (apolipoproteinA1, haptoglobin) ， gender, age, gammaglutamyltranspeptidase, marker of fibrosis (alpha2-macroglobulin)
	LCR2	D	-	Groupe Hospitalier Pitié Salpêtrière cohort	1997	2012	-	-	general people	France	5-HCC/PLC/liver cancer	hepatoprotective proteins (apolipoproteinA1, haptoglobin) ， gender, age, gammaglutamyltranspeptidase, marker of fibrosis (alpha2-macroglobulin)
	LCR2	V	Internal	Groupe Hospitalier Pitié Salpêtrière cohort	1997	2012	-	-	general people	France	5-HCC/PLC/liver cancer	hepatoprotective proteins (apolipoproteinA1, haptoglobin) ， gender, age, gammaglutamyltranspeptidase, marker of fibrosis (alpha2-macroglobulin)
10.2147/JHC.S341045	Elaborate Base Model	D	-	NIH-AARP Diet and Health Study Cohort	1995	1996	2011	16 years	general people	USA	>10-HCC/PLC/liver cancer	age, sex, BMI , height ， diabetes, general health status (excellent/very good, good, fair/poor), alcohol, moderate-to-vigorous physical activity (< 3 times/week, ≥3 times/week), dietary cholesterol, saturated fat, trans-fatty acids, ounce equivalents of lean meat from eggs per day, dietary vitamin B6, and healthy eating index scores
	Elaborate Base Model	V	Internal	NIH-AARP Diet and Health Study Cohort	1995	1996	2011	16 years	general people	USA	>10-HCC/PLC/liver cancer	age, sex, BMI , height ， diabetes, general health status (excellent/very good, good, fair/poor), alcohol, moderate-to-vigorous physical activity (< 3 times/week, ≥3 times/week), dietary cholesterol, saturated fat, trans-fatty acids, ounce equivalents of lean meat from eggs per day, dietary vitamin B6, and healthy eating index scores
	Parsimonious Base Mode	D	-	NIH-AARP Diet and Health Study Cohort	1995	1996	2011	16 years	general people	USA	>10-HCC/PLC/liver cancer	age, sex, BMI, diabetes, general health status, alcohol, moderate-to-vigorous physical activity, ounce equivalents of lean meat from eggs per day, and healthy eating index scores.
	Parsimonious Base Mode	V	Internal	NIH-AARP Diet and Health Study Cohort	1995	1996	2011	16 years	general people	USA	>10-HCC/PLC/liver cancer	age, sex, BMI, diabetes, general health status, alcohol, moderate-to-vigorous physical activity, ounce equivalents of lean meat from eggs per day, and healthy eating index scores.
	Elaborate Base Model	V	Internal	NIH-AARP Diet and Health Study Cohort	1995	1996	2011	16 years	general people	USA	>10-HCC/PLC/liver cancer	age, sex, BMI , height ， diabetes, general health status (excellent/very good, good, fair/poor), alcohol, moderate-to-vigorous physical activity (< 3 times/week, ≥3 times/week), dietary cholesterol, saturated fat, trans-fatty acids, ounce equivalents of lean meat from eggs per day, dietary vitamin B6, and healthy eating index scores
	Elaborate Base Model	V	Internal	NIH-AARP Diet and Health Study Cohort	1995	1996	2011	16 years	general people	USA	>10-HCC/PLC/liver cancer	age, sex, BMI , height ， diabetes, general health status (excellent/very good, good, fair/poor), alcohol, moderate-to-vigorous physical activity (< 3 times/week, ≥3 times/week), dietary cholesterol, saturated fat, trans-fatty acids, ounce equivalents of lean meat from eggs per day, dietary vitamin B6, and healthy eating index scores
10.1016/j.jhep.2013.09.029	CU-HCC	V	External	Prince of Wales Hospital	2006	2008	-	69 months	HBV infected	Hong Kong	<5-HCC/PLC/liver cancer	age, albumin, bilirubin, HBV DNA,and clinical cirrhosis;
	LSM-HCC	D	-	Prince of Wales Hospital	2007	2009	-	69 months	HBV infected	Hong Kong	<5-HCC/PLC/liver cancer	age, albumin, Liver stiffness measurement, HBV DNA,
	CU-HCC	V	External	Prince of Wales Hospital	2008	2010	-	69 months	HBV infected	Hong Kong	5-HCC/PLC/liver cancer	age, albumin, bilirubin, HBV DNA,and clinical cirrhosis;
	LSM-HCC	D	-	Prince of Wales Hospital	2009	2011	-	69 months	HBV infected	Hong Kong	5-HCC/PLC/liver cancer	age, albumin, Liver stiffness measurement, HBV DNA,
	CU-HCC	V	External	Prince of Wales Hospital	2010	2012	-	70 months	HBV infected	Hong Kong	<5-HCC/PLC/liver cancer	age, albumin, bilirubin, HBV DNA,and clinical cirrhosis;
	LSM-HCC	V	Internal	Prince of Wales Hospital	2011	2013	-	70 months	HBV infected	Hong Kong	<5-HCC/PLC/liver cancer	age, albumin, Liver stiffness measurement, HBV DNA,
	CU-HCC	V	External	Prince of Wales Hospital	2012	2014	-	70 months	HBV infected	Hong Kong	5-HCC/PLC/liver cancer	age, albumin, bilirubin, HBV DNA,and clinical cirrhosis;
	LSM-HCC	V	Internal	Prince of Wales Hospital	2013	2015	-	70 months	HBV infected	Hong Kong	5-HCC/PLC/liver cancer	age, albumin, Liver stiffness measurement, HBV DNA,
10.1016/j.jhepr.2021.100298	LCR1;LCR2	V	External	ANRS CO22 Hepather cohort	2012	2015	2020	-	HCV infected	France	5-HCC/PLC/liver cancer	-
	LCR1;LCR2	V	External	ANRS CO22 Hepather cohort	2012	2015	2020	-	HCV infected	France	10-HCC/PLC/liver cancer	-
	LCR1;LCR2	V	External	ANRS CO22 Hepather cohort	2012	2015	2020	-	HCV infected	France	5~10-HCC/PLC/liver cancer	-
	ANN-model 1	D	-	NHIRD	2000	2003	2009	-	patients with diabetes	Taiwan	5~10-HCC/PLC/liver cancer	sex, age,alcoholic cirrhosis, nonalcoholic cirrhosis, alcoholic hepatitis, viral hepatitis, other types of chronic hepatitis, alcoholic fatty liver disease, other types of fatty liver disease, and hyperlipidemia.
	LR-model 1	D	-	NHIRD	2000	2003	2009	-	patients with diabetes	Taiwan	5~10-HCC/PLC/liver cancer	sex, age,alcoholic cirrhosis, nonalcoholic cirrhosis, alcoholic hepatitis, viral hepatitis, other types of chronic hepatitis, alcoholic fatty liver disease, other types of fatty liver disease, and hyperlipidemia.

10.1016/j.cmpb.2015.11.009	ANN-model 2	D	-	NHIRD	2000	2003	2009	-	patients with diabetes	Taiwan	5~10-HCC/PLC/liver cancer	sex, age,alcoholic cirrhosis, alcoholic hepatitis, alcoholic fatty liver disease
	LR-model 2	D	-	NHIRD	2000	2003	2009	-	patients with diabetes	Taiwan	5~10-HCC/PLC/liver cancer	sex, age,alcoholic cirrhosis, alcoholic hepatitis, alcoholic fatty liver disease
	ANN-model 3	D	-	NHIRD	2000	2003	2009	-	patients with diabetes	Taiwan	5~10-HCC/PLC/liver cancer	sex, age, nonalcoholic cirrhosis, viral hepatitis, other types of chronic hepatitis, other types of fatty liver disease, and hyperlipidemia.
	LR-model 3	D	-	NHIRD	2000	2003	2009	-	patients with diabetes	Taiwan	5~10-HCC/PLC/liver cancer	sex, age, nonalcoholic cirrhosis, viral hepatitis, other types of chronic hepatitis, other types of fatty liver disease, and hyperlipidemia.
10.1111/liv.12621	LSPS	D	-	Consecutive patients with CHB who underwent both LB and transient elastography on the same day from January 2006 to February 2010 were identified from the database of Severance Hospital, Yonsei University College of Medicine, Seoul, Korea	2010	2016	-	-	HBV infected	South Korea	5-HCC/PLC/liver cancer	Liver stiffness, spleen diameter, platelet count
	ASPRI	V	External	Consecutive patients with CHB who underwent both LB and transient elastography on the same day from January 2006 to February 2010 were identified from the database of Severance Hospital, Yonsei University College of Medicine, Seoul, Korea	2010	2016	-	-	HBV infected	South Korea	5-HCC/PLC/liver cancer	-
	LS	V	External	Consecutive patients with CHB who underwent both LB and transient elastography on the same day from January 2006 to February 2010 were identified from the database of Severance Hospital, Yonsei University College of Medicine, Seoul, Korea	2010	2016	-	-	HBV infected	South Korea	5-HCC/PLC/liver cancer	Liver stiffness (LS) value using transient elastography
	FIB-4	V	External	Consecutive patients with CHB who underwent both LB and transient elastography on the same day from January 2006 to February 2010 were identified from the database of Severance Hospital, Yonsei University College of Medicine, Seoul, Korea	2010	2016	-	-	HBV infected	South Korea	5-HCC/PLC/liver cancer	Platelets, age, aspartate aminotransferase (AST), ALT
	APRI	V	External	Consecutive patients with CHB who underwent both LB and transient elastography on the same day from January 2006 to February 2010 were identified from the database of Severance Hospital, Yonsei University College of Medicine, Seoul, Korea	2010	2016	-	-	HBV infected	South Korea	5-HCC/PLC/liver cancer	AST, platelet count
10.1111/liv.13489	APRI	V	External	This is a retrospective cohort study of chronic HBV-infected patients who received care at Samsung Medical Center in Seoul, Korea	2006	2011	2016	-	HBV infected	South Korea	5-HCC/PLC/liver cancer	AST, platelet count
	FIB-4	V	External	This is a retrospective cohort study of chronic HBV-infected patients who received care at Samsung Medical Center in Seoul, Korea	2006	2011	2016	-	HBV infected	South Korea	5-HCC/PLC/liver cancer	Platelets, age, aspartate aminotransferase (AST), ALT

	**APRI+FIB4**	**V**	**External**	This is a retrospective cohort study of chronic HBV-infected patients who received care at Samsung Medical Center in Seoul, Korea	2006	2011	2016	-	HBV infected	South Korea	5-HCC/PLC/liver cancer	AST, platelets, age, ALT
	**CU-HCC**	**V**	**External**	Severance Hospital, Yonsei University College of Medicine (Seoul, Republic of Korea	2006	2011	-	-	HBV infected	South Korea	<5-HCC/PLC/liver cancer	Age ,Albumin, Bilirubin, HBV DNA, Cirrhosis
	**GAG-HCC**	**V**	**External**	Severance Hospital, Yonsei University College of Medicine (Seoul, Republic of Korea	2006	2011	-	-	HBV infected	South Korea	<5-HCC/PLC/liver cancer	Sex,Age ,HBV DNA , Core promoter mutations, Cirrhosis
	**REACH-B**	**V**	**External**	Severance Hospital, Yonsei University College of Medicine (Seoul, Republic of Korea	2006	2011	-	-	HBV infected	South Korea	<5-HCC/PLC/liver cancer	Sex ,Age , ALT , HBeAg,HBV DNA
	**LSM-HCC**	**V**	**External**	Severance Hospital, Yonsei University College of Medicine (Seoul, Republic of Korea	2006	2011	-	-	HBV infected	South Korea	<5-HCC/PLC/liver cancer	Age , Albumin, HBV DNA ,Liver stiffness
	**mREACH-B**	**V**	**External**	Severance Hospital, Yonsei University College of Medicine (Seoul, Republic of Korea	2006	2011	-	-	HBV infected	South Korea	<5-HCC/PLC/liver cancer	Sex ,Age ,ALT ,HBeAg ,Liver stiffness
	**CU-HCC**	**V**	**External**	Severance Hospital, Yonsei University College of Medicine (Seoul, Republic of Korea	2006	2011	-	-	HBV infected	South Korea	5-HCC/PLC/liver cancer	Age ,Albumin, Bilirubin, HBV DNA, Cirrhosis
	**GAG-HCC**	**V**	**External**	Severance Hospital, Yonsei University College of Medicine (Seoul, Republic of Korea	2006	2011	-	-	HBV infected	South Korea	5-HCC/PLC/liver cancer	Sex,Age ,HBV DNA , Core promoter mutations, Cirrhosis
	**REACH-B**	**V**	**External**	Severance Hospital, Yonsei University College of Medicine (Seoul, Republic of Korea	2006	2011	-	-	HBV infected	South Korea	5-HCC/PLC/liver cancer	Sex ,Age , ALT , HBeAg,HBV DNA
	**LSM-HCC**	**V**	**External**	Severance Hospital, Yonsei University College of Medicine (Seoul, Republic of Korea	2006	2011	-	-	HBV infected	South Korea	5-HCC/PLC/liver cancer	Age , Albumin, HBV DNA ,Liver stiffness
**10.1002/hep.28115**	**mREACH-B**	**V**	**External**	Severance Hospital, Yonsei University College of Medicine (Seoul, Republic of Korea	2006	2011	-	-	HBV infected	South Korea	5-HCC/PLC/liver cancer	Sex ,Age ,ALT ,HBeAg ,Liver stiffness
	**CU-HCC**	**V**	**External**	Severance Hospital, Yonsei University College of Medicine (Seoul, Republic of Korea	2006	2011	-	-	HBV infected	South Korea	<5-SLD/advanced liver disease/cirrhosis/liver death	Age ,Albumin, Bilirubin, HBV DNA, Cirrhosis
	**GAG-HCC**	**V**	**External**	Severance Hospital, Yonsei University College of Medicine (Seoul, Republic of Korea	2006	2011	-	-	HBV infected	South Korea	<5-SLD/advanced liver disease/cirrhosis/liver death	Sex,Age ,HBV DNA , Core promoter mutations, Cirrhosis
	**REACH-B**	**V**	**External**	Severance Hospital, Yonsei University College of Medicine (Seoul, Republic of Korea	2006	2011	-	-	HBV infected	South Korea	<5-SLD/advanced liver disease/cirrhosis/liver death	Sex ,Age , ALT , HBeAg,HBV DNA
	**LSM-HCC**	**V**	**External**	Severance Hospital, Yonsei University College of Medicine (Seoul, Republic of Korea	2006	2011	-	-	HBV infected	South Korea	<5-SLD/advanced liver disease/cirrhosis/liver death	Age , Albumin, HBV DNA ,Liver stiffness

	**mREACH-B**	**V**	**External**	Severance Hospital, Yonsei University College of Medicine (Seoul, Republic of Korea	2006	2011	-	-	HBV infected	South Korea	<5-SLD/advanced liver disease/cirrhosis/liver death	Sex ,Age ,ALT ,HBeAg ,Liver stiffness
	**CU-HCC**	**V**	**External**	Severance Hospital, Yonsei University College of Medicine (Seoul, Republic of Korea	2006	2011	-	-	HBV infected	South Korea	5-SLD/advanced liver disease/cirrhosis/liver death	Age ,Albumin, Bilirubin, HBV DNA, Cirrhosis
	**GAG-HCC**	**V**	**External**	Severance Hospital, Yonsei University College of Medicine (Seoul, Republic of Korea	2006	2011	-	-	HBV infected	South Korea	5-SLD/advanced liver disease/cirrhosis/liver death	Sex,Age ,HBV DNA , Core promoter mutations, Cirrhosis
	**REACH-B**	**V**	**External**	Severance Hospital, Yonsei University College of Medicine (Seoul, Republic of Korea	2006	2011	-	-	HBV infected	South Korea	5-SLD/advanced liver disease/cirrhosis/liver death	Sex ,Age , ALT , HBeAg,HBV DNA
	**LSM-HCC**	**V**	**External**	Severance Hospital, Yonsei University College of Medicine (Seoul, Republic of Korea	2006	2011	-	-	HBV infected	South Korea	5-SLD/advanced liver disease/cirrhosis/liver death	Age , Albumin, HBV DNA ,Liver stiffness
	**mREACH-B**	**V**	**External**	Severance Hospital, Yonsei University College of Medicine (Seoul, Republic of Korea	2006	2011	-	-	HBV infected	South Korea	5-SLD/advanced liver disease/cirrhosis/liver death	Sex ,Age ,ALT ,HBeAg ,Liver stiffness
	**CU-HCC**	**V**	**External**	From 2006 to 2014, a total of 1505 CHB patients who underwent LS measurements using TE at different time points at Severance Hospital, Yonsei University College of Medicine, Seoul, Korea	2006	2014	-	-	HBV infected	South Korea	<5-HCC/PLC/liver cancer	Age ,Albumin, Bilirubin, HBV DNA, Cirrhosis
	**REACH-B**	**V**	**External**	From 2006 to 2014, a total of 1505 CHB patients who underwent LS measurements using TE at different time points at Severance Hospital, Yonsei University College of Medicine, Seoul, Korea	2006	2014	-	-	HBV infected	South Korea	<5-HCC/PLC/liver cancer	Sex ,Age , ALT , HBeAg,HBV DNA
	**LSM-HCC**	**V**	**External**	From 2006 to 2014, a total of 1505 CHB patients who underwent LS measurements using TE at different time points at Severance Hospital, Yonsei University College of Medicine, Seoul, Korea	2006	2014	-	-	HBV infected	South Korea	<5-HCC/PLC/liver cancer	Age , Albumin, HBV DNA ,Liver stiffness
	**mREACH-B**	**V**	**External**	From 2006 to 2014, a total of 1505 CHB patients who underwent LS measurements using TE at different time points at Severance Hospital, Yonsei University College of Medicine, Seoul, Korea	2006	2014	-	-	HBV infected	South Korea	<5-HCC/PLC/liver cancer	Sex ,Age ,ALT ,HBeAg ,Liver stiffness
	**CU-HCC**	**V**	**External**	From 2006 to 2014, a total of 1505 CHB patients who underwent LS measurements using TE at different time points at Severance Hospital, Yonsei University College of Medicine, Seoul, Korea	2006	2014	-	-	HBV infected	South Korea	5-HCC/PLC/liver cancer	Age ,Albumin, Bilirubin, HBV DNA, Cirrhosis

10.1111/liv.13583	**REACH-B**	**V**	**External**	From 2006 to 2014, a total of 1505 CHB patients who underwent LS measurements using TE at different time points at Severance Hospital, Yonsei University College of Medicine, Seoul, Korea	2006	2014	-	-	HBV infected	South Korea	5-HCC/PLC/liver cancer	Sex ,Age , ALT , HBeAg,HBV DNA
	**LSM-HCC**	**V**	**External**	From 2006 to 2014, a total of 1505 CHB patients who underwent LS measurements using TE at different time points at Severance Hospital, Yonsei University College of Medicine, Seoul, Korea	2006	2014	-	-	HBV infected	South Korea	5-HCC/PLC/liver cancer	Age , Albumin, HBV DNA ,Liver stiffness
	**mREACH-B**	**V**	**External**	From 2006 to 2014, a total of 1505 CHB patients who underwent LS measurements using TE at different time points at Severance Hospital, Yonsei University College of Medicine, Seoul, Korea	2006	2014	-	-	HBV infected	South Korea	5-HCC/PLC/liver cancer	Sex ,Age ,ALT ,HBeAg ,Liver stiffness
	**CU-HCC**	**V**	**External**	From 2006 to 2014, a total of 1505 CHB patients who underwent LS measurements using TE at different time points at Severance Hospital, Yonsei University College of Medicine, Seoul, Korea	2006	2014	-	-	HBV infected	South Korea	5~10-HCC/PLC/liver cancer	Age ,Albumin, Bilirubin, HBV DNA, Cirrhosis
	**REACH-B**	**V**	**External**	From 2006 to 2014, a total of 1505 CHB patients who underwent LS measurements using TE at different time points at Severance Hospital, Yonsei University College of Medicine, Seoul, Korea	2006	2014	-	-	HBV infected	South Korea	5~10-HCC/PLC/liver cancer	Sex ,Age , ALT , HBeAg,HBV DNA
	**LSM-HCC**	**V**	**External**	From 2006 to 2014, a total of 1505 CHB patients who underwent LS measurements using TE at different time points at Severance Hospital, Yonsei University College of Medicine, Seoul, Korea	2006	2014	-	-	HBV infected	South Korea	5~10-HCC/PLC/liver cancer	Age , Albumin, HBV DNA ,Liver stiffness
	**mREACH-B**	**V**	**External**	From 2006 to 2014, a total of 1505 CHB patients who underwent LS measurements using TE at different time points at Severance Hospital, Yonsei University College of Medicine, Seoul, Korea	2006	2014	-	-	HBV infected	South Korea	5~10-HCC/PLC/liver cancer	Sex ,Age ,ALT ,HBeAg ,Liver stiffness
	**CU-HCC**	**V**	**External**	From 2006 to 2014, a total of 1505 CHB patients who underwent LS measurements using TE at different time points at Severance Hospital, Yonsei University College of Medicine, Seoul, Korea	2006	2014	-	-	HBV infected	South Korea	<5-HCC/PLC/liver cancer	Age ,Albumin, Bilirubin, HBV DNA, Cirrhosis

**REACH-B**	**V**	**External**	From 2006 to 2014, a total of 1505 CHB patients who underwent LS measurements using TE at different time points at Severance Hospital, Yonsei University College of Medicine, Seoul, Korea	2006	2014	-	-	HBV infected	South Korea	<5-HCC/PLC/liver cancer	Sex ,Age , ALT , HBeAg,HBV DNA
**LSM-HCC**	**V**	**External**	From 2006 to 2014, a total of 1505 CHB patients who underwent LS measurements using TE at different time points at Severance Hospital, Yonsei University College of Medicine, Seoul, Korea	2006	2014	-	-	HBV infected	South Korea	<5-HCC/PLC/liver cancer	Age , Albumin, HBV DNA ,Liver stiffness
**mREACH-B**	**V**	**External**	From 2006 to 2014, a total of 1505 CHB patients who underwent LS measurements using TE at different time points at Severance Hospital, Yonsei University College of Medicine, Seoul, Korea	2006	2014	-	-	HBV infected	South Korea	<5-HCC/PLC/liver cancer	Sex ,Age ,ALT ,HBeAg ,Liver stiffness
**CU-HCC**	**V**	**External**	From 2006 to 2014, a total of 1505 CHB patients who underwent LS measurements using TE at different time points at Severance Hospital, Yonsei University College of Medicine, Seoul, Korea	2006	2014	-	-	HBV infected	South Korea	5-HCC/PLC/liver cancer	Age ,Albumin, Bilirubin, HBV DNA, Cirrhosis
**REACH-B**	**V**	**External**	From 2006 to 2014, a total of 1505 CHB patients who underwent LS measurements using TE at different time points at Severance Hospital, Yonsei University College of Medicine, Seoul, Korea	2006	2014	-	-	HBV infected	South Korea	5-HCC/PLC/liver cancer	Sex ,Age , ALT , HBeAg,HBV DNA
**LSM-HCC**	**V**	**External**	From 2006 to 2014, a total of 1505 CHB patients who underwent LS measurements using TE at different time points at Severance Hospital, Yonsei University College of Medicine, Seoul, Korea	2006	2014	-	-	HBV infected	South Korea	5-HCC/PLC/liver cancer	Age , Albumin, HBV DNA ,Liver stiffness
**mREACH-B**	**V**	**External**	From 2006 to 2014, a total of 1505 CHB patients who underwent LS measurements using TE at different time points at Severance Hospital, Yonsei University College of Medicine, Seoul, Korea	2006	2014	-	-	HBV infected	South Korea	5-HCC/PLC/liver cancer	Sex ,Age ,ALT ,HBeAg ,Liver stiffness
**CU-HCC**	**V**	**External**	From 2006 to 2014, a total of 1505 CHB patients who underwent LS measurements using TE at different time points at Severance Hospital, Yonsei University College of Medicine, Seoul, Korea	2006	2014	-	-	HBV infected	South Korea	5~10-HCC/PLC/liver cancer	Age ,Albumin, Bilirubin, HBV DNA, Cirrhosis

	**REACH-B**	**V**	**External**	From 2006 to 2014, a total of 1505 CHB patients who underwent LS measurements using TE at different time points at Severance Hospital, Yonsei University College of Medicine, Seoul, Korea	2006	2014	-	-	HBV infected	South Korea	5~10-HCC/PLC/liver cancer	Sex ,Age , ALT , HBeAg,HBV DNA
	**LSM-HCC**	**V**	**External**	From 2006 to 2014, a total of 1505 CHB patients who underwent LS measurements using TE at different time points at Severance Hospital, Yonsei University College of Medicine, Seoul, Korea	2006	2014	-	-	HBV infected	South Korea	5~10-HCC/PLC/liver cancer	Age , Albumin, HBV DNA ,Liver stiffness
	**mREACH-B**	**V**	**External**	From 2006 to 2014, a total of 1505 CHB patients who underwent LS measurements using TE at different time points at Severance Hospital, Yonsei University College of Medicine, Seoul, Korea	2006	2014	-	-	HBV infected	South Korea	5~10-HCC/PLC/liver cancer	Sex ,Age ,ALT ,HBeAg ,Liver stiffness
**10.1136/gutjnl-2014-309099**	**CU-HCC**	**V**	**External**	-	-	-	-	-	HBV infected	mixed ethnicities: Asian and no-Asian	<5-HCC/PLC/liver cancer	Age ,Albumin, Bilirubin, HBV DNA, Cirrhosis
	**REACH-B**	**V**	**External**	-	-	-	-	-	HBV infected	mixed ethnicities: Asian and no-Asian	<5-HCC/PLC/liver cancer	Sex ,Age , ALT , HBeAg,HBV DNA
	**NGM1-HCC**	**V**	**External**	-	-	-	-	-	HBV infected	mixed ethnicities: Asian and no-Asian	<5-HCC/PLC/liver cancer	-
	**NGM2-HCC**	**V**	**External**	-	-	-	-	-	HBV infected	mixed ethnicities: Asian and no-Asian	<5-HCC/PLC/liver cancer	-
	**GAG-HCC**	**V**	**External**	-	-	-	-	-	HBV infected	mixed ethnicities: Asian and no-Asian	<5-HCC/PLC/liver cancer	Sex,Age ,HBV DNA , Core promoter mutations, Cirrhosis
	**GAG-HCC**	**V**	**External**	-	-	-	-	-	HBV infected	mixed ethnicities: Asian and no-Asian	5-HCC/PLC/liver cancer	Sex,Age ,HBV DNA , Core promoter mutations, Cirrhosis
	**GAG-HCC**	**V**	**External**	-	-	-	-	-	HBV infected	mixed ethnicities: Asian and no-Asian	10-HCC/PLC/liver cancer	Sex,Age ,HBV DNA , Core promoter mutations, Cirrhosis
**10.1111/jvh.12727**	**PAGE-B**	**V**	**External**	-	1985	2012	-	-	HBV infected	Netherlands	10-SLD/advanced liver disease/cirrhosis/liver death	Platelets, gender, age
	**REACH-B**	**V**	**External**	-	1985	2012	-	-	HBV infected	Netherlands	10-SLD/advanced liver disease/cirrhosis/liver death	Gender,age,ALT, HBeAg status, hepatitis B virus(HBV)DNA load(copies/mL)
	**FIB-4**	**V**	**External**	-	1985	2012	-	-	HBV infected	Netherlands	10-SLD/advanced liver disease/cirrhosis/liver death	Platelets, age, aspartate aminotransferase (AST), ALT
	**Log APRI**	**V**	**External**	-	1985	2012	-	-	HBV infected	Netherlands	10-SLD/advanced liver disease/cirrhosis/liver death	Platelets,AST
	**PAGE-B + Ishak**	**V**	**External**	-	1985	2012	-	-	HBV infected	Netherlands	10-SLD/advanced liver disease/cirrhosis/liver death	Gender,age,ALT, HBeAg status, hepatitis B virus(HBV)DNA load(copies/mL)
	**GAG-HCC**	**V**	**External**	-	1985	2012	-	-	HBV infected	Netherlands	10-SLD/advanced liver disease/cirrhosis/liver death	Gender,age,HBV DNAload(copies/mL),cirrhosis(US+)
	**CU-HCC**	**V**	**External**	-	1985	2012	-	-	HBV infected	Netherlands	10-SLD/advanced liver disease/cirrhosis/liver death	Age,albumin, bilirubin, HBV DNA load(copies/mL),cirrhosis (US+)
	**Ishak fibrosis**	**V**	**External**	-	1985	2012	-	-	HBV infected	Netherlands	10-SLD/advanced liver disease/cirrhosis/liver death	Ishak fibrosis
	**PAGE-B**	**V**	**External**	-	1985	2012	-	-	HBV infected	Netherlands	10-HCC/PLC/liver cancer	Platelets, gender, age
	**REACH-B**	**V**	**External**	-	1985	2012	-	-	HBV infected	Netherlands	10-HCC/PLC/liver cancer	Gender,age,ALT, HBeAg status, hepatitis B virus(HBV)DNA load(copies/mL)
	**FIB-4**	**V**	**External**	-	1985	2012	-	-	HBV infected	Netherlands	10-HCC/PLC/liver cancer	Platelets, age, aspartate aminotransferase (AST), ALT
	**Log APRI**	**V**	**External**	-	1985	2012	-	-	HBV infected	Netherlands	10-HCC/PLC/liver cancer	Platelets,AST
	**PAGE-B + Ishak**	**V**	**External**	-	1985	2012	-	-	HBV infected	Netherlands	10-HCC/PLC/liver cancer	Gender,age,ALT, HBeAg status, hepatitis B virus(HBV)DNA load(copies/mL)
	**GAG-HCC**	**V**	**External**	-	1985	2012	-	-	HBV infected	Netherlands	10-HCC/PLC/liver cancer	Gender,age,HBV DNAload(copies/mL),cirrhosis(US+)
	**CU-HCC**	**V**	**External**	-	1985	2012	-	-	HBV infected	Netherlands	10-HCC/PLC/liver cancer	Age,albumin, bilirubin, HBV DNA load(copies/mL),cirrhosis (US+)
	**Ishak fibrosis**	**V**	**External**	-	1985	2012	-	-	HBV infected	Netherlands	10-HCC/PLC/liver cancer	Ishak fibrosis
**10.1136/gutjnl-2015-310818**	**RWS-HCC**	**D**	-	Singapore General Hospital Department of Gastroenterology and Hepatology with ethics approval.	-	-	-	-	HBV infected	Singapore	10-HCC/PLC/liver cancer	age, gender, cirrhosis,serum AFP
	**RWS-HCC**	**V**	**External**	REACH-B cohorts	-	-	-	-	HBV infected	Asia	10-HCC/PLC/liver cancer	age, gender, cirrhosis,serum AFP
	**RWS-HCC**	**V**	**External**	GAG-HCC cohort	-	-	-	-	HBV infected	Asia	10-HCC/PLC/liver cancer	age, gender, cirrhosis,serum AFP
	**RWS-HCC**	**V**	**External**	CU-HCC cohort	-	-	-	-	HBV infected	Asia	10-HCC/PLC/liver cancer	age, gender, cirrhosis,serum AFP
**10.1007/s10620-020-06762-w**	**Liver Volume Index**	**D**	-	CHC Patients	2003	2016			HCV infected	South Korea	<5-HCC/PLC/liver cancer	Volume Index, cirrhosis, GGT > 80 U/L
	**Liver Volume Index**	**D**	-	CHC Patients	2003	2016			HCV infected	South Korea	<5-HCC/PLC/liver cancer	Volume Index, cirrhosis, GGT > 80 U/L
	**Liver Volume Index**	**D**	-	CHC Patients	2003	2016			HCV infected	South Korea	5~10-HCC/PLC/liver cancer	Volume Index, cirrhosis, GGT > 80 U/L
	**Liver Volume Index**	**V**	**Internal**	CHC Patients	2003	2016			HCV infected	South Korea	<5-HCC/PLC/liver cancer	Volume Index, cirrhosis, GGT > 80 U/L
	**Liver Volume Index**	**V**	**Internal**	CHC Patients	2003	2016			HCV infected	South Korea	<5-HCC/PLC/liver cancer	Volume Index, cirrhosis, GGT > 80 U/L
	**Liver Volume Index**	**V**	**Internal**	CHC Patients	2003	2016			HCV infected	South Korea	5~10-HCC/PLC/liver cancer	Volume Index, cirrhosis, GGT > 80 U/L
